# Applying XAI to an AI-based system for candidate management to mitigate bias and discrimination in hiring

**DOI:** 10.1007/s12525-022-00600-9

**Published:** 2022-12-20

**Authors:** Lennart Hofeditz, Sünje Clausen, Alexander Rieß, Milad Mirbabaie, Stefan Stieglitz

**Affiliations:** 1 Universität Duisburg-Essen, Forsthausweg 2, 47057 Duisburg, Germany; 2 Paderborn University, Warburger Str. 100, 33098 Paderborn, Germany

**Keywords:** Explainable AI, Hiring, Bias, Discrimination, Ethics, O30

## Abstract

Assuming that potential biases of Artificial Intelligence (AI)-based systems can be identified and controlled for (e.g., by providing high quality training data), employing such systems to augment human resource (HR)-decision makers in candidate selection provides an opportunity to make selection processes more objective. However, as the final hiring decision is likely to remain with humans, prevalent human biases could still cause discrimination. This work investigates the impact of an AI-based system’s candidate recommendations on humans’ hiring decisions and how this relation could be moderated by an Explainable AI (XAI) approach. We used a self-developed platform and conducted an online experiment with 194 participants. Our quantitative and qualitative findings suggest that the recommendations of an AI-based system can reduce discrimination against older and female candidates but appear to cause fewer selections of foreign-race candidates. Contrary to our expectations, the same XAI approach moderated these effects differently depending on the context.

**Supplementary Information:**

The online version contains supplementary material available at 10.1007/s12525-022-00600-9.

## Introduction

At 99% of Fortune 500 companies, job applications are first evaluated by an applicant tracking system instead of a human being (Hu, 2019). These systems are often based on artificial intelligence (AI) and allow human resource (HR) professionals to cope with large amounts of applicant data, the pressure to give timely responses to candidates, and limited resources for finding the best talent (Mujtaba & Mahapatra, 2019; Raghavan et al., 2020). While a universally accepted definition does not exist, AI has recently been defined as “the frontier of computational advancements that references human intelligence in addressing ever more complex decision-making problems” (Berente et al., 2021, p. 1,435). Thus, AI refers to machines performing a spectrum of cognitive tasks and intelligent behavior patterns commonly associated with human intelligence (Russell & Norvig, 2016)*.* AI comprises a variety of methods, such as machine learning (ML) and rule-based symbolic logic, which differ in their complexity and suitability for different tasks (Rouse, 2020). To date, strong AI that is akin to human intelligence does not exist. The present research focuses on a type of so-called weak AI that simulates intelligent behavior in a certain area, specifically on using ML to identify suitable candidates among job applicants (Russell & Norvig, 2016).

Importantly, AI-based systems also promise to combat the pressing problem of discrimination in hiring (Quillian et al., 2017; Sánchez-Monedero et al., 2020; Zschirnt & Ruedin, 2016) by basing decisions solely on skillsets and criteria related to job requirements rather than additional information such as demographic criteria to reduce the impact of human biases (Li et al., 2021; Ochmann & Laumer, 2019). However, this process can still be challenging, as some criteria, such as social skills, are difficult to measure using an AI-based system, and it is often difficult for humans to comprehend a system’s output. While previous literature and news media have raised concerns about potential biases in AI-based systems (Barocas & Selbst, 2016; Raghavan et al., 2020), such as the preference for male candidates in Amazon’s recruitment system (Dastin, 2018), machines themselves cannot be moral or immoral. Instead, biases in the historical data used to train an AI-based system lead to biased results, referred to as “garbage in, garbage out” (Barocas & Selbst, 2016). Discrimination by AI can also result from algorithms and presentations (Kulshrestha et al., 2019; Wijnhoven & van Haren, 2021). However, AI-based systems make such biases visible and controllable and thus can not only lead to more successful hires and lower costs but also reduce discrimination and facilitate diversity in hiring (e.g., Houser, 2019; Li et al., 2021). Nonetheless, attempts by organizations as large as Amazon to automate the hiring process have failed, which indicates that humans are still needed as final decision makers (Dastin, 2018).

Yet, AI-based systems are not likely to entirely replace humans in hiring soon but rather to augment human decision-making (Ebel et al., 2021). Augmentation refers to application scenarios of AI in organizations in which “humans collaborate closely with machines to perform a task” (Raisch & Krakowski, 2021, p. 193). Therefore, augmentation can take different forms depending on, for example, whether the AI or the human agent makes the final decision (Teodorescu et al., 2021). Here, we investigate a type of augmentation where the human is the locus of the decision, that is, where the human is the final decision maker. Thus, in this scenario, the AI will not take over the task of hiring but collaborate with the human to identify suitable candidates (Raisch & Krakowski, 2021). The final decision on whom to hire remains with the human, which introduces potential barriers to realizing the potential of AI-based systems in hiring. Some people prefer to retain decision-making power and tend to be averse to the decisions and predictions of AI-based systems and similar algorithms (Berger et al., 2021; Dietvorst et al., 2015; Jussupow et al., 2020; Ochmann et al., 2021). This phenomenon occurs even if the algorithm’s predictions are better than those of humans. High self-confidence in particular has been shown to reduce the acceptance of advice from an AI-based system (Chong et al., 2022). One reason might be that the origin of recommendations made by an AI-based system is often incomprehensible (Adadi & Berrada, 2018), which makes it difficult for people to trust the underlying technology (Zhu et al., 2018). This could lead to scenarios in which an AI-based system recommends an objectively better-qualified applicant, but the human chooses another applicant nonetheless. Thus, the final human decision could still systematically disadvantage racial minorities, older and very young applicants, and female applicants (Baert, 2018). Thus, to encourage humans to follow the recommendations of AI-based systems, additional mechanisms are needed. Accordingly, we formulated the following research question (RQ):

- **RQ1:** *Given comparable candidate qualifications, how can an AI-based system’s recommendation reduce discrimination (based on the sensitive attributes race, age, gender) in hiring decisions?*

The field of explainable AI (XAI) seeks to provide better insight into how and why an AI-based system operates the way it does (Adadi & Berrada, 2018). Barredo Arrieta et al. (2020) defined XAI as follows: “Given a certain audience, an explainable Artificial Intelligence is one that produces details or reasons to make its functioning clear or easy to understand” (p. 6). XAI refers to a variety of approaches (e.g., reverse engineering) to overcome the opaque nature of some types of AI-based systems, such as deep neural networks (Guidotti et al., 2018; Meske et al., 2022). Thereby, different XAI approaches serve different purposes and should be tailored to the target audience (Barredo Arrieta et al., 2020; Meske et al., 2022). As the target audience for the system investigated in this study comprises individuals managing applicants in hiring, we adopt a type of XAI that provides users with high-level insights into how the AI-based system weighs (sensitive) candidate attributes to derive candidate recommendations. Previous research has attempted to design XAI in a more human-centered way by testing the effect of providing contextual domain knowledge, which was found to be an influencing factor on trust of and reliance on AI-based systems (Dikmen & Burns, 2022). XAI can increase users’ trust in an AI-based system’s recommendations, their knowledge about the system, and the decision task (Barredo Arrieta et al., 2020; Meske et al., 2022). As the implementation of XAI has been shown to increase trust in AI (Meske & Bunde, 2020), XAI could improve user confidence in candidate recommendations by an AI-based system (Gunning et al., 2019; Hoffman et al., 2018). Therefore, we state a second research question:

- **RQ2:** *What is the influence of explainable AI on decision-making in the context of an AI-based system’s recommendation for hiring decisions?*

Implementing XAI in AI-based systems for candidate recommendations might increase human acceptance of these recommendations and, thus, reduce discrimination in hiring. However, little empirical research, which also shows contradictory results in terms of the effect of adding XAI and transparency, is available to date (Hofeditz et al., 2021; Shin, 2021). Previous research has indicated that the human’s role is not sufficiently studied in existing explainability approaches (Adadi & Berrada, 2018). Therefore, it is also important to identify and understand the reasons for user hiring decisions on XAI-based candidate management platforms. Therefore, we pose a third research question:

- **RQ3:** *What are users’ reasons for selecting applicants on an XAI-based candidate management platform?*

To address these research questions, we developed an interactive, functional prototype that simulated an AI-based system for candidate management and evaluated the impact of XAI and AI recommendations on the selection of typically disadvantaged individuals (2 × 2 between-subjects design, N = 194). As discrimination can differ between countries, we focused on a specific country and chose a German context for our study.

The remainder of the paper is structured as follows: First, we review relevant literature on AI-based systems in hiring, biases, and discrimination in hiring processes, and XAI. In the methods section, we describe the sample, the development of the prototypical AI-based system for candidate management, and the employed questionnaires. We then present quantitative and qualitative insights from our study and discuss them in the context of the relevant literature. The paper concludes with limitations, opportunities for future research, and a short summary of the main findings.

## Related work

### AI-based systems in hiring

As previously mentioned, AI is the frontier of computational advancements and refers to machines performing a spectrum of cognitive tasks commonly associated with human intelligence, such as complex decision-making (Berente et al., 2021; Russell & Norvig, 2016). In hiring, the use of AI-based systems has been on the rise in recent years (Black & van Esch, 2020; Raghavan et al., 2020), and organizations already use various software solutions in practice for hiring workers (Li et al., 2021; Raghavan et al., 2020; Sánchez-Monedero et al., 2020). While there has been limited research on the topic (Pan et al., 2021), existing literature suggests that AI-based systems can add great value to data-intensive and time-consuming processes in hiring, such as sourcing, screening, and the assessment of potential candidates (Black & van Esch, 2020; Li et al., 2021). Although Kuncel et al. (2014) stated that humans are good at defining job characteristics and assessing candidates in job interviews, in an analysis of 17 studies on candidate screening, they found that algorithms outperform human decision-making (measured in terms of the number of above-average performing employees recruited) by more than 25% if a large number of candidates must be screened. In addition to efficiency gains, AI-based systems also promise to reduce discrimination in hiring. Li et al. (2021) interviewed HR professionals who already used AI-based systems. Their findings suggest that the automation of hiring processes reduces opportunities for introducing biases and discrimination in hiring decisions and increases the diversity of hires (Li et al., 2021). Similarly, Ochmann and Laumer (2019) conducted expert interviews in HR management and suggested that AI can be used to highlight human biases and thus result in greater objectivity in decision-making (Ochmann & Laumer, 2019).

Black and van Esch (2020) presented several real-world examples of successful implementation of AI-based systems in organizations. For example, by introducing game-based assessments and video-based assessments, Unilever reduced the required time of HR professionals per application by 75% (Feloni, 2017). Typically, these systems do not replace but rather augment human decision-making, for example, by recommending the most suitable candidates for a position. Thus, the final hiring decision remains with the human, which poses the risk that human biases might still affect the selection of candidates.

### Bias and discrimination in hiring

As AI-based systems in hiring are not expected to fully automate but instead augment decision-making, human biases might still allow discriminatory behavior. Hiring remains an area where discrimination is most common (Sánchez-Monedero et al., 2020; Zschirnt & Ruedin, 2016). Discrimination can result from a number of psychological reasons and occurs especially in contexts with limited or missing information (Fiske et al., 1991; Foschi et al., 1994; Tosi & Einbender, 1985), as is the case in hiring. When decision makers must make decisions based on limited information, they tend to rely more on a group’s average performance to judge individuals (Guryan & Charles, 2013), and the likelihood of stereotyping increases (Fiske et al., 1991; Tosi & Einbender, 1985). In addition, ambiguous information allows room for interpretation by the human decision maker, which in turn may reinforce discrimination, as the decision is then more likely to be made based on stereotypes due to the cognitive models activated in these situations (Fiske et al., 1991). Difficulty documenting or tracking decision-making in hiring can increase discrimination as unethical behavior (Petersen & Saporta, 2004). A lack of documentation often implies that discrimination cannot be proven (Sabeg & Me´haignerie, 2006), and thus, decision makers do not face negative consequences for unethical behavior. To mitigate unethical human behavior, previous research has suggested applying AI-based systems in hiring (Hofeditz et al., 2022a, 2022b; Sühr et al., 2021), as AI is already being used by some organizations to perform the preselection of applicants (Laurim et al., 2021). However, in practice, these systems are not in charge of making the final decision (without a human decision maker). What AI-based systems usually do is provide recommendations to augment human decision-making in organizations that target in a certain direction. XAI in combination with the provision of domain knowledge can help increase trust in AI-based systems (Dikmen & Burns, 2022). With AI-based recommendations, we assume that XAI both challenges human assumptions and augments human decision-making by providing information that the human otherwise would not be aware of.

On the one hand, an AI-based system might encourage reflection on the (objective) reasons for selecting a candidate, especially if the candidate preferred by the human and the recommendation of the AI-based system differ (Ochmann & Laumer, 2019). On the other hand, it is important that the AI-based system’s recommendations not be discriminatory by design or based on certain data. Previous research has already focused on approaches to how AI-based systems can be applied without causing discrimination in hiring by, for example, avoiding biases in historical data (van Giffen et al., 2022). In this study, we therefore assume that AI-based systems in hiring are blind to historical demographic characteristics and increasingly provide recommendations based solely on objective criteria. Rieskamp et al. (2023) summarized different approaches that aim to mitigate AI-based systems’ discrimination by building on pre-process, in-process, post-process, and feature-selection approaches. Using a pre-process approach, historical data can be normalized for the training of the algorithm. Thus, if it can be assumed that AI-based systems in hiring increasingly embrace diversity, it is important to focus on human decision makers as the origin of discrimination.

Discrimination in hiring is highly relevant and frequently discussed in the literature (Akinlade et al., 2020; Baert et al., 2017; Neumark, 2018; Quillian et al., 2017, 2019; Zschirnt & Ruedin, 2016). A recent meta-analysis by Zschirnt and Ruedin (2016) found that candidates from minority groups must send out approximately 50% more applications to be invited for a job interview. Ameri et al. (2018) showed that applicants who indicated a disability that would not affect job performance received 26% less feedback than those not indicating a disability. Baert (2018) comprehensively evaluated empirical studies on discrimination in hiring from 2005 to 2016 and identified race, gender, age, religion, sexual orientation, disability, and physical appearance as reasons for discrimination that are sufficiently supported by the literature; age, gender, and race are the most frequently mentioned reasons for discrimination in the literature (Baert, 2018). We also found that these three forms of discrimination are the most common in online hiring, which made them the most suitable for our study (see Table 6 in Appendix 1 for an overview of reasons for discrimination).

An extensive amount of literature suggests addressing the issue of racial discrimination in hiring (Lancee, 2021; Quillian et al., 2017, 2019; Zschirnt & Ruedin, 2016). For example, Lancee (2021) found in a cross-national study that ethnic minorities have significantly lower chances of being hired. Quillian et al. (2017) suggested that the rate of discrimination in hiring against African Americans has not decreased over the past 25 years in the United States. Thus, race-based discrimination in hiring is among the most important cases needing to be considered, and action must be taken to ensure that it does not continue.

Victims of discrimination can differ among cultures and countries. Quillian et al. (2019) were able to show that racial discrimination in Germany occurs mostly against Turkish candidates. As we focused on the German context in this study, we chose job applicants with a Turkish name to test for race-based discrimination.

Building on the literature suggesting that AI-based systems can reduce discrimination in hiring, we hypothesize the following:

- **H1:** *Recommending foreign-race candidates in an AI-based system for candidate management leads to a higher rate of foreign-race candidate selection.*

Age-based discrimination is also one of the most relevant issues in hiring (Abrams et al., 2016; Baert, 2018; Lössbroek et al., 2021; Neumark et al., 2017; Zaniboni et al., 2019). Although this form of discrimination can affect both “too young” and “too old” candidates, current literature states that older applicants tend to have worse job application chances than younger applicants (Lössbroek et al., 2021; Neumark, 2021; Zaniboni et al., 2019). Reasons for this can be stereotypical perceptions of older candidates, such as poorer trainability (Richardson et al., 2013). Richardson et al. (2013) also found that applicants in the age group of 42 to 48 years are preferred and hired more frequently than older or younger applicants. There is also evidence in the literature that little work experience is more often a stereotypical perception of younger candidates (Baert et al., 2017). Therefore, it was important that the control group of candidates in this study be neither too old nor too young. Therefore, this study compared applicants who were younger (33–39 years old) or older (51–57 years old). A possible approach to reducing discrimination against older candidates in hiring could be the use of an AI-based candidate recommendation system, as previous research has examined their potential in recruiting (Mehrotra & Celis, 2021). Therefore, the following hypothesis is proposed:

- **H2:** *Recommending older candidates in an AI-based system for candidate management leads to a higher rate of older candidate selection.*

Previous literature clearly shows that men are consistently preferred over women in application processes (Baert, 2018; Carlsson & Sinclair, 2018; Kübler et al., 2018). The literature suggests that discrimination in hiring processes has led to and reinforces this gender inequity (Petersen & Togstad, 2006), and discrimination is often based on stereotypes, such as lower productivity of female applicants (González et al., 2019). Furthermore, Correll et al. (2007) found that women are penalized for motherhood in hiring due to various factors, such as being family-oriented. Another study found that female recruiters attributed more work experience to male applicants’ resumes than equal female applicants’ resumes (Cole et al., 2004), suggesting that even female recruiters discriminate against female applicants. In addition to male and female applicants, other types of gender experience discrimination in hiring (Davidson, 2016). However, this study was conducted in a yet unexplored field of research. For simplicity, the binary gender system was used to compare male and female applicants in this study. A possible approach to reducing discrimination of female candidates in hiring might be the use of an AI-based system for candidate recommendations, as gender is also a source of discrimination that has already been examined in the context of AI-based systems in previous studies (Fernández-Martínez & Fernández, 2020; Köchling et al., 2021). Therefore, we hypothesize:

- **H3:** *Recommending female candidates in an AI-based system for candidate management leads to a higher rate of female candidate selection.*

We use the term “sensitive attributes” to describe characteristics of candidates who are of older age, foreign race, or female and consider these attributes in the context of an AI-based system for candidate management.

### Explainable AI and its role in decision-making

One challenge in working with AI-based systems is that their results cannot always be easily explained or tracked (Dwivedi et al., 2021), and artificial and deep neural networks in particular have been described as a black box (Adadi & Berrada, 2018). This is a problem, especially for high-stakes or sensitive decisions such as hiring, as it is often not possible to explain why a system produced a certain result (Gunning et al., 2019; Hepenstal & McNeish, 2020; Sokol & Flach, 2020).

The general aim of implementing XAI is to disclose the behavior of the AI to users and make it comprehensible (Barredo Arrieta et al., 2020; Gunning et al., 2019). However, due to the relative newness and large quantity of XAI research, a standardized understanding and precise terminology regarding the term “XAI” and its applications are missing (Barredo Arrieta et al., 2020; Hussain et al., 2021; Meske et al., 2022).

Several recent surveys have provided an overview and categorization of technical XAI approaches (Adadi & Berrada, 2018; Gilpin et al., 2018; Guidotti et al., 2018). For example, Gilpin et al. (2018) focused on explaining deep neural architectures and propose a taxonomy consisting of three categories of XAI approaches that respectively: i) emulate the processing of the data, ii) explain the representation of data inside the network, or iii) are explanation-producing. Despite being less technically specific, the XAI type explored in this work falls most closely into the first category in that providing some form of justification for the input–output relation of the system may “build human trust in the system’s accuracy and reasonableness” (Gilpin et al., 2018, p. 86).

However, XAI is not just a technical concept but a movement, initiative, or effort in response to transparency issues related to AI-based systems (Adadi & Berrada, 2018). Similarly, Barredo Arrieta et al. (2020) stated that “any means to reduce the complexity of the model or simplify its outputs should be considered as an XAI approach” (p. 6). In selecting an appropriate XAI approach, Meske et al. (2022) argued that there are different objectives for XAI and the stakeholders for whom XAI is relevant. In this study, we focused on *users* of AI-based systems, for whom XAI can increase trust in the system’s recommendation and allow them to compare the system’s reasoning with their own. Furthermore, a main objective of XAI that we consider in this work is that users be able to learn from an AI-based system. Thus, in this research, we did not focus on a highly technical XAI approach (e.g., for explaining deep neural architectures as described by Gilpin et al., 2018) but provided users with a high-level explanation of how the AI-based system selects candidates and how it considers the sensitive attributes of the candidates. Thereby, the XAI can help users gain knowledge on diverse hiring selection decisions.

In XAI research, the term “transparency” often appears but is then not sufficiently differentiated. AI transparency and XAI have some overlap and are difficult to consider separately. Whereas AI transparency can be limited to the mere visibility of the deployment or use of an AI, XAI takes one step beyond this and aims to provide easily understandable and comprehensible explanations and derivations of the procedure and output of an AI-based system (Schmidt et al., 2020). Simplified, the relationship between XAI and transparency is that XAI is an approach or an effort made in response to a need for more transparency for stakeholders, such as decision makers, in the context of using AI-based systems (Adadi & Berra, 2018). However, in our literature research, we found that the distinction and relation between XAI and transparency is often not clearly addressed. With XAI’s goal of a higher level of transparency, the user should be enabled to better understand and assess the capabilities and limitations of an AI in advance (ante-hoc) (Lepri et al., 2018; Liao et al., 2020). This transparency through XAI can be achieved in various ways, for example, based on text or visualization (Barredo Arrieta et al., 2020).

As people tend to be averse to machines’ decisions (Dietvorst et al., 2015; Jussupow et al., 2020), and the opaque nature of AI can have a negative impact on trust (Hoffman et al., 2018), people might not trust candidate recommendations, rendering them ineffective for countering discrimination in hiring. Here, the emerging concept of XAI aiming to make AI use more transparent could be a promising method to increase trust in its recommendations (Thiebes et al., 2020).

Specifically, people tend to be cautious about technologies that are not interpretable or traceable (Barredo Arrieta et al., 2020), which could be reinforced by reports in the media stating that AI-based systems have led to discriminatory outcomes (Burke et al., 2021; Dastin, 2018). The goal of implementing XAI is to provide technical and contextual knowledge of how the underlying technology produces an output (Lepri et al., 2018; Mittelstadt et al., 2019), and XAI might also make it easier to identify and prevent unethical use of AI (Barredo Arrieta et al., 2020). We argue that XAI-induced transparency can increase reliance on the candidate recommendations of an AI-based system and result in users being more likely to follow the recommendations. However, as there are other studies indicating that providing transparency and domain knowledge can, in some cases, decrease trust of and reliance on a system (Dikmen & Burns, 2022; Hofeditz et al., 2021), it is difficult to determine if such an effect has a positive or a negative impact. Therefore, the following hypothesis is proposed:

- **H4:** *Explaining an AI-based system moderates the effect of recommending candidates in an AI-based system on the selection of candidates.*

This hypothesis is divided into three sub-hypotheses based on the sensitive attributes used:

- **H4.1–H4.3:** *Explaining an AI-based system moderates the effect of recommending foreign-race/older/female candidates in an AI-based system on the selection of foreign-race/older/female candidates.*

**Fig. 1:**
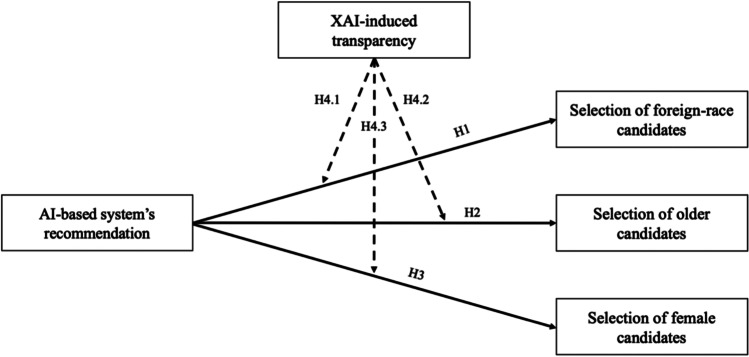
Visualization of the proposed research model

The research model is visualized in Fig. 1.

## Research design

This study implemented a 2 × 2 between-subjects design and was conducted in the form of an online experiment due to health concerns during the COVID-19 pandemic. Quantitative and qualitative data were collected with a two-part online survey and with a task on a functional, interactive platform simulating an AI-based system for candidate management. In the task, participants were asked to select suitable candidates for several job advertisements in a fictional organization.

Data collection took place between September 15, 2021, and October 20, 2021. The participants were equally distributed across four experimental groups (see Table 1). The four groups were varied in whether the participants received information about the functionality of the AI-based system (“XAI-induced transparency”) and whether candidates with a sensitive attribute (regarding age, race, gender) were explicitly recommended by the AI-based system (“AI recommendation”) on the candidate management platform. In more detail, we had AI recommendations as one varying factor and XAI-induced transparency as the other. In all groups, participants had to choose between two candidates each in 12 rounds per job and were (depending on the group) supported by AI recommendations, XAI, both, or neither. Among these 12 rounds, 6 represented the relevant rounds in which the candidates with sensitive attributes were recommended by the AI. The operationalization of our groups is explained in more detail in “Procedure.”

**Table 1:** Experimental groups

		XAI-induced transparency (No | Yes)	
AI Recommendation	No	Group 1	Group 2
	Yes	Group 3	Group 4

### Material

To investigate the impact of an AI-based system’s recommendations on human decision-making in hiring, especially for typically disadvantaged candidates, a highly controllable and customizable environment was required. Previous literature has shown that users can evaluate AI-based systems if they believe that they are interacting with one (Hofeditz et al., 2021). This approach is related to the Wizard of Oz technique in which the functionality of the system is simulated by a human (the “wizard”). This technique can be used to test the interaction between humans and intelligent systems that cannot be easily implemented or realized with available resources (Weiss et al., 2009; Wilson & Rosenberg, 1988; Schoonderwoerd et al., 2022). Here, the system’s functionality was not simulated by a human in real time but manually implemented prior to the experiment. Thus, this study did not develop a real AI-based system but a realistic, functional, and interactive prototype that simulated an AI-based system for candidate management. Specifically, we developed a candidate management platform called “nordflow” using the tool Bubble.io.^1^ The presence of the AI-based system was simulated through a cover story and various user interface elements on the platform (e.g., loading screens indicating that the AI was analyzing applications). On the platform, participants navigated between three different job advertisements, reviewed applications for the respective position, and decided which candidates to invite. With this design, we followed the recommendations of Kuncel et al. (2014), who suggested using an algorithmic system based on a large number of datapoints to narrow a field of applicants before applying a human selection process for a few selected finalists. We placed emphasis on an intuitive user interface and realism of the platform to evoke realistic responses from the participants. The procedure section provides a more detailed overview of the platform and how the participants interacted with it. We tracked both quantitative data (participant decisions) and qualitative data (participant decision rationales). The former was used for hypotheses testing and answering our research questions, and the latter to gain richer insights into the participants’ reasons for their decisions.

Furthermore, we used several questionnaires to assess different factors that might have influenced the results (Table 2). We used these controlling variables, as previous research has suggested considering a related combination in similar study contexts (Hofeditz et al., 2022a, 2022b; Mirbabaie et al., 2021a, 2021b, 2022).

**Table 2:** Questionnaires

Questionnaire	α	Author
Demographics		N/A
Big Five Inventory (BFI-10)	0.58–0.84	Rammstedt et al. (2013)
Affinity for Technology Interaction (ATI)	0.90	Franke et al. (2017)
Human Computer Trust Scale (HCTS)	0.83–0.88	Gulati et al. (2019)
NASA Task Load Index (NASA TLX)	0.83	Hart and Staveland (1988)
Ethics Position Questionnaire (EPQ)	0.80–0.83	Strack and Gennerich (2007)

The demographics questionnaire included questions on gender, age, employment status, educational attainment, and whether the participant had previous experience in HR. We then included the Affinity for Technology Interaction (ATI) scale to assess the tendency to actively engage in intensive technology interactions. The scale requires participants to rate their agreement with statements such as “I like testing the functions of new technical systems.” We included a definition of “technical systems” to ensure a common understanding. Additionally, participants were asked to answer the Human Computer Trust Scale (HCTS), which was adapted to AI and the context of hiring; for example, “I think that Artificial Intelligence is competent and effective in selecting candidates.” To ensure a common understanding of AI, we included a definition describing AI as “a system that can adapt independently to new situations and contents. It can solve problems and tasks that require a certain level of intelligence, as is typically present in humans.” To measure the subjective cognitive load after interacting with the candidate management platform, we included the NASA Task Load Index (NASA TLX), consisting of questions such as “How mentally demanding was the task?” We excluded the scales for physical and temporal demand as those were not relevant for this study. The Ethics Position Question (EPQ) was used to measure ethical dispositions by asking for agreement to items such as “Risks to another should never be tolerated, irrespective of how small the risks might be.” It was included last to avoid priming ethical behavior in the decision task. For all questionnaires, German translations or existing German versions were used, and all items were measured with a 7-point Likert scale. Following Oppenheimer et al. (2009), manipulation checks were implemented in the ATI and EPQ questionnaires to increase data quality and statistical power.

### Procedure

First, participants received general information about the study and data protection and were asked to provide their written consent. It was specified that the study could only be completed on a desktop or laptop computer, and participants could not proceed if another device was used. Afterwards, participants were asked to answer the demographics, BFI-10, ATI, and HCTS questionnaires (Table 2) and were automatically assigned to one of four experimental groups (Table 1). Then, a cover story was presented to the participants stating that a (fictional) technology organization called “nordflow” had developed an AI-based system for candidate management and that the participants would be asked to interact with a prototype of that system. The participants were informed that the AI can pre-select a certain number of suitable candidates for different job advertisements by evaluating and rating their qualifications and fit for the job advertisement (visualized with star ratings). However, the AI cannot decide between applicants with particularly similar ratings. Therefore, the participants were asked to review sets of these similarly qualified candidates, decide whom to invite for an interview, and explain their decision. Thereby, participants were asked to consider the description of the job requirements (Fig. 7 in Appendix 5) and the qualification ratings of the candidates.

**Fig. 2:**
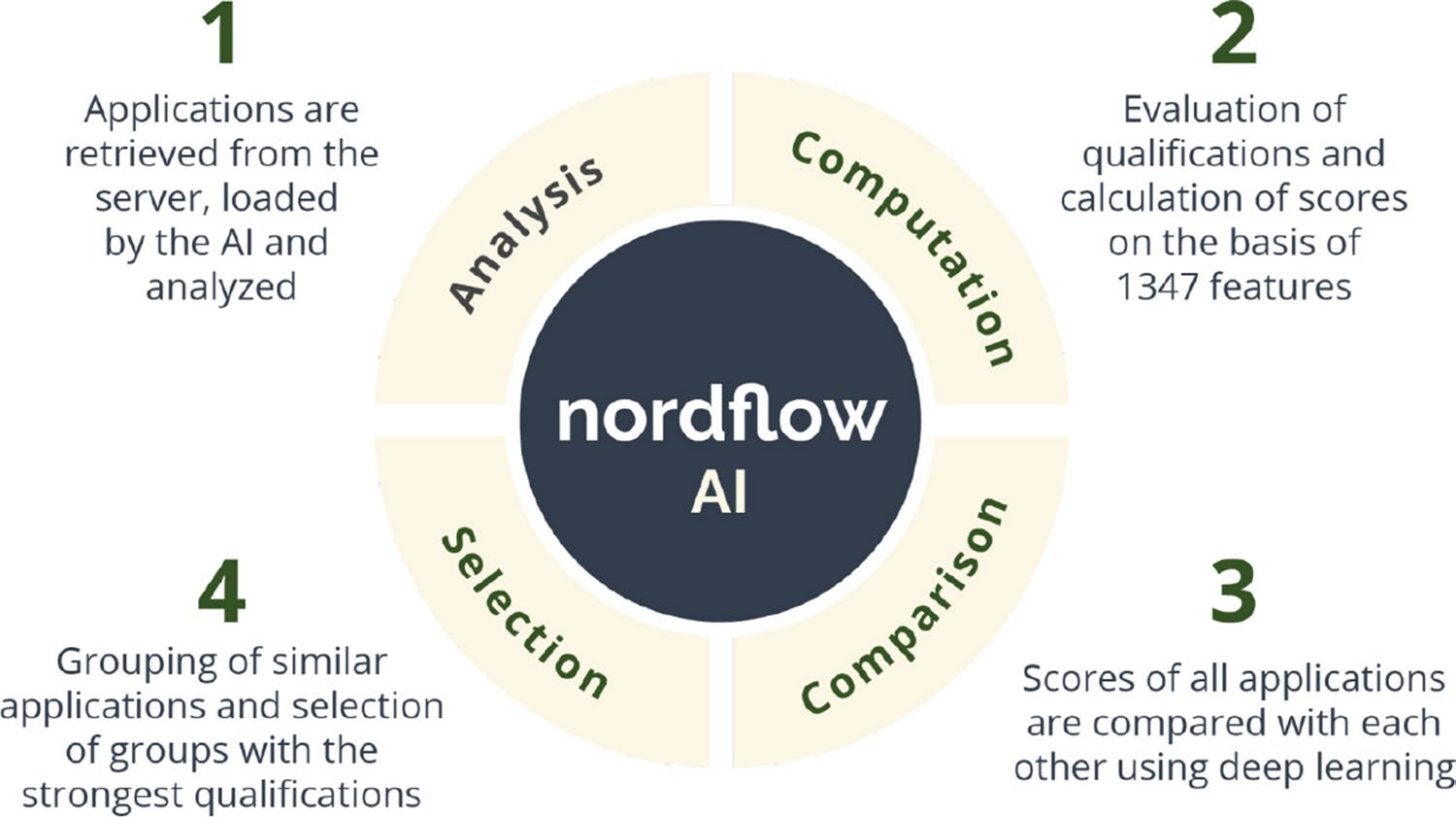
Process diagram of the candidate selection process (XAI-induced transparency)

In the experimental groups with XAI-induced transparency (groups 2 and 4; Table 1), the participants additionally received an explanation of the functionality of the AI-based system. Specifically, the participants received a description in text form and a diagram showing the candidate selection and analysis process (see Fig. 2). In the text describing the AI-based system, the participants were informed that the AI-based system uses various algorithms in its calculations. It was emphasized that in the development of the AI, an important focus was placed on diversity and that the AI differentiates applicants on a variety of characteristics selected by a panel of experts (see Appendix 4 for details). The latter highlights that the foundation of data processing has also been verified and supported by external parties, which should lead to greater trust in the AI by the participants. It was emphasized that the AI’s evaluation of candidates was based on objective criteria. Lastly, the participants were informed that the goal of the AI was to encourage decision makers to make more ethical decisions (i.e., decisions that enhance diversity) in candidate selection processes. Thus, participants in groups 2 and 4 received a high-level explanation of how the data is processed by the AI-based system, which was intended to improve their understanding of why the AI selects and recommends certain candidates over others. This type of explanation relates to the first category of XAI approaches proposed in the taxonomy of Gilpin et al. (2018) and may increase user trust in the system’s behavior. The participants in the other experimental groups did not receive this information.

Lastly, all participants were presented with a three-step tutorial explaining the platform’s functionalities and instructions for using it. This included the job view (see Fig. 7 in Appendix 5), candidate selection view (Fig. 4), and screenshot of a text field for entering the decision rationale. The tutorial was adapted to the respective experimental group. After completing the tutorial, the participants were redirected to the candidate management platform.

The “job view” of the platform showed four job advertisements, for three of which the participants should select candidates (Fig. 7 in Appendix 5). The job advertisements were identical for all participants but displayed in a randomized order. The participants were free to decide which job advertisement to start with. Each job advertisement was accompanied by a short description of the job. This description included references to different qualifications and was provided to ensure a common baseline for the participants’ assessment of the candidate’s qualifications.

**Fig. 3:**
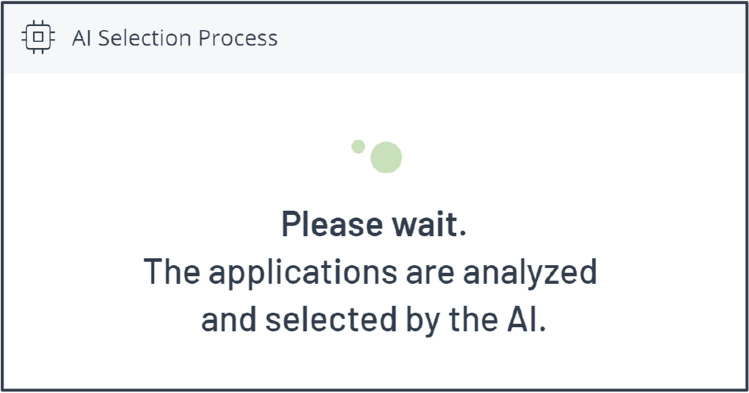
Loading screen simulating an AI-based system for candidate selection

After participants selected one of the three job advertisements by clicking on “Start Selection,” an animated loading screen appeared that served to simulate the AI-based system’s selection process (Fig. 3).

**Fig. 4:**
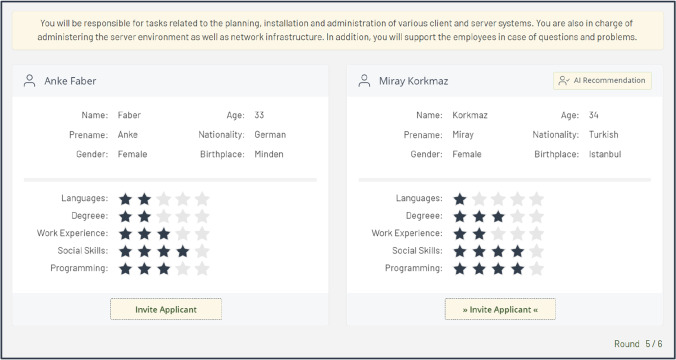
Example of the “Candidate selection view” displaying job and candidate information, qualification ratings, and (in experimental groups 3 and 4) an AI recommendation

Next, the participants saw the “candidate selection view” (Fig. 4; round 5 for the job “IT administration”), which showed the personal attributes and qualification ratings for two candidates. It was ensured that the total qualification rating (sum of stars) was identical for the two candidates. The ratings for specific qualifications differed slightly between the candidates to enhance realism, to constantly test whether participants focused on the demographics, and to examine whether AI recommendations and XAI influenced this focus. The candidates’ position, left or right, was randomized per participant to ensure that the recommendation was not always on the same side of the candidate window. Above the candidates, the description of the job advertisement was displayed as a reminder for the participants. The qualifications were visualized with a rating scale, as this makes different qualifications (e.g., different degrees) more comparable and reduces the influence of participants’ individual preferences (e.g., for a specific language).

Participants were given the task of selecting candidates for the three job advertisements with sufficient applications. For each job advertisement, participants completed six rounds, comparing two candidates per round. Of the six rounds per job advertisement, three were “relevant rounds” that included one candidate with a sensitive attribute. In total, 36 candidates were created, of which 9 were of interest for the study (one candidate each in three relevant rounds per job). A complete list of candidates is included in Table 7 in the Appendix 2. The interplay between job advertisement, sensitive attribute, and relevant rounds is displayed in Table 3.

**Table 3:** Job advertisements round sequence

Job advertisement	Sensitive attribute	Round sequence					
		1	2	3	4	5	6
1 – IT-Administration	Race	R	R	D	D	R	D
2 – Project Management	Age	D	D	R	R	D	R
3 – Accounting	Gender	D	R	D	R	R	D

*R* relevant round with a candidate with a sensitive attribute, *D* nonrelevant round

Only in experimental groups 3 and 4 were specific candidates recommended to the participant by the AI. Specifically, in the three relevant rounds per job, the candidate with a sensitive attribute was labeled with “AI Recommendation” in the upper right corner and the “invite applicant” button was complemented with small arrows (Fig. 4). In the nonrelevant rounds, the AI recommendation was “out of line,” meaning that candidates without sensitive attributes might be recommended if they were more qualified. This approach was chosen to prevent the participants from recognizing a pattern in the recommendations or candidates. The current round was displayed in the lower right corner to show the participants how far they had progressed.

**Fig. 5:**
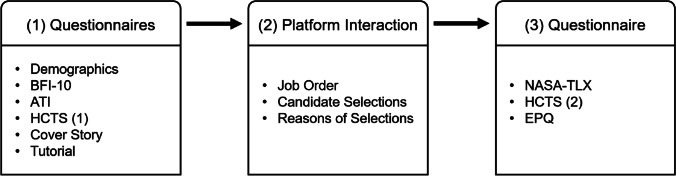
Procedure

To better understand why the participants selected a candidate, they were asked to enter the reason (as free text) for their decision after each choice. The cover story explained this to the participants by pointing out that supervisors wanted to track the reasons behind the decisions. Once the participants had completed all three job advertisements, they were directed back to the online survey. A complete overview of the order of the questionnaires, the content presented, and the information collected in this study is provided in Fig. 5.

## Findings

### Demographics

Individuals above the age of 18 years were eligible to participate in this study, and participants were recruited though SurveyCircle. Further restrictions for participation were not imposed. SurveyCircle is a research platform that helps European researchers recruit participants for online surveys and experiments. SurveyCircle’s idea is to provide the opportunity to experience current online studies and actively support research in different disciplines through voluntary participation. As with SurveyCircle a completely representative sample cannot be guaranteed, we reached out to additional participants via postings on LinkedIn, XING, and Facebook. It would be obvious to limit participation to people working in HR. However, we found that even among HR employees and within strategic HR management there are many differences between systems and HR philosophies (Lepak et al., 2004), which made it challenging to find a consistent group of HR employees while maintaining a large enough sample size. Also, employees in HR may already be aware that such a system could be used to test diversity in hiring, as this topic was already present in HR-relevant media, resulting in behavior unlike their natural decision behavior. Furthermore, AI-based systems augment people in the workplace in such a way that they can solve more complex tasks (Dellermann et al., 2019; Mirbabaie et al., 2021a, 2021b, 2021c). We therefore expect AI-based systems to enable increasingly more people in the future to perform tasks that were previously preserved by domain experts. Therefore, we decided to recruit not only current HR employees but also potential future leaders in companies as participants.

A total of 208 participants took part in the study. With 14 participants excluded for not providing reasons for their candidate selections on the interaction platform, 194 valid cases were included in the analysis. At the end of the survey, participants were asked whether they answered all the information honestly and in the best interests of the scenario (the participants were assured that their answer to this question would not put them at a disadvantage). No additional participants were excluded on the basis of this question. On average, participants spent 29 min completing the study. Participants ranged in age from 18 to 68 years (*M* = 28.28, *SD* = 9.02), of whom 126 were women (~ 65%) and 68 men. This approximates the real distribution of male and female employees working in HR in Germany, which is around 70% women and 30% men (Gorges, 2015). In addition, the sample shows that the participants were highly educated. A university degree was held by 68% of the participants, and a high school diploma or higher education entrance qualification by 23%. Furthermore, 67% of the participants reported being students, and 26% that they were employees. Between students (*M* = 25.05, *SD* = 2.99) and employees (*M* = 33.73, *SD* = 11.06), there was an age difference of almost 9 years. Moreover, among the employees, 68% reported having a university degree. Nearly one-third of the participants stated that they had experience in HR.

### Quantitative findings

#### Effect of AI-based system’s recommendations on candidate selection

To analyze whether the AI-based system’s recommendations of typically disadvantaged individuals impact candidate selection in terms of race, age, and gender (H1–H3), unpaired *t*-tests were conducted. A candidate selection score, our dependent variable, was ratio scaled, and the independent variable was categorical with two groups. Furthermore, no outliers were identified. Except for normal distribution, all requirements for the *t*-test were met. Table 4 shows the means and standard deviations of the four conditions and three job advertisements to provide an overview of the participants’ candidate selections.

**Table 4:** Participants’ candidate selections

		Job advertisement					
		Job 1		Job 2		Job 3	
No	Condition	*M*	*SD*	*M*	*SD*	*M*	*SD*
1	No recommendation and no XAI	2.11	0.759	1.91	0.905	1.45	0.829
2	No recommendation and XAI	1.81	0.970	1.89	0.759	1.47	0.830
3	Recommendation and no XAI	1.98	0.948	2.33	0.712	1.78	0.673
4	Recommendation and XAI	2.43	0.645	2.24	0.804	1.98	0.777

The relevant candidates with sensitive attributes (in terms of diversity) were coded 1, and the rest 0. The scores in Table 4 indicate (per condition and subdivided by job) the diversity in the participants’ decisions (only for the rounds with candidates of minority groups). A 3 represents a decision toward selecting more diversity (relevant candidates with sensitive attributes were chosen), and a 0 represents selection of a candidate without sensitive attributes.

We then assigned a score for each participant per job. The values in Table 4 correspond to its mean across all participants, grouped by condition. Thus, in the example of Condition 1 regarding Job 1, participants selected an average of 2.11 relevant candidates with a sensitive attribute (higher age, female, or non-German). Comparing Condition 1 with 3 in Job 1, for example, the recommendations led to a reduction in more diverse candidate selection. Table 4 provides an overview of the candidates that allows a comparison of the conditions and jobs.

Regarding race (H1), participants who received the AI-based system’s recommendations were less likely to select foreign-race candidates (*M* = 1.98, *SD* = 0.948) than those without recommendations (*M* = 2.11, *SD* = 0.759). There was no statistically significant difference between the candidate selection with recommendations and the group without recommendations, *t*(96) = 0.722, *p* = 0.472, *r* = 0.075. Thus, the first hypothesis was not supported. There was no significant effect of an AI-based system’s recommendations on the selection of foreign-race candidates.

Regarding age (H2), participants who received recommendations from the AI-based system were more likely to select older candidates (*M* = 2.33, *SD* = 0.712) than those without recommendations (*M* = 1.91, *SD* = 0.905). There was a statistically significant difference between the candidate selections with recommendations and the group without recommendations, *t*(96) = -2.555, *p* = 0.012. The effect size is *r* = 0.251 and corresponds, according to Funder and Ozer (2019), to a medium-sized effect. The second hypothesis was supported, and there was a significant positive effect of an AI-based system’s recommendations on the selection of older candidates.

Regarding gender (H3), participants who received the AI-based system’s recommendations were more likely to select female candidates (*M* = 1.78, *SD* = 0.673) than those without recommendations (*M* = 1.45, *SD* = 0.829). The Levene test did not show homogeneity of variance (*p* < 0.5). Therefore, the Welch test was conducted. There was a statistically significant difference between the candidate selections with recommendations and the group without recommendations, *t*(88.711) = -2.202, *p* = 0.03. The effect size is *r* = 0.214 and corresponds, again according to Funder and Ozer (2019), to a medium-sized effect. The third hypothesis was supported, and there was a significant positive effect of the AI-based system’s recommendations on the selection of female candidates.

In addition, moderating effects regarding gender, occupation, and HR experience were calculated using the PROCESS macro by Hayes (2018). The groups of students and employees were analyzed in terms of occupation, as they represented most of the participants. For the calculation of occupation, a new variable was calculated for each case, indicating the participant’s respective group. The independence already mentioned for the *t*-test was also required for this procedure and was present, as it resulted from the experimental design. The relationship between the variables was not linear according to a visual inspection of the scatter plot after LOESS smoothing. However, the analysis continued, and a loss of statistical power was accepted. Bootstrapping was performed with 5,000 iterations and heteroscedasticity-consistent standard errors to calculate confidence intervals (CIs) (Davidson & MacKinnon, 1993). There is no centering of variables, as only the interaction effect is of interest.

#### Effect of XAI-induced transparency on candidate selection

To analyze whether the interaction between XAI and the AI-based system’s recommendations significantly predicted participants’ candidate selections, moderation analyses using the PROCESS macro by Hayes (2018) were conducted. Bootstrapping was performed with 5,000 iterations and heteroscedasticity-consistent standard errors to calculate CIs (Davidson & MacKinnon, 1993). The relationship between the variables was not linear for any of the XAI hypotheses according to a visual inspection of the scatter plot after LOESS smoothing. However, the analysis continued, and a loss of statistical power was accepted. There is no centering of variables as only the interaction effect, the influence of XAI, is of interest. To perform the moderation analysis, two new variables were calculated from the stimulus variable that represents all four groups. Two variables were created indicating whether participants received a condition including recommendation (regardless of XAI; *n* = 100) or XAI (regardless of recommendation; *n* = 96).

The overall model regarding the selection of foreign-race candidates was significant *F*(3, 190) = 5.46, *p* = 0.001, predicting 6.88% of the variance. The moderation analysis showed that XAI significantly moderated the effect between the AI-based system’s recommendation and the selection of foreign-race candidates: Δ*R*^*2*^ = 4.66%, *F*(1, 190) = 9.33, *p* = 0.002, 95% CI[0.279, 1.236]. Thus, Hypothesis 4.1 was confirmed.

The overall model regarding the selection of older candidates was significant *F*(3, 190) = 4.04, *p* = 0.008, predicting 5.8% of the variance. However, the moderation analysis did not show that XAI significantly moderated the effect between the AI-based system’s recommendation and the selection of older candidates: Δ*R*^*2*^ < 0.01%, *F*(1, 190) = 0.084, *p* = 0.772, 95% CI[-0.518, 0.384]. Hypothesis 4.2 was not confirmed.

The overall model regarding the selection of female candidates was significant *F*(3, 190) = 4.96, *p* = 0.002, predicting 7.72% of the variance. The moderation analysis did not show that XAI significantly moderated the effect between the AI-enabled candidate recommendation system and the selection of female candidates: Δ*R*^*2*^ < 0.01%, *F*(1, 190) = 0.587, *p* = 0.444, 95% CI[-0.276, 0.613]. Hypothesis 4.3 was not confirmed.

As only one sub-hypothesis showed significance, Hypothesis 4, which states that XAI moderates the effect between an AI-based system’s recommendations and candidate selections, could not be confirmed.

**Fig. 6:**
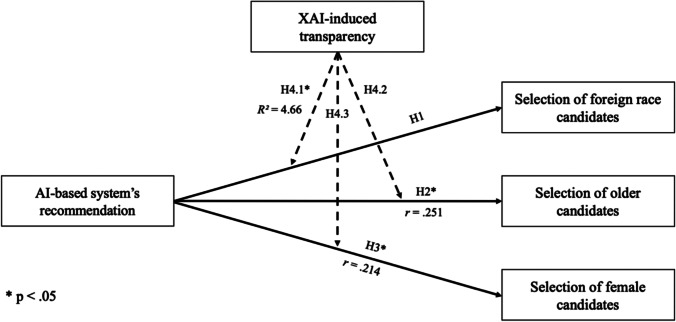
Summarized findings

**Fig. 7:**
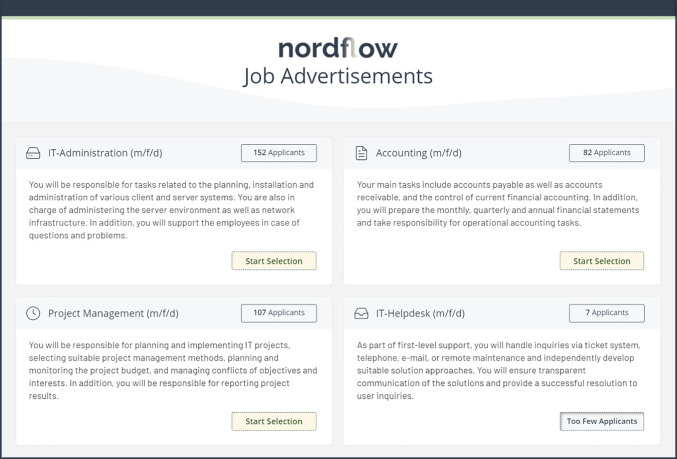
“Job view” of the platform displaying job advertisements

To summarize the findings, Fig. 6 shows the quantitative results for all hypotheses. Further results, such as an overview of the BFI-10, can be found in the Appendix.

### Qualitative findings: Reasons for participants’ selection behavior

The participants were asked the following question after each selection: “Why did you select this candidate?” Their reasons for selecting candidates on the platform is evaluated in the following sections. The dataset consists of 1,746 fields (194 participants × 3 jobs × 3 reasons), including keywords, sentences, and short argumentations. To gain insights into the reasons for selections, we conducted qualitative content analysis according to Mayring (1994).

Content analysis allowed us to summarize and reduce the participants’ reasons to their essential message. The coding categories were derived inductively and included the five different qualifications of the candidates (i.e., languages, degree, work experience, social skills, and programming/methods/software skills) and a general qualification category for those cases in which the participants did not specify which qualification contributed to the decision. Furthermore, the categories included the three sensitive attributes (i.e., race, age, gender), each with the sub-categories of “sensitive attribute preferred,” “non-sensitive attribute preferred,” or “unspecified.” Additionally, we included a category for coding whether the participants mentioned that they followed the AI recommendation. Lastly, we included the categories “subjective” for personal reasons, “ethical” for general comments about diversity without a specific reference to race, age, or gender, and “excluded” for comments that did not specify any reasons. The coding was conducted by two of the authors, who discussed the coding at several points in the process to decide on ambiguous cases. Comments could be assigned to multiple categories. Table 5 shows the derived categories with their relative occurrence in percentages.

**Table 5:** Selection reasons mentioned by participants with and without HR experience in percentages

	*L*	*D*	*WoE*	*SoS*	*P/M/S*	*Q*	*Rec*	*Race*	*Age*	*Gender*
Job 1	7.56	10.31	32.30	16.84	50.34	16.15	13.00	1.37	1.20	1.89
HR	9.70	8.48	32.73	18.79	49.70	14.55	9.80	1.82	0.00	2.42
No HR	6.71	11.03	32.12	16.07	50.60	16.79	14.65	1.20	1.68	1.68
Job 2	2.06	6.19	31.44	29.38	31.44	24.40	13.33	0.00	12.89	0.00
HR	0.61	4.85	30.30	26.06	23.03	30.91	8.82	0.00	18.18	0.00
No HR	2.64	6.71	31.89	30.70	34.77	21.82	15.66	0.00	11.03	0.00
Job 3	4.81	22.34	20.62	18.73	31.27	23.02	17.00	0.00	1.55	4.64
HR	1.82	22.42	21.21	21.21	28.48	23.03	15.69	0.00	0.00	3.03
No HR	6.00	22.30	20.38	17.75	32.37	23.02	17.68	0.00	2.16	5.28

55 participants had HR experience, 139 had no HR experience

*L* language, *D* degree, *WoE* work experience, *SoS* social skills, *P/M/S*  programming / methods / software skills, *Q *qualification, *Rec* recommendation; *Job 1 * IT Administration, *Job 2* Project Management, *Job 3* Accounting

Regarding the percentages for the sensitive attributes of race, age, and gender, all mentions of these attributes in the comments were considered. Thus, the percentage displayed includes all comments referring to a sensitive attribute, regardless of whether these are positively or negatively framed. This was done because considering these sensitive attributes in a hiring process could already be seen as a form of discrimination. For an explorative comparison, Table 5 distinguishes the reasons provided by participants with domain knowledge in HR compared to those without domain knowledge in HR.

For the first job, “IT-Administration,” approximately half the participants mentioned “programming” as a reason for selecting candidates. For the other jobs, the reasons for selection were more evenly distributed across different skills. Furthermore, while race and gender were rarely explicitly mentioned in the comments, relatively more comments addressed the age of the participants. Interestingly, more participants with HR experience mentioned age than those without HR experience. Lastly, it is interesting that participants with HR experience mentioned the AI recommendation less frequently as a reason for their selection compared to participants without HR experience.

We also examined the comments on race, age, and gender in more detail. All but one comment coded in the gender category expressed a preference for female candidates, mentioning, for example, the quota for women (ID 1771; ID 1565), that in the case of similar qualifications, women should be preferred (ID 1739), that women should be supported in certain disciplines, such as IT (ID 1848), or that a “woman is always good for team morale” (ID 1492).

With regard to race, several participants expressed a preference for non-Turkish candidates, for example, “I would not invite a Turk” (ID 1405) or “Turkish, but still more IT experience” (ID 1492) or stated “German” as the only justification for their candidate selection (ID 1385). However, one participant emphasized positive aspects of increasing diversity with new hires, stating “intercultural, therefore access to other resources” (ID 1749).

Lastly, almost all participants who commented on a candidate’s age preferred younger candidates, stating, for example, that another candidate has “more time before retiring” (ID 1395). Several participants connected age to the ability to learn quickly, which compensated for methodological skills that they were currently lacking. For example, “methodological competence can possibly be further developed due to his age” (ID 1692). The only pro-older candidate comment was “Older women should be supported” (ID 1848)..

## Discussion

### Why AI recommendations might not reduce race-based discrimination in hiring

The participants’ selection of foreign-race candidates was not in line with existing literature on racial discrimination in hiring, which has found that race continues to be one of the main sources of discrimination in hiring today and that Turkish individuals are especially discriminated against in Germany (Baert, 2018; Quillian et al., 2017, 2019; Zschirnt & Ruedin, 2016). This was generally not the case in our study, as the control group without recommendations and explainability showed a high number of selections of foreign-race candidates. However, if the AI-based system recommended a foreign-race candidate, the candidate was less likely to be selected. Thus, the AI-based system’s recommendations did not increase the selection of foreign-race candidates, and Hypothesis 1 was not supported. Instead, participants who received the recommendations tended to select fewer foreign-race candidates than participants who did not receive recommendations. As the BFI-scores of the participants in the experimental groups receiving AI recommendations did not differ significantly from the groups without AI recommendations (see Table 8 in Appendix 5), this difference does not appear to result from personality differences of the participants in the respective groups.

We suggest that the implemented AI recommendation did not work as assumed because of algorithmic aversion (Berger et al., 2021; Dietvorst et al., 2015; Ochmann et al., 2021). Although algorithmic aversion usually occurs if people with domain knowledge can select between a human and an algorithm recommendation (Dietvorst et al., 2015), some previous research has suggested that there are cases of algorithm aversion occurring even if there is no human recommender alternative (Bigman et al., 2021). The qualitative analysis also provides evidence for this, suggesting that participants with domain knowledge in HR relied less on the AI recommendations compared to participants without domain knowledge. Accordingly, there is a possibility that the participants were indeed averse to AI, which led to a rejection of the recommended candidate and an increased selection of German candidates. This could be another indicator that algorithmic aversion can occur even without offering a human alternative, as suggested by Bigman et al. (2021). To avoid possible aversion, AI-based systems might be used to some extent as part of new task designs that balance human and system characteristics through mutual delegation (Baird & Maruping, 2021). Another approach to mitigating aversion is to get affected individuals better involved in the AI adaptation process as part of organizational learning (Wijnhoven, 2021). On the other hand, the lower reliance on the AI recommendations of some of the participants might also be due to a higher degree of self-confidence in selecting the right candidate, as recent research has found that self-confidence influences the adoption or rejection of AI augmentation (Chong et al., 2022).

As the reasons provided by participants with and without HR practice differed only slightly and participants could not select between a human and an algorithmic advisor, further explanations than aversion and self-confidence need to be taken into account. Considering the qualitative results, the low number of reasons given based on a candidate’s race suggests that participants did not pay much attention to the race of candidates or that they were trying to be as objective as possible in decision-making. This could indicate that the selection decisions were, in fact, predominantly made based on qualifications. While the overall qualification (sum of stars) was identical for the candidates, the individual scores for qualifications differed slightly. However, it is also possible that participants were not aware of or avoided mentioning the role of the candidate’s race in their selection, either because they were not aware of their own biases or because they did not want to admit them (i.e., the answers might be subject to a social desirability bias).

Examining the participants’ reasons in more detail, we found that programming experience was the most frequently mentioned reason for the decision in the first job round in which race was the sensitive attribute. We therefore assume that the reason for not finding an effect of AI recommendation on selecting foreign-race candidates could be that the majority of our participants were sure that programming skills comprised the most important criterion for the role of IT administrator even though other skills were mentioned in the job description. Thus, an AI recommendation does not seem to be effective when there is already a clear qualification-based indicator for a decision. This could be explained by a high level of confidence that results in the avoidance of following AI recommendations. The result of the personality test (BFI-10) could also be used to explain the lack of evidence for discrimination in the initial candidate selection. A high level of openness (see Table 8 in Appendix 5) indicates that the participants think unconventionally and are open to new things (John & Srivastava, 1999). This could foster consideration of the overall qualification of the candidates regardless of their demographics and thus, result in less discrimination. However, we did not find systematic differences in personality between the relevant groups. When considering the results for Hypotheses 2 and 3, it becomes apparent that the AI-based system’s recommendations can also work as expected in cases where participants perceive less clear qualification-based criteria for job profiles than was the case with programming for the first job.

### AI recommendations can reduce age- and gender-based discrimination

When examining candidate selection in the control group, it becomes apparent that compared to the attributes of race and age, the participants selected female candidates considerably less often. This reinforces the evidence in the literature regarding discrimination against female candidates in hiring (Baert, 2018; Carlsson & Sinclair, 2018; Kübler et al., 2018). The qualitative data rather signaled that if participants mentioned gender as a reason for selection, they emphasized positive (yet partially stereotypical) aspects of hiring women (e.g., being good for team morale). This suggests that the negative discrimination against women shown by the quantitative results happened unconsciously or that participants deliberately concealed the discrimination.

The quantitative finding that AI recommendations can increase the selection of older and female candidates (H2 and H3) can be further strengthened by the qualitative results, which reveal that 13% and 17%, respectively, of the participants who received recommendations mentioned it as a reason for their candidate selection. In addition, participants stated that they used the recommendations to make decisions in cases of uncertainty. Regarding the second hypothesis, where we considered whether an AI-based system’s recommendations impact the selection of older candidates, it was supported and showed a medium-sized effect. This implies that the recommendations led to more frequent selection of older candidates. These findings are strengthened by the qualitative findings, in which the participants mentioned the recommendation as a reason for their selection. Furthermore, the participants’ ethical position (EPQ) indicated that they possessed rational and diverse candidate selection behavior. The findings for the sensitive attribute age (H2) are also in line with current literature regarding discrimination, which shows that older candidates are subject to discrimination in hiring (Baert, 2018; Lössbroek et al., 2021; Neumark et al., 2017; Zaniboni et al., 2019). In summary, the AI-based system’s recommendations positively influenced the participants’ selection decisions for older and female candidates.

Comparing the participants with and without HR experience, those with HR experience mentioned age more often as a reason for their decision. If we assume that a candidate was selected mainly because of their age and not because of a certain skillset, we consider this a case of age discrimination (Baert, 2018; Neumark, 2021; Richardson et al., 2013; Zaniboni et al., 2019). However, AI-based recommendations showed a positive effect on the selection of an older candidate for both groups.

### The role of XAI in AI-based systems for hiring

Our findings suggest that XAI-induced transparency, that is, providing participants information about the functionality of the AI-based system, did not moderate the effect of the system’s recommendations on the selection of older and female candidates (H4.2 and H4.3 rejected). It appears that emulating the processing of an AI-based system by providing a high-level explanation of the input–output relation of the data did not – as would be expected based on the suggestions of Gilpin et al. (2018) – increase the participants’ trust in and acceptance of the system’s recommendations. Thus, these findings seem to challenge expectations highlighting the effectiveness of and general need for XAI (Adadi & Berrada, 2018; Dwivedi et al., 2021; Gunning et al., 2019). One reason for this might be that there is a wide range of XAI types (see, e.g., Giudotti et al. (2018) for an overview) and that a different XAI type would have been more suitable to support the target audience. However, as XAI-induced transparency positively moderated the selection of foreign-race candidates (H4.1 supported), the effectiveness of XAI might also depend on the content of the decision task. Previous research has already emphasized that a successful application of XAI depends on various quality criteria, such as fidelity, generalizability, explanatory power, interpretability, comprehensibility, plausibility, effort, privacy, and fairness, depending on the target group (Meske et al., 2022). Here, with H4.2 and H4.3 not being supported and H4.1 being supported, our findings suggest that not only quality criteria and XAI type but also the content of the decision task need to be considered.

With these findings, we addressed the research gap identified by Adadi and Berrada (2018), who argued that the role of humans in XAI is inconclusive and can only be attributed to undiscovered influencing factors. We provided empirical evidence for the context of discrimination in hiring and tested XAI in the context of participants’ ethical position and personality traits. In addition, our findings suggest that the content for achieving XAI-induced transparency should be individually adaptable to user qualifications. This is in line with Shin (2021), who argued that algorithmic experience in AI needs to be addressed in practice and that heuristics and cognitive processes need to be incorporated into the design of these algorithms, making them user-centric. Furthermore, based on our findings, more research is needed regarding the mechanisms of XAI on humans and their influencing factors, which was also one of the research opportunities outlined by Meske et al. (2022) for XAI in information systems. In addition, we provided empirical evidence on how a higher degree of transparency leads to better understanding of potentially undesired practices in the offline world (e.g., gender bias and discrimination), which was mentioned as a promising research direction by Meske et al. (2022). We addressed both knowledge on XAI in the context of individual attributes and knowledge on how XAI and transparency can lead to less discrimination and bias in hiring.

### Limitations and further research

The study adopted a fairly broad, high-level type of XAI in which participants received a general explanation about the processing of data in the system as well as its goal of augmenting decision-making in hiring to reduce discrimination. However, there are many other, more technically detailed XAI approaches that could prove (more) effective in this context (see, e.g., Adadi & Berrada, 2018 or Gilpin et al., 2018). While this study focused on a target audience (and a corresponding sample) of non-AI experts, we acknowledge that this might be an insufficiently detailed characterization of HR professionals. In addition, a relatively large number of participants were educated, female, and from Germany, and only about one-third of the participants reported prior HR experience. Therefore, the findings are subject to limited generalizability to HR professionals.

The candidate management platform was designed to resemble prevalent AI-based systems for this purpose; however, the findings might not be generalizable to other platforms in this domain. The overall qualification of the candidates (sum of stars) was identical for both candidates in each round; the star ratings for specific qualifications differed between the two candidates. While this was necessary to gain insights on participants’ tendency to consider demographic information for deciding between candidates, it introduced the risk that the participants’ perceived relevance of certain qualifications for a job influenced their selection. Also, the effects might differ if participants had to select candidates from a larger pool of candidates on the platform rather than making a choice based on a direct comparison of two candidates. Lastly, the cultural context in which the platform is deployed might make a difference as for example the influence of AI recommendations could be more pronounced in highly technology-affine societies.

Addressing these limitations, future research could explore the (dis-)advantages of different types of XAI from the perspective of HR professionals in greater depth. It would be interesting to conduct the study in a real HR environment and limit participation to experienced HR employees. As the sensitive attributes leading to discrimination might differ depending on contextual factors (e.g., culture) or individual factors (e.g., characteristics of the decision maker), future studies should aim to explore the effects of AI recommendations and XAI with different sensitive attributes (e.g., disability) and a diverse group of HR professionals. Furthermore, to dive deeper into possible causes for the observed candidate selection behavior and the effectiveness of AI recommendations and XAI, future research could measure algorithmic aversion, automation bias, cognitive load, or the effect of mistrust disposition. Future research should consider directly measuring these aspects in the context of XAI and AI recommendations for candidate selection and examine possibilities to mitigate aversion, for example, by incorporating AI-based information systems as part of new task designs that balance human and systemic characteristics through mutual delegation and through organizational learning processes with strong stakeholder participation in AI adoption. Additionally, to improve generalizability, future research could investigate XAI and AI recommendations on different types of candidate management platforms and in alternative deployment contexts (e.g., other countries).

Lastly, we focused on the point of view of the recruiter and not on those who are affected by discrimination or bias in hiring. Future research needs to go a step further and, for example, follow a discourse ethics approach based on that of Mingers and Walsham (2010) by also involving other stakeholders in the debate about diversity in XAI-based recommendations.

### Contribution to research

The findings of this study contribute to research on augmenting human decision-making with AI-based systems in several ways. First, we showed that in decision-making scenarios with no clearly preferable option, providing AI recommendations and XAI can influence decision-making and potentially reduce discrimination in hiring. Second, our findings suggest that a clear association between a qualification-based criterion and a decision outcome limits the impact of AI recommendations on decision-making. Third, our exploratory analysis indicated that participants with domain knowledge did not behave differently in response to AI recommendations and/or XAI than participants without domain knowledge. Fourth, we open a new field of research regarding the combination of XAI and AI-based system recommendations to augment decision-making in the context of hiring.

We also contribute to the literature on XAI by empirically testing the influence of XAI on the effectiveness of augmenting decision making with an AI-based system in the context of hiring. As the effects of XAI differed for the sensitive attributes, our findings suggest that, in addition to quality criteria and target groups (Meske et al., 2022), the content or context of the decision plays a role in the impact of XAI.

Furthermore, this research extends the literature concerning the reduction of discrimination in hiring (e.g., Foley & Williamson, 2018; Krause et al., 2012) and presents recommendations regarding an AI-based system as a promising approach for reducing discrimination against older and female candidates in hiring. Moreover, the findings argue for the positive benefits of using AI to reduce discrimination and bias, complementing the literature that discusses the ethical issues of AI in hiring (Lepri et al., 2018; Raghavan et al., 2020). Finally, the study contributes to broadening the understanding of AI in society by demonstrating a new beneficial use case of applying XAI to reduce discrimination in hiring.

### Contribution to practice

This research also provides practical implications for stakeholder groups working with XAI, such as AI managers, AI developers, AI users, and individuals affected by AI-based system decisions and recommendations.

On the one hand, the study contributes to increasing general welfare by examining an important topic for society and electronic markets. Thus, our findings might lead to greater diversity in future workforces and positively affect individuals with sensitive attributes who are subject to AI-based system recommendations. For example, recruiters as AI users can augment their decision making with similar systems, reflect on their (potential) biases, and better understand the reasons for AI-based system recommendations through XAI. On the other hand, this research can draw the attention of organizations and AI managers to the issue of discrimination remaining an important problem in hiring. Furthermore, the platform conceptualized and developed in this research can be a starting point for developing a system for training HR staff on discrimination in hiring. XAI and recommendations from AI-based systems can be effective, but they may require further action from the organization to achieve diverse hiring in the long term.

For practical application purposes, XAI might be successful in areas where users are in more frequent contact with the technology. Complementing XAI, the implementation of AI recommendations might be a suitable method to realize the AI’s purpose of, in this case, countering discrimination in hiring. Moreover, in general, educating different stakeholders of XAI about AI’s potential dangers and benefits would be advisable to reduce prejudice and fear and increase general acceptance of AI.

From an organizational perspective, a question arises as to the overall benefits of XAI for their business. Not every organization that uses an AI-based system necessarily needs to understand the reasons for its outcomes. In addition, some algorithms must be developed from scratch to allow for the ability to explain the processes and reasons for decisions afterward. This leads to an immense amount of work, which may not justify the perceived benefits of XAI in every context. Therefore, incentives are needed that could counteract some of the barriers to the implementation of XAI to ensure more diversity and fewer biases through XAI and AI based-system decisions and recommendations.

## Conclusion

In summary, our findings suggest how recommendations by an AI-based system for hiring, combined with an XAI approach, can be applied on a candidate management platform to achieve greater transparency and diversity. It appears that AI recommendations are sufficient to cause participants to reconsider their decision-making or to draw attention to sensitive attributes. While our findings might not generalize to other AI-based systems or candidate management platforms, we found that AI recommendations encouraged decision makers to select more female and older candidates. However, the recommendations also resulted in fewer selections of foreign-race candidates, which might be due to algorithmic aversion caused by overly obvious recommendations based on sensitive attributes. Furthermore, while explainability moderated the effect of AI recommendations on the selection of foreign-race candidates, our findings cannot unreservedly support the positive impact of explainability on the effect of AI recommendations on selection behavior. However, our findings overall suggest that AI recommendations can reduce discrimination in hiring decisions. We further conclude that XAI helped reduce reactance and aversion caused by recommendations that were too obviously perceived as an influencing factor by our participants. The XAI appeared to have different effects for the same target group and the same quality criteria, highlighting the importance of considering the content of the decision task.

### Electronic supplementary material

Below is the link to the electronic supplementary material.

Supplementary file1 (PDF 483 KB)

## Appendix 1

Table 6

**Table 6:** Reasons for discrimination in hiring

Discrimination type	Disadvantaged groups	Exemplary sources
Race	Minority races and national origins	(Baert, 2018; Weichselbaumer, 2016)
Gender	Females or mothers	(Baert, 2018; Ruffle & Shtudiner, 2015)
Age	Too young or old	(Baert, 2018; Neumark, 2021; Richardson et al., 2013; Zaniboni et al., 2019)
Religion	Minority religions	(Baert, 2018; Weichselbaumer, 2016)
Disability	Any disability	(Baert, 2018; Stone & Wright, 2013)
Sexual orientation	Non-heterosexual orientation	(Baert, 2018; Weichselbaumer, 2016)
Physical appearance	Low attractiveness, females with high attractiveness	(Baert, 2018; Stone & Wright, 2013)

## Appendix 2

Table 7

**Table 7:** Candidate profiles

Job	Round	Type	Prename	Name	Gender	Age	Birthplace	Nationality	Lang	Deg	Exp	Social	Extra
1	1	0	Dirk	Bauer	Male	41	Solingen	German	3	4	3	3	2
1	1	1	Mustafa	Özdemir	Male	43	Tarsus	Turkish	2	3	4	3	3
1	2	1	Ecrin	Şahin	Female	37	Adana	Turkish	4	3	1	2	4
1	2	0	Stephanie	Jung	Female	39	Kerpen	German	3	2	2	4	3
1	3	99	Paulina	Krasny	Female	37	Breslau	Polish	1	3	2	2	4
1	3	98	Wolfgang	Fischer	Male	54	Wittlich	German	3	2	5	1	1
1	4	99	Mathias	Eisenberg	Male	42	Ulm	German	1	2	3	1	3
1	4	98	Dennis	Zimmer	Male	37	Rosenheim	German	3	4	2	3	2
1	5	1	Miray	Korkmaz	Female	34	Istanbul	Turkish	1	3	2	4	4
1	5	0	Anke	Faber	Female	33	Minden	German	2	2	3	4	3
1	6	99	Maria	van Dijk	Female	39	Venlo	Dutch	4	2	4	3	3
1	6	98	Jürgen	Fuhrmann	Male	51	Bramsche	German	2	3	3	5	2
2	1	99	Luisa	Engel	Female	56	Marburg	German	3	4	4	2	2
2	1	98	Sophia	Thalberg	Female	47	Lörrach	German	2	3	3	4	5
2	2	98	Oskar	Borkowski	Male	37	Lissa	Polish	2	3	3	1	5
2	2	99	Simone	Wulf	Female	33	Leipzig	German	5	5	1	4	3
2	3	0	Sven	Kuster	Male	33	Flensburg	German	2	4	3	2	4
2	3	1	Thomas	Ackermann	Male	54	Tübingen	German	2	2	4	4	3
2	4	1	Monika	Zimmer	Female	57	Niebüll	German	3	4	4	2	3
2	4	0	Lisa	Schaefer	Female	36	Babelsberg	German	2	5	3	4	2
2	5	98	Philipp	Neumann	Male	54	Essen	German	2	3	4	5	4
2	5	99	Katja	Weiß	Female	36	Halle (Saale)	German	4	2	3	5	2
2	6	0	Martin	Bach	Male	39	Nürnberg	German	5	3	2	4	1
2	6	1	Patrick	Lehmann	Male	51	Hilden	German	3	3	3	2	4
3	1	99	Robin	Winkler	Male	51	Wesel	German	3	5	3	2	5
3	1	98	Jannik	Grunewald	Male	48	Bocholt	German	3	2	4	3	4
3	2	0	Christian	Nacht	Male	37	Bayreuth	German	2	3	3	5	2
3	2	1	Katharina	Decker	Female	36	Celle	German	3	4	2	3	3
3	3	99	Laura	Fischer	Female	34	Paderborn	German	2	1	2	3	2
3	3	98	Aylin	Öztürk	Female	45	Mersin	Turkish	4	2	4	2	3
3	4	0	Arne	Meyer	Male	44	Halberstadt	German	4	4	4	2	3
3	4	1	Karin	Richter	Female	42	Fulda	German	3	5	4	3	2
3	5	1	Sophia	Ostermann	Female	34	Dresden	German	2	3	2	4	2
3	5	0	Dominik	Braun	Male	33	Rathenow	German	3	4	1	2	3
3	6	98	Anna	Iwanow	Female	57	Krasnojarsk	Russian	3	4	5	3	2
3	6	99	Aaron	Becker	Male	39	Koblenz	German	2	3	3	4	5

Column Type: 0 = relevant; 1 = relevant with recommendation; 98 = non-relevant with recommendation; 99 = non-relevant. Lang. = Languages; Deg. = Degree; Exp. = Work Experience; Social = Social Skills; Extra = Dynamic Field

## Appendix 3

### Questionnaires

All questionnaires are based on a 7-point Likert scale.

**Big Five Inventory (BFI-10)**

*Bitte geben Sie den Grad Ihrer Zustimmung zu folgenden Aussagen an.*

1. Ich bin eher zurückhaltend, reserviert.
2. Ich schenke anderen leicht Vertrauen, glaube an das Gute im Menschen.
3. Ich bin bequem, neige zur Faulheit.
4. Ich bin entspannt, lasse mich durch Stress nicht aus der Ruhe bringen.
5. Ich habe nur wenig künstlerisches Interesse.
6. Ich gehe aus mir heraus, bin gesellig.
7. Ich neige dazu, andere zu kritisieren.
8. Ich erledige Aufgaben gründlich.
9. Ich werde leicht nervös und unsicher.
10. Ich habe eine aktive Vorstellungskraft, bin fantasievoll.

**Affinity for Technology Interaction (ATI)**

*Bitte geben Sie den Grad Ihrer Zustimmung zu folgenden Aussagen an.*

Mit „technischen Systemen “ sind sowohl Apps und andere Software-Anwendungen als auch komplette digitale Geräte (z.B. Handy, Computer, Fernseher, Auto-Navigation) gemeint.

1. Ich beschäftige mich gern genauer mit technischen Systemen.
2. Ich probiere gern die Funktionen neuer technischer Systeme aus.
3. In erster Linie beschäftige ich mich mit technischen Systemen, weil ich muss.
4. Wenn ich ein neues technisches System vor mir habe, probiere ich es intensiv aus.
5. Ich verbringe sehr gern Zeit mit dem Kennenlernen eines neuen technischen Systems.
6. Es genügt mir, dass ein technisches System funktioniert, mir ist es egal, wie oder warum.
7. Ich versuche zu verstehen, wie ein technisches System genau funktioniert.
8. Es genügt mir, die Grundfunktionen eines technischen Systems zu kennen.
9. Ich versuche, die Möglichkeiten eines technischen Systems vollständig auszunutzen.

**Human Computer Trust Scale (HCTS)**

*Bitte geben Sie den Grad Ihrer Zustimmung zu folgenden Aussagen an.*

Eine künstliche Intelligenz (KI) lässt sich als System beschreiben, dass die Fähigkeit besitzt, sich selbstständig an neue Situationen und Inhalte anzupassen. Es kann Probleme lösen und Aufgaben erledigen, die ein gewisses Maß an Intelligenz erfordern, wie sie typischerweise bei Menschen vorhanden ist.

1. Ich glaube, dass der Einsatz einer künstlichen Intelligenz negative Folgen haben könnte.
2. Ich glaube, ich muss vorsichtig sein, wenn ich eine künstliche Intelligenz verwende.
3. Es ist riskant, mit einer künstlichen Intelligenz zu interagieren.
4. Ich glaube, dass eine künstliche Intelligenz in meinem besten Interesse handeln wird.
5. Ich glaube, dass eine künstliche Intelligenz ihr Bestes tun wird, um mir zu helfen, wenn ich Hilfe benötige.
6. Ich glaube, dass eine künstliche Intelligenz daran interessiert ist, meine Bedürfnisse und Vorlieben zu verstehen.
7. Ich denke, dass eine künstliche Intelligenz bei der Auswahl von Bewerber:innen kompetent und effektiv ist.
8. Ich denke, dass eine künstliche Intelligenz ihre Rolle als Instrument zur Bewerberauswahl sehr gut erfüllt.
9. Ich glaube, dass eine künstliche Intelligenz über alle Funktionen verfügt, die ich von einem Hilfsmittel zur Bewerberauswahl erwarten würde.
10. Wenn ich eine künstliche Intelligenz verwende, denke ich, dass ich mich vollständig auf sie verlassen kann.
11. Ich kann mich immer auf eine künstliche Intelligenz verlassen, wenn es um die Entscheidungsfindung geht.
12. Ich kann den Informationen vertrauen, die mir eine künstliche Intelligenz liefert.

**NASA Task Load Index (NASA-TLX)**

1. Wie viel geistige Anforderung war bei der Aufnahme und Verarbeitung von Informationen erforderlich? War die Aufgabe einfach oder komplex?
2. Wie erfolgreich haben Sie Ihrer Meinung nach die vom Versuchsleiter (oder Ihnen selbst) gesetzten Ziele erreicht?
3. Wie anstrengend war die Arbeit, um Ihren Grad an Aufgabenerfüllung zu erreichen?
4. Wie frustriert (unsicher, entmutigt, irritiert, gestresst und verärgert) fühlten Sie sich während der Aufgabe?

**Ethics Position Questionnaire (EPQ)**

*Bitte geben Sie den Grad Ihrer Zustimmung zu folgenden Aussagen an.*

1. Das Wohl anderer zu opfern, ist niemals wirklich notwendig.
2. Moralische Standards sollten als etwas Individuelles gesehen werden: Was eine Person als moralisch ansieht, kann eine andere als unmoralisch bewerten.
3. Die Würde und das Wohlergehen der Menschen sollten die wichtigste Sorge in jeder Gesellschaft sein.
4. Ob eine Lüge als unmoralisch oder sogar moralisch zu beurteilen ist, hängt ganz von den Umständen ab.
5. In sozialen Beziehungen sind ethische Probleme oft so komplex, dass man Personen erlauben sollte, ihre eigenen persönlichen Regeln zu finden.
6. Was „ethisch“ ist, variiert zwischen Situationen und Kulturen.
7. Es ist unmoralisch, negative Folgen einer Handlung durch positive Folgen verrechnen zu wollen.
8. Man darf andere Personen weder psychisch noch physisch schädigen.
9. Wenn eine Handlung eine unschuldige Person schädigen könnte, muss man sie unterlassen.
10. Es gibt keine ethischen Prinzipien, die so wichtig sind, dass sie eine allgemeingültige Vorschrift bilden könnten.
11. Moralisches Handeln liegt dann vor, wenn es der Ideal-Handlung entspricht.
12. Man darf keine Handlungen ausführen, die in irgendeiner Weise die Würde und das Wohlergehen anderer Personen bedrohen.
13. Eine starre Ethik-Vorschrift, die bestimmte Handlungsmöglichkeiten verhindern soll, kann der Verbesserung sozialer Beziehungen sogar im Wege stehen.
14. Risiken in Kauf zu nehmen, die andere Personen betreffen, ist nicht tolerierbar, egal wie gering sie sind.
15. Potentielle Schädigungen Dritter in Kauf zu nehmen, ist immer schlecht, egal welche guten Zwecke verfolgt werden.
16. Moralisches Standards sind jeweils persönliche Regeln, sie sollten nicht auf die Beurteilung anderer angewendet werden.
17. Die Frage, was ethisch richtig ist, wird sich niemals beantworten lassen, da es sich bei der Entscheidung, was moralisch oder unmoralisch ist, um eine persönliche Entscheidung handelt.
18. Man sollte sichergehen, mit seinen Handlungen niemanden zu verletzen oder zu schädigen.
19. Verschiedene Arten von Moral dürfen nicht als mehr oder weniger „Gut“ bewertet werden.
20. Über das Lügen lässt sich keine Regel formulieren; ob eine Lüge zulässig ist oder nicht, hängt von der Situation ab.

## Appendix 4

### Information about the AI-based system (translated from German)

#### Important note on the implemented AI technology

The implemented AI makes use of various algorithms. During development, a great focus was placed on fair and ethical decisions. The AI distinguishes between applicants on the basis of a variety of features (characteristics) that have been selected by an independent panel of experts. Access to the applicants' personal information is technically prevented. The measures implemented ensure that the evaluation carried out by the AI is always based on objective factors. The aim is to encourage the decision-maker to make more ethical decisions in candidate selection processes.

## Appendix 5

Fig. 7

### BFI-10 findings

The BFI-10 questionnaire was assessed on a 7-point Likert scale to evaluate the participants’ personalities. To calculate the BFI scores, the negatively formulated items were inverted, and the mean values of the two items of each of the five dimensions were calculated. Afterward, descriptive statistics were generated, as shown in the Table 8 below. The BFI-scores of the groups with and without AI recommendations and the BFI scores of the groups with and without XAI were compared with independent samples t-tests. There were no significant differences between the respective groups.

**Table 8:** Results of BFI-10

Dimension	*M (SD)*_*AI rec*_	*M (SD)*_*No rec*_	*M (SD) *_*XAI*_	*M (SD)*_*No XAI*_	*M (SD)*_*Total*_
Extraversion	4.09 *(1.5)*	4.19 *(1.50)*	4.19 *(1.44)*	4.09 *(1.58)*	4.14 *(1.50)*
Agreeableness	4.27 *(1.3)*	4.38 *(1.16)*	4.23 *(1.29)*	4.42 *(1.17)*	4.33 *(1.23)*
Conscientiousness	4.95 *(1.26)*	5.12 *(1.15)*	5.06 *(1.13)*	5.00 *(1.29)*	5.03 *(1.21)*
Neuroticism	4.18 *(1.38)*	4.22 *(1.3)*	4.12 *(1.33)*	4.27 *(1.35)*	4.20 *(1.34)*
Openness	5.05 *(1.39)*	4.93 *(1.54)*	5.08 *(1.49)*	4.90 *(1.44)*	4.99 *(1.46)*

## Appendix 6

### Participants reasons

https://osf.io/3tjre/?view_only=47915ebaca684083acd568b9a7b4941b

## Footnotes

1 https://bubble.io/

## References

[CR1] Abrams D, Swift HJ, Drury L (2016). Old and unemployable? How age-based stereotypes affect willingness to hire job candidates. Journal of Social Issues.

[CR2] Adadi A, Berrada M (2018). Peeking inside the black-box: A survey on explainable artificial intelligence (XAI). IEEE Access.

[CR3] Akinlade EY, Lambert JR, Zhang P (2020). Mechanisms for hiring discrimination of immigrant applicants in the United States. Equality, Diversity and Inclusion: An International Journal.

[CR4] Ameri M, Schur L, Adya M, Bentley FS, McKay P, Kruse D (2018). The disability employment puzzle: A field experiment on employer hiring behavior. ILR Review.

[CR5] Baert, S. (2018). Hiring discrimination: An overview of (almost) all correspondence experiments since 2005. In *Audit studies: Behind the scenes with theory, method, and nuance* (pp. 63–77). Springer International Publishing. 10.1007/978-3-319-71153-9_3

[CR6] Baert S, Albanese A, du Gardein S, Ovaere J, Stappers J (2017). Does work experience mitigate discrimination?. Economics Letters.

[CR7] Baird A, Maruping LM (2021). The next generation of research on is use: A theoretical framework of delegation to and from agentic is artifacts. MIS Quarterly Management Information Systems.

[CR8] Barocas S, Selbst AD (2016). Big data’s disparate impact. SSRN Electronic Journal.

[CR9] Barredo Arrieta A, Díaz-Rodríguez N, Del Ser J, Bennetot A, Tabik S, Barbado A, Garcia S, Gil-Lopez S, Molina D, Benjamins R, Chatila R, Herrera F (2020). Explainable artificial intelligence (XAI): Concepts, taxonomies, opportunities and challenges toward responsible AI. Information Fusion.

[CR10] Berente N, Gu B, Recker J, Santhanam R (2021). Managing artificial intelligence. MIS Quarterly.

[CR11] Berger B, Adam M, Rühr A, Benlian A (2021). Watch me improve—algorithm aversion and demonstrating the ability to learn. Business and Information Systems Engineering.

[CR12] Bigman YE, Yam KC, Marciano D, Reynolds SJ, Gray K (2021). Threat of racial and economic inequality increases preference for algorithm decision-making. Computers in Human Behavior.

[CR13] Black JS, van Esch P (2020). AI-enabled recruiting: What is it and how should a manager use it?. Business Horizons.

[CR14] Burke G, Mendoza M, Linderman J, Tarm M (2021). How AI-powered tech landed man in jail with scant evidence.

[CR15] Carlsson R, Sinclair S (2018). Prototypes and same-gender bias in perceptions of hiring discrimination. The Journal of Social Psychology.

[CR16] Chong L, Zhang G, Goucher-Lambert K, Kotovsky K, Cagan J (2022). Human confidence in artificial intelligence and in themselves: The evolution and impact of confidence on adoption of AI advice. Computers in Human Behavior.

[CR17] Cole MS, Feild HS, Giles WF (2004). Interaction of recruiter and applicant gender in resume evaluation: A field study. Sex Roles.

[CR18] Correll SJ, Benard S, Paik I (2007). Getting a job: Is there a motherhood penalty?. American Journal of Sociology.

[CR19] Dastin, J. (2018). *Amazon scraps secret AI recruiting tool that showed bias against women*. Reuters. https://www.reuters.com/article/us-amazon-com-jobs-automation-insight-idUSKCN1MK08G

[CR20] Davidson S (2016). Gender inequality: Nonbinary transgender people in the workplace. Cogent Social Sciences.

[CR21] Davidson R, MacKinnon J (1993). Estimation and inference in econometrics.

[CR22] Dellermann D, Ebel P, Söllner M, Leimeister JM (2019). Hybrid intelligence. Business and Information Systems Engineering.

[CR23] Dietvorst BJ, Simmons JP, Massey C (2015). Algorithm aversion: People erroneously avoid algorithms after seeing them err. Journal of Experimental Psychology: General.

[CR24] Dikmen M, Burns C (2022). The effects of domain knowledge on trust in explainable AI and task performance: A case of peer-to-peer lending. International Journal of Human Computer Studies.

[CR25] Dwivedi, Y. K., Hughes, L., Ismagilova, E., Aarts, G., Coombs, C., Crick, T., Duan, Y., Dwivedi, R., Edwards, J., Eirug, A., Galanos, V., Ilavarasan, P. V., Janssen, M., Jones, P., Kar, A. K., Kizgin, H., Kronemann, B., Lal, B., Lucini, B., Medaglia, R., Le Meunier-FitzHugh, K., Le Meunier-FitzHugh, L. C., Misra, S., Mogaji, E., Kumar Sharma, S., Bahadur Singh, J., Raghavan, V., Raman, R., Rana, N. P., Samothrakis, S., Spencer, J., Tamilmani, K., Tubadji A., Waltony, P., & Williams, M. D. (2021). Artificial Intelligence (AI): Multidisciplinary perspectives on emerging challenges, opportunities, and agenda for research, practice and policy. *International Journal of Information Management*, *57*, 101994. 10.1016/j.ijinfomgt.2019.08.002

[CR26] Ebel P, Söllner M, Leimeister JM, Crowston K, de Vreede G-J (2021). Hybrid intelligence in business networks. Electronic Markets.

[CR27] Feloni, R. (2017). *Consumer goods giant Unilever has been hiring employees using brain games and artificial intelligence — and it’s a huge success*. Business Insider Australia. https://www.businessinsider.in/Consumer-goods-giant-Unilever-has-been-hiring-employees-using-brain-games-and-artificial-intelligence-and-its-a-huge-success/articleshow/59356757.cms

[CR28] Fernández-Martínez C, Fernández A (2020). AI and recruiting software: Ethical and legal implications. Paladyn, Journal of Behavioral Robotics.

[CR29] Fiske ST, Bersoff DN, Borgida E, Deaux K, Heilman M (1991). Social science research on trial: Use of sex stereotyping research in Price Waterhouse v. Hopkins. American Psychologist.

[CR30] Foley M, Williamson S (2018). Does anonymising job applications reduce gender bias?. Gender in Management: An International Journal.

[CR31] Foschi M, Lai L, Sigerson K (1994). Gender and double standards in the assessment of job applicants. Social Psychology Quarterly.

[CR32] Franke, T., Attig, C., & Wessel, D. (2017). *Assessing affinity for technology interaction – the affinity for technology assessing affinity for technology interaction ( ATI )*. *July*. 10.13140/RG.2.2.28679.50081

[CR33] Funder DC, Ozer DJ (2019). Evaluating effect size in psychological research: Sense and nonsense. Advances in Methods and Practices in Psychological Science.

[CR34] Gilpin, L. H., Bau, D., Yuan, B. Z., Bajwa, A., Specter, M., & Kagal, L. (2018). Explaining explanations: An overview of interpretability of machine learning. *2018 IEEE 5th International Conference on Data Science and Advanced Analytics (DSAA)* (pp. 80–89). 10.1109/DSAA.2018.00018

[CR35] González MJ, Cortina C, Rodríguez J (2019). The role of gender stereotypes in hiring: A field experiment. European Sociological Review.

[CR36] Gorges H (2015). HR braucht mehr Männer.

[CR37] Guidotti R, Monreale A, Ruggieri S, Turini F, Giannotti F, Pedreschi D (2018). A survey of methods for explaining black box models. ACM Computing Surveys.

[CR38] Gulati SN, Sousa SC, Lamas D (2019). Design, development and evaluation of a human-computer trust scale. Behaviour and Information Technology.

[CR39] Gunning D, Stefik M, Choi J, Miller T, Stumpf S, Yang G-Z (2019). XAI—Explainable artificial intelligence. Science Robotics.

[CR40] Guryan J, Charles KK (2013). Taste-based or statistical discrimination: The economics of discrimination returns to its roots. The Economic Journal.

[CR41] Hart SG, Staveland LE (1988). Development of NASA-TLX (task load index): Results of empirical and theoretical research. Power Technology and Engineering.

[CR42] Hayes, A. F. (2018). *Introduction to mediation, moderation, and conditional process analysis: A regression-based perspective* (2nd ed.). Guilford Press.

[CR43] Hepenstal, S., & McNeish, D. (2020). Explainable artificial intelligence: What do you need to know? In *Lecture notes in computer science (including subseries lecture notes in artificial intelligence and lecture notes in bioinformatics): Vol. 12196 LNAI* (Issue Lipton 2016). Springer International Publishing. 10.1007/978-3-030-50353-6_20

[CR44] Hofeditz, L., Mirbabaie, Mi., Stieglitz, S., & Holstein, J. (2021). Do you trust an AI-Journalist? A credibility analysis of news content with AI-Authorship. *Proceedings of the 28th European Conference on Information Systems*. Marakech, Morocco.

[CR45] Hofeditz, L., Harbring, M., Mirbabaie, M., & Stieglitz, S. (2022a). Working with ELSA – how an emotional support agent builds trust in virtual teams. *Hawaii International Conference on System Sciences (HICSS)*, Maui, Hawaii.

[CR46] Hofeditz, L., Mirbabaie, M., Luther, A., Mauth, R., & Rentemeister, I. (2022b). Ethics guidelines for using ai-based algorithms in recruiting: Learnings from a systematic literature review. *Hawaii International Conference on System Sciences (HICSS)*, Maui, Hawaii.

[CR47] Hoffman, R. R., Mueller, S. T., Klein, G., & Litman, J. (2018). *Metrics for explainable AI: Challenges and prospects* (pp. 1–50). 10.48550/arXiv.1812.04608

[CR48] Houser KA (2019). Can AI solve the diversity problem in the tech industry? Mitigating noise and bias in employment decision-making. Stanford Technology Law Review.

[CR49] Hu, J. (2019). *99% of Fortune 500 Companies use Applicant Tracking Systems*. Jobscan. https://www.jobscan.co/blog/99-percent-fortune-500-ats/

[CR50] Hussain, F., Hussain, R., & Hossain, E. (2021). Explainable Artificial Intelligence (XAI): An engineering perspective. *arXiv*, 1–11. 10.48550/arXiv.2101.03613

[CR51] John, O. P., & Srivastava, S. (1999). The big five trait taxonomy: History, measurement, and theoretical perspectives. In *Handbook of personality: Theory and research, 2nd ed.* (pp. 102–138). Guilford Press.

[CR52] Jussupow, E., Benbasat, I., & Heinzl, A. (2020). Why are we averse towards algorithms? A comprehensive literature review on algorithm aversion. *Proceedings of the 28th European Conference on Information Systems. *Marakech, Morocco.

[CR53] Köchling A, Riazy S, Wehner MC, Simbeck K (2021). Highly accurate, but still discriminatory: A fairness evaluation of algorithmic video analysis in the recruitment context. Business and Information Systems Engineering.

[CR54] Krause A, Rinne U, Zimmermann KF (2012). Anonymous job applications in Europe. IZA Journal of European Labor Studies.

[CR55] Kübler D, Schmid J, Stüber R (2018). Gender discrimination in hiring across occupations: A nationally-representative vignette study. Labour Economics.

[CR56] Kulshrestha J, Eslami M, Messias J, Zafar MB, Ghosh S, Gummadi KP, Karahalios K (2019). Search bias quantification: Investigating political bias in social media and web search. Information Retrieval Journal.

[CR57] Kuncel NR, Klieger DM, Ones DS (2014). In hiring, algorithms beat instinct. Harvard Business Review.

[CR58] Lancee B (2021). Ethnic discrimination in hiring: comparing groups across contexts. Results from a cross-national field experiment. Journal of Ethnic and Migration Studies.

[CR59] Laurim, V., Arpaci, S., Prommegger, B., & Krcmar, H. (2021). Computer, whom should I hire? - Acceptance criteria for artificial intelligence in the recruitment process. *Proceedings of the Annual Hawaii International Conference on System Sciences*, *2020*-*Janua* (pp. 5495–5504). 10.24251/hicss.2021.668

[CR60] Lepak DP, Marrone JA, Takeuchi R (2004). The relativity of HR systems: Conceptualising the impact of desired employee contributions and HR philosophy. International Journal of Technology Management.

[CR61] Lepri B, Oliver N, Letouzé E, Pentland A, Vinck P (2018). Fair, transparent, and accountable algorithmic decision-making processes. Philosophy & Technology.

[CR62] Li, L., Lassiter, T., Oh, J., & Lee, M. K. (2021). Algorithmic hiring in practice: Recruiter and HR professional’s perspectives on AI use in hiring. *Proceedings of the 2021 AAAI/ACM Conference on AI, Ethics, and Society*, *1*(1), 166–176. 10.1145/3461702.3462531

[CR63] Liao, Q. V., Gruen, D., & Miller, S. (2020). Questioning the AI: informing design practices for explainable AI user experiences. *Proceedings of the 2020 CHI Conference on Human Factors in Computing Systems* (pp. 1–15). 10.1145/3313831.3376590

[CR64] Lössbroek J, Lancee B, van der Lippe T, Schippers J (2021). Age discrimination in hiring decisions: A factorial survey among managers in nine European countries. European Sociological Review.

[CR65] Mayring, P. (1994). *Qualitative Inhaltsanalyse*. http://nbn-resolving.de/urn:nbn:de:0168-ssoar-14565

[CR66] Mehrotra, A., & Celis, L. E. (2021). Mitigating bias in set selection with noisy protected attributes. *FAccT 2021 - Proceedings of the 2021 ACM Conference on Fairness, Accountability, and Transparency* (pp. 237–248). 10.1145/3442188.3445887

[CR67] Meske C, Bunde E, Degen H, Reinerman-Jones L (2020). Transparency and trust in human-AI-interaction: The role of model-agnostic explanations in computer vision-based decision support. Artificial intelligence in HCI.

[CR68] Meske C, Bunde E, Schneider J, Gersch M (2022). Explainable artificial intelligence: Objectives, stakeholders, and future research opportunities. Information Systems Management.

[CR69] Mingers J, Walsham G (2010). Toward ethical information systems: The contribution of discourse ethics. MIS Quarterly.

[CR70] Mirbabaie M, Brünker F, Möllmann (Frick) NRJ, Stieglitz S (2021). The rise of artificial intelligence – understanding the AI identity threat at the workplace. Electronic Markets.

[CR71] Mirbabaie M, Stieglitz S, Brünker F, Hofeditz L, Ross B, Frick NRJ (2021). Understanding collaboration with virtual assistants – the role of social identity and the extended self. Business and Information Systems Engineering.

[CR72] Mirbabaie M, Stieglitz S, Frick NRJ (2021). Hybrid intelligence in hospitals: Towards a research agenda for collaboration. Electronic Markets.

[CR73] Mittelstadt, B., Russell, C., & Wachter, S. (2019). Explaining explanations in AI. *Proceedings of the Conference on Fairness, Accountability, and Transparency* (pp. 279–288). 10.1145/3287560.3287574

[CR74] Mujtaba, D. F., & Mahapatra, N. R. (2019). Ethical considerations in AI-based recruitment. *2019 IEEE International Symposium on Technology and Society (ISTAS)* (pp. 1–7). 10.1109/ISTAS48451.2019.8937920

[CR75] Neumark D (2018). Experimental research on labor market discrimination. Journal of Economic Literature.

[CR76] Neumark, D. (2021). Age discrimination in hiring: Evidence from age-blind vs. non-age-blind hiring procedures. *Journal of Human Resources*, *August*, 0420-10831R1. 10.3368/jhr.0420-10831R1

[CR77] Neumark D, Burn I, Button P (2017). Age discrimination and hiring of older workers. FRBSF Economic Letter.

[CR78] Ochmann, J., & Laumer, S. (2019). Fairness as a determinant of AI adoption in recruiting: An interview-based study. *DIGIT 2019 Proceedings*. https://aisel.aisnet.org/digit2019/16

[CR79] Ochmann, J., Zilker, S., Michels, L., Tiefenbeck, V., & Laumer, S. (2021). The influence of algorithm aversion and anthropomorphic agent design on the acceptance of AI-based job recommendations. *International Conference on Information Systems, ICIS 2020* (pp. 17).

[CR80] Oppenheimer DM, Meyvis T, Davidenko N (2009). Instructional manipulation checks: Detecting satisficing to increase statistical power. Journal of Experimental Social Psychology.

[CR81] Pan, Y., Froese, F., Liu, N., Hu, Y., & Ye, M. (2021). The adoption of artificial intelligence in employee recruitment: The influence of contextual factors. *The International Journal of Human Resource Management*, 1–23. 10.1080/09585192.2021.1879206

[CR82] Petersen T, Saporta I (2004). The opportunity structure for discrimination. American Journal of Sociology.

[CR83] Petersen T, Togstad T (2006). Getting the offer: Sex discrimination in hiring. Research in Social Stratification and Mobility.

[CR84] Quillian L, Pager D, Hexel O, Midtbøen AH (2017). Meta-analysis of field experiments shows no change in racial discrimination in hiring over time. Proceedings of the National Academy of Sciences of the United States of America.

[CR85] Quillian L, Heath A, Pager D, Midtbøen A, Fleischmann F, Hexel O (2019). Do some countries discriminate more than others? Evidence from 97 field experiments of racial discrimination in hiring. Sociological Science.

[CR86] Raghavan, M., Barocas, S., Kleinberg, J., & Levy, K. (2020). Mitigating bias in algorithmic hiring: Evaluating claims and practices. *FAT* 2020 - Proceedings of the 2020 Conference on Fairness, Accountability, and Transparency* (pp. 469–481). 10.1145/3351095.3372828

[CR87] Raisch S, Krakowski S (2021). Artificial intelligence and management: The automation–augmentation paradox. Academy of Management Review.

[CR88] Rammstedt, B., Kemper, C., Klein, M., Beierlein, C., & Kovaleva, A. (2013). Eine kurze Skala zur Messung der fünf Dimensionen der Persönlichkeit: 10 Item Big Five Inventory (BFI-10). *Methoden, Daten, Analysen (Mda)*, *7*(2), 233–249. 10.12758/mda.2013.013

[CR89] Richardson, B., Webb, J., Webber, L., & Smith, K. (2013). *Age discrimination in the evaluation of job applicants: Discovery Service for University of Portsmouth* (pp. 35–44). 10.1111/j.1559-1816.2013.00979.x

[CR90] Rieskamp, J., Hofeditz, L., Mirbabaie, M., & Stieglitz, S. (2023). Approaches to improve fairness when deploying ai-based algorithms in hiring – using a systematic literature review to guide future research. *Hawaii International Conference on System Sciences*. Maui, Hawaii.

[CR91] Rouse WB (2020). AI as systems engineering augmented intelligence for systems engineers. Insight.

[CR92] Ruffle BJ, Shtudiner Z (2015). Are good-looking people more employable?. Management Science.

[CR93] Russell, S. J., & Norvig, P. (2016). *Artificial Intelligence: A Modern Approach*. Pearson Education Limited.

[CR94] Sabeg Y, Me´haignerie L (2006). Les oublie´s de l’e´galite´ des chances [The forgotten ones of the equality of opportunity].

[CR95] Sánchez-Monedero, J., Dencik, L., & Edwards, L. (2020). What does it mean to “solve” the problem of discrimination in hiring? *Proceedings of the 2020 Conference on Fairness, Accountability, and Transparency* (pp. 458–468). 10.1145/3351095.3372849

[CR96] Schmidt P, Biessmann F, Teubner T (2020). Transparency and trust in artificial intelligence systems. Journal of Decision Systems.

[CR97] Schoonderwoerd Tjeerd A.J., Zoelen Emma M. van, Bosch Karel van den, Neerincx Mark A. (2022). Design patterns for human-AI co-learning: A wizard-of-Oz evaluation in an urban-search-and-rescue task. International Journal of Human-Computer Studies.

[CR98] Shin D (2021). The effects of explainability and causability on perception, trust, and acceptance: Implications for explainable AI. International Journal of Human-Computer Studies.

[CR99] Sokol, K., & Flach, P. (2020). Explainability fact sheets. *Proceedings of the 2020 Conference on Fairness, Accountability, and Transparency* (pp. 56–67). 10.1145/3351095.3372870

[CR100] Stone A, Wright T (2013). When your face doesn’t fit: Employment discrimination against people with facial disfigurements. Journal of Applied Social Psychology.

[CR101] Strack, M., & Gennerich, C. (2007). Erfahrung mit Forsyths ’Ethic Position Questionnaire? (EPQ): Bedeutungsunabhängigkeit von Idealismus und Realismus oder Akquieszens und Biplorarität? *Berichte Aus Der Arbeitsgruppe “Verantwortung, Gerechtigkeit, Moral”, Nr. 167, ISSN 1430-1148*.

[CR102] Sühr, T., Hilgard, S., & Lakkaraju, H. (2021). Does fair ranking improve minority outcomes? Understanding the interplay of human and algorithmic biases in online hiring. *AIES 2021 - Proceedings of the 2021 AAAI/ACM Conference on AI, Ethics, and Society* (pp. 989–999). 10.1145/3461702.3462602

[CR103] Teodorescu MHM, Morse L, Awwad Y, Kane GC (2021). Failures of fairness in automation require a deeper understanding of human–ml augmentation. MIS Quarterly: Management Information Systems.

[CR104] Thiebes S, Lins S, Sunyaev A (2020). Trustworthy artificial intelligence. Electronic Markets.

[CR105] Tosi HL, Einbender SW (1985). The effects of the type and amount of information in sex discrimination research: A meta-analysis. Academy of Management Journal.

[CR106] van Giffen B, Herhausen D, Fahse T (2022). Overcoming the pitfalls and perils of algorithms: A classification of machine learning biases and mitigation methods. Journal of Business Research.

[CR107] Weichselbaumer, D. (2016). Discrimination against female migrants wearing headscarves. *SSRN Electronic Journal*, *10217*. 10.2139/ssrn.2842960

[CR108] Weiss, A., Bernhaupt, R., Schwaiger, D., Altmaninger, M., Buchner, R., & Tscheligi, M. (2009). User experience evaluation with a Wizard of Oz approach: Technical and methodological considerations. *9th IEEE-RAS International Conference on Humanoid Robots*, (pp. 303–308). 10.1109/ICHR.2009.5379559

[CR109] Wijnhoven, F. (2021). Organizational learning for intelligence amplification adoption: Lessons from a clinical decision support system adoption project. *Information Systems Frontiers*, *0123456789*.10.1007/s10796-021-10206-9

[CR110] Wijnhoven F, van Haren J (2021). Search engine gender bias. Frontiers in Big Data.

[CR111] Wilson, J., & Rosenberg, D. (1988). Rapid prototyping for user interface design. In *Handbook of human-computer interaction*. Elsevier B.V. 10.1016/b978-0-444-70536-5.50044-0

[CR112] Zaniboni S, Kmicinska M, Truxillo DM, Kahn K, Paladino MP, Fraccaroli F (2019). Will you still hire me when I am over 50? The effects of implicit and explicit age stereotyping on resume evaluations. European Journal of Work and Organizational Psychology.

[CR113] Zhu, J., Liapis, A., Risi, S., Bidarra, R., & Youngblood, G. M. (2018). Explainable AI for designers: A human-centered perspective on mixed-initiative co-creation. *2018 IEEE Conference on Computational Intelligence and Games (CIG)*, *2018*-*Augus* (pp. 1–8). 10.1109/CIG.2018.8490433

[CR114] Zschirnt E, Ruedin D (2016). Ethnic discrimination in hiring decisions: A meta-analysis of correspondence tests 1990–2015. Journal of Ethnic and Migration Studies.

