# Supplementary material for: Applying XAI to an AI-based system for candidate management to mitigate bias and discrimination in hiring
Source: Electron Mark. 2022 Dec 20;32(4):2207–33. doi: 10.1007/s12525-022-00600-9 (PMC9764302; doi:10.1007/s12525-022-00600-9)
Supplement: Supplementary file 1 — Supplementary file1 (PDF 483 KB) [file 12525_2022_600_MOESM1_ESM.pdf]

Appendix

Appendix A Candidate Profiles

Job	Round	Type	Prename	Name	Gender	Age	Birthplace	Nationality	Lang.	Deg.	Exp.	Social	Extra
1	1	0	Dirk	Bauer	Male	41	Solingen	German	3	4	3	3	2
1	1	1	Mustafa	Özdemir	Male	43	Tarsus	Turkish	2	3	4	3	3
1	2	1	Ecrin	Şahin	Female	37	Adana	Turkish	4	3	1	2	4
1	2	0	Stephanie	Jung	Female	39	Kerpen	German	3	2	2	4	3
1	3	99	Paulina	Krasny	Female	37	Breslau	Polish	1	3	2	2	4
1	3	98	Wolfgang	Fischer	Male	54	Wittlich	German	3	2	5	1	1
1	4	99	Mathias	Eisenberg	Male	42	Ulm	German	1	2	3	1	3
1	4	98	Dennis	Zimmer	Male	37	Rosenheim	German	3	4	2	3	2
1	5	1	Miray	Korkmaz	Female	34	Istanbul	Turkish	1	3	2	4	4
1	5	0	Anke	Faber	Female	33	Minden	German	2	2	3	4	3
1	6	99	Maria	van Dijk	Female	39	Venlo	Dutch	4	2	4	3	3
1	6	98	Jürgen	Fuhrmann	Male	51	Bramsche	German	2	3	3	5	2
2	1	99	Luisa	Engel	Female	56	Marburg	German	3	4	4	2	2
2	1	98	Sophia	Thalberg	Female	47	Lörrach	German	2	3	3	4	5
2	2	98	Oskar	Borkowski	Male	37	Lissa	Polish	2	3	3	1	5
2	2	99	Simone	Wulf	Female	33	Leipzig	German	5	5	1	4	3
2	3	0	Sven	Kuster	Male	33	Flensburg	German	2	4	3	2	4
2	3	1	Thomas	Ackermann	Male	54	Tübingen	German	2	2	4	4	3
2	4	1	Monika	Zimmer	Female	57	Niebüll	German	3	4	4	2	3
2	4	0	Lisa	Schaefer	Female	36	Babelsberg	German	2	5	3	4	2
2	5	98	Philipp	Neumann	Male	54	Essen	German	2	3	4	5	4
2	5	99	Katja	Weiß	Female	36	Halle (Saale)	German	4	2	3	5	2

2	6	0	Martin	Bach	Male	39	Nürnberg	German	5	3	2	4	1
2	6	1	Patrick	Lehmann	Male	51	Hilden	German	3	3	3	2	4
3	1	99	Robin	Winkler	Male	51	Wesel	German	3	5	3	2	5
3	1	98	Jannik	Grunewald	Male	48	Bocholt	German	3	2	4	3	4
3	2	0	Christian	Nacht	Male	37	Bayreuth	German	2	3	3	5	2
3	2	1	Katharina	Decker	Female	36	Celle	German	3	4	2	3	3
3	3	99	Laura	Fischer	Female	34	Paderborn	German	2	1	2	3	2
3	3	98	Aylin	Öztürk	Female	45	Mersin	Turkish	4	2	4	2	3
3	4	0	Arne	Meyer	Male	44	Halberstadt	German	4	4	4	2	3
3	4	1	Karin	Richter	Female	42	Fulda	German	3	5	4	3	2
3	5	1	Sophia	Ostermann	Female	34	Dresden	German	2	3	2	4	2
3	5	0	Dominik	Braun	Male	33	Rathenow	German	3	4	1	2	3
3	6	98	Anna	Iwanow	Female	57	Krasnojarsk	Russian	3	4	5	3	2
3	6	99	Aaron	Becker	Male	39	Koblenz	German	2	3	3	4	5

Note. Column Type: 0 = relevant; 1 = relevant with recommendation; 98 = non-relevant with recommendation; 99 = non-relevant. Lang. = Languages; Deg. = Degree; Exp. = Work Experience; Social = Social Skills; Extra = Dynamic Field.

Appendix B Questionnaires

All questionnaires are based on a 7-point Likert scale.

Big Five Inventory (BFI-10)

Bitte geben Sie den Grad Ihrer Zustimmung zu folgenden Aussagen an.

1. Ich bin eher zurückhaltend, reserviert.
2. Ich schenke anderen leicht Vertrauen, glaube an das Gute im Menschen.
3. Ich bin bequem, neige zur Faulheit.
4. Ich bin entspannt, lasse mich durch Stress nicht aus der Ruhe bringen.
5. Ich habe nur wenig künstlerisches Interesse.
6. Ich gehe aus mir heraus, bin gesellig.
7. Ich neige dazu, andere zu kritisieren.
8. Ich erledige Aufgaben gründlich.
9. Ich werde leicht nervös und unsicher.
10. Ich habe eine aktive Vorstellungskraft, bin fantasievoll.

Affinity for Technology Interaction (ATI)

Bitte geben Sie den Grad Ihrer Zustimmung zu folgenden Aussagen an.

Mit „technischen Systemen" sind sowohl Apps und andere Software-Anwendungen als auch komplette digitale Geräte (z.B. Handy, Computer, Fernseher, Auto-Navigation) gemeint.

1. Ich beschäftige mich gern genauer mit technischen Systemen.
2. Ich probiere gern die Funktionen neuer technischer Systeme aus.
3. In erster Linie beschäftige ich mich mit technischen Systemen, weil ich muss.
4. Wenn ich ein neues technisches System vor mir habe, probiere ich es intensiv aus.
5. Ich verbringe sehr gern Zeit mit dem Kennenlernen eines neuen technischen Systems.
6. Es genügt mir, dass ein technisches System funktioniert, mir ist es egal, wie oder warum.
7. Ich versuche zu verstehen, wie ein technisches System genau funktioniert.
8. Es genügt mir, die Grundfunktionen eines technischen Systems zu kennen.
9. Ich versuche, die Möglichkeiten eines technischen Systems vollständig auszunutzen.

Human Computer Trust Scale (HCTS)

Bitte geben Sie den Grad Ihrer Zustimmung zu folgenden Aussagen an.

Eine künstliche Intelligenz (KI) lässt sich als System beschreiben, dass die Fähigkeit besitzt, sich selbstständig an neue Situationen und Inhalte anzupassen. Es kann Probleme lösen und Aufgaben erledigen, die ein gewisses Maß an Intelligenz erfordern, wie sie typischerweise bei Menschen vorhanden ist.

1. Ich glaube, dass der Einsatz einer künstlichen Intelligenz negative Folgen haben könnte.
2. Ich glaube, ich muss vorsichtig sein, wenn ich eine künstliche Intelligenz verwende.
3. Es ist riskant, mit einer künstlichen Intelligenz zu interagieren.
4. Ich glaube, dass eine künstliche Intelligenz in meinem besten Interesse handeln wird.
5. Ich glaube, dass eine künstliche Intelligenz ihr Bestes tun wird, um mir zu helfen, wenn ich Hilfe benötige.

6. Ich glaube, dass eine künstliche Intelligenz daran interessiert ist, meine Bedürfnisse und Vorlieben zu verstehen.
7. Ich denke, dass eine künstliche Intelligenz bei der Auswahl von Bewerber:innen kompetent und effektiv ist.
8. Ich denke, dass eine künstliche Intelligenz ihre Rolle als Instrument zur Bewerberauswahl sehr gut erfüllt.
9. Ich glaube, dass eine künstliche Intelligenz über alle Funktionen verfügt, die ich von einem Hilfsmittel zur Bewerberauswahl erwarten würde.
10. Wenn ich eine künstliche Intelligenz verwende, denke ich, dass ich mich vollständig auf sie verlassen kann.
11. Ich kann mich immer auf eine künstliche Intelligenz verlassen, wenn es um die Entscheidungsfindung geht.
12. Ich kann den Informationen vertrauen, die mir eine künstliche Intelligenz liefert.

NASA Task Load Index (NASA-TLX)

1. Wie viel geistige Anforderung war bei der Aufnahme und Verarbeitung von Informationen erforderlich? War die Aufgabe einfach oder komplex?
2. Wie erfolgreich haben Sie Ihrer Meinung nach die vom Versuchsleiter (oder Ihnen selbst) gesetzten Ziele erreicht?
3. Wie anstrengend war die Arbeit, um Ihren Grad an Aufgabenerfüllung zu erreichen?
4. Wie frustriert (unsicher, entmutigt, irritiert, gestresst und verärgert) fühlten Sie sich während der Aufgabe?

Ethics Position Questionnaire (EPQ)

Bitte geben Sie den Grad Ihrer Zustimmung zu folgenden Aussagen an.

1. Das Wohl anderer zu opfern, ist niemals wirklich notwendig.
2. Moralische Standards sollten als etwas Individuelles gesehen werden: Was eine Person als moralisch ansieht, kann eine andere als unmoralisch bewerten.
3. Die Würde und das Wohlergehen der Menschen sollten die wichtigste Sorge in jeder Gesellschaft sein.
4. Ob eine Lüge als unmoralisch oder sogar moralisch zu beurteilen ist, hängt ganz von den Umständen ab.
5. In sozialen Beziehungen sind ethische Probleme oft so komplex, dass man Personen erlauben sollte, ihre eigenen persönlichen Regeln zu finden.
6. Was „ethisch“ ist, variiert zwischen Situationen und Kulturen.
7. Es ist unmoralisch, negative Folgen einer Handlung durch positive Folgen verrechnen zu wollen.
8. Man darf andere Personen weder psychisch noch physisch schädigen.
9. Wenn eine Handlung eine unschuldige Person schädigen könnte, muss man sie unterlassen.
10. Es gibt keine ethischen Prinzipien, die so wichtig sind, dass sie eine allgemeingültige Vorschrift bilden könnten.
11. Moralisches Handeln liegt dann vor, wenn es der Ideal-Handlung entspricht.
12. Man darf keine Handlungen ausführen, die in irgendeiner Weise die Würde und das Wohlergehen anderer Personen bedrohen.
13. Eine starre Ethik-Vorschrift, die bestimmte Handlungsmöglichkeiten verhindern soll, kann der Verbesserung sozialer Beziehungen sogar im Wege stehen.

14. Risiken in Kauf zu nehmen, die andere Personen betreffen, ist nicht tolerierbar, egal wie gering sie sind.
15. Potentielle Schädigungen Dritter in Kauf zu nehmen, ist immer schlecht, egal welche guten Zwecke verfolgt werden.
16. Moralisches Standards sind jeweils persönliche Regeln, sie sollten nicht auf die Beurteilung anderer angewendet werden.
17. Die Frage, was ethisch richtig ist, wird sich niemals beantworten lassen, da es sich bei der Entscheidung, was moralisch oder unmoralisch ist, um eine persönliche Entscheidung handelt.
18. Man sollte sichergehen, mit seinen Handlungen niemanden zu verletzen oder zu schädigen.
19. Verschiedene Arten von Moral dürfen nicht als mehr oder weniger „Gut“ bewertet werden.
20. Über das Lügen lässt sich keine Regel formulieren; ob eine Lüge zulässig ist oder nicht, hängt von der Situation ab.

Appendix C Participants Reasons

The categories are coded in column C as follows:

- q = Qualification
- k = Recommendation
- r = Race
- a = Age
- e = Ethical
- o = Subjective
- x = Excluded

The tables are sorted according to the conditions.

Table C1 Candidate Selection Reasons of Participants in Condition 1

ID	Reason 1	C	Reason 2	C	Reason 3	C
			**Condition 1 – Job 1**			
1335	bessere Berufserfahrung	q	bessere Softskills	o	bessere Programmierungerfahrung	q
1352	mehr Erfahrung	q	wichtigere Skills	o	bessere qsverteilung	q
1363	Sprachen	q	Berufserfahrung macht sie mit Programmierer-fahrung wett	q	Berufserfahrung macht sie mit Programmierer-fahrung wett	q
1387	programmiererfahrung	q	programmeirefahrung	q	programmieren	q
1402	Bessere Berufserfahrung und Programmi-ererfahrung	q	Bessere soziale Skills	o	Programmiererfahrung und guter Abschluss	q
1415	Berufserfahrung & Programmierung sind wichtig	q	Soziale Skills stehen hier über den niedrigen Erfahrungen des Mitbewerbers	q	In allen Bereichen gut	q
1433	alle Fähigkeiten gut ausgeglichen, hat mehr Berufserfahrung	q	Hat gute Programmiererfahrung	q	Erfahrung und Skills	q
1439	Berufserfahrung und Programmiererfahrung sind Vorteil, da höher bewertet als bei anderem Bewerber.	q	Die höhere Berufserfahrung erscheint hier in Kombination mit der geringeren Programmier-erfahrung als geeigneter.	q	Mehr Berufserfahrung erscheint als Vorteil.	q
1452	Hat bessere Programmiererfahrung	q	Bessere Social Skills	q	Bessere Programmiererfahrung	q
1473	diversität ist auch wichtig	e	mehr Erfahrung	q	mehr programmiererfahrung	q
1498	Berufserfahrung und skills, sonst sind beide ähnlich gut	q	Skills ähnlich, aber der 2. Kandidat kann sich wahrscheinlich besser ins Team integrieren	q, o	Beide Kandidaten sind ähnlich gut, jedoch hat die 2. Kandidatin mehr überzeugt	q, o
1501	Mehr Erfahrung die für Beruf relevant sind	q	mehr Kompetenz die für Beruf relevant sind	q	Bessere Kompetenz	q

1514	Programmiererfahrung und Berufserfahrung	q	Programmiererfahrung	q	Bessere Fähigkeiten	o
1524	Dem anderen Bewerber in Sachen Methodenkompetenz überlegen	q	Mehr Berufserfahrung	q	bessere Programmierkenntnis und besseren Abschluss als die Konkurrentin	q
1532	Da er mehr Beruf und Programiererfahrung als der andere Kandiat hat.	q	SIe hat merh Programmiererfahrung ea für den Job wichtiger ist	q	Soziale Skills,Programmier erfahrung besser	q
1535	Die Person weiß wie man Programiert. Beim Informatikstudium lernt man das nicht.	o	IT Skills sind am Wichtigsten	q	das ist die beste Mischung	o
1550	Bessere Programmierferfahrung	q	Soziale Skills nicht ganz so wichtig, da keine Führungsposition, Programmiererfahrung wichtiger.	q	Mehr Programmiererfahrung	q
1551	Abschluss und Erfahrung	q	Abschluss und Erfahrung	q	Allrounder	o
1567	Der Bewerber verfügt im Vergleich zum Mitbewerber über eine höhere Programmier- und Berufserfahrung. Dies erscheint mir wichtiger als die Sprachkenntnisse und der Abschluss.	q	Die Programmiererfahrung scheint mir bei dem Job die entscheidende q zu sein und diese ist bei diesem Bewerber höher ausgeprägt.	q	Bis auf die Sprachkenntnisse scheint die Bewerberin über gute qen zu verfügen. Ein Sprachkurs könnte Bedingung für die Einstellung sein.	q
1580	mehr Berufserfahrung	q	mehr Berufserfahrung und soziale skills	q	mehr berufserfahrung	q
1589	Programmiererfahrung	q	Programmiererfahrung	q	Programmiererfahrung und skills als ITler	q
1597	Programmiererfahrung sowie Berufserfahrung definitiv relevanter als der Abschluss	q	Programmiererfahrung sowie mehr Berufserfahrung ist hier ausschlaggebend	q	Berufserfahrung ist hier der entscheidende Grund für Bewerber Faber	q
1623	Insgesamt war die Anzahl der Sterne über alle kategorien hinweg für die zwei Bewerber gleich. Allerdings hat Mustafa Özdemir im Hinblick auf Programmiererfahrung und Berufserfahrung besser abgeschnitten (Ich glaube das sind die relevanteren Fähigkeiten/Erfahrungen für diesen Beruf). Ich glaube nicht, dass es für diesen beruf wichtig ist, verschiedene Sprachen zu sprechen. Auch ist der theoretische Abschluss auch nicht unbedungt aussagekräftig für eine solche sehr praktische Tätigkeit	q	Die zuvor genannten wichtigen Aspekte (Programmiererfahrung und Berufserfahrung) sind unter den Bwerberinnen ausgeglichen - auch die Gesamtzahl der Sterne ist identisch. Die in meinen Augen nachfolgend wichtige Eigenschaft ist soziale Skills, da anwenderspezifische Fragestellungen der Belegschaft beantwortet werden müssen.	q	Gesamtzahl der Sterne identisch - ausgeglichen im Hinblick auf Programmiererfahrung, soziale Skills und Berufserfarhung. Abschluss in meinen Augen wichtiger als Sprachen	q
1625	Programmiererfahrung und Berufserfahrung wichtig	q	Soziale Skills wichtig bei Fragen, und Berufserfahrung ist auch wichtig	q	sehr gute Werte auf den wichtigsten, soziale skills und Programmiererfahrung	q
1639	Mehr Berufs- sowie Programmierungserfahrungen vorhanden	q	Hohe Programmierungserfahrung	q	Bessere Programmierungserfahrung	q
1643	Berfuserfahrung höher	q	Bessere Programmiererfahrung	q	Intuition	o
1651	höhere Berufserfahrung & Programmiererfahrung	q	mehr berufserfahrung	q	Sprachen besser	q
1658	Trotz schlechterer Sprache, längere Erfahrungen in der Branche und bessere Kenntnisse!	q	Bessere wichtige Qualitäten, wie soziale Fähigkeiten und langwierige Erfahrung!	q	Längere Erfahrung, ausgeglichene Punktverteilung!	q

1673	Technisches Know-How aus der Berufserfahrung (und insbesondere Programmiererfahrung) waren ausschlaggebend.	q	Bei nur leicht abweichenden Programmiererfahrungen unterscheiden sich die Bewerber:innen v. a. in den Social Skills, die letztlich ausschlaggebend waren.	q	Abschluss und Programmiererfahrung können den leichten Unterschied bei der Berufserfahrung (einziger wichtiger Unterschied) über Zeit wettmachen.	q
1674	bessere Berufserfahrung, mehr Programmierfahrung	q	Mehr Berufserfahrung nötig in der Umgebung	q	sehr viel Berufserfahrung und viele soziale Skills	q
1683	Programmiererfahrung	q	Abschluss, Programmiererfahrung	q	allgemein besser	o
1689	bessere Programmiererfahrungen	q	bessere Sprache und Abschluss	q	bessere Programmiererfahrung	q
1692	Programmier- und Berufserfahrung besser.	q	Gesamtpaket besser. Sprachen bzw. Abschluss sind in diesem Fall nicht so wichtig-	q	Hier wurde der Bewerber mit mehr Berufserfahrung gewählt. Der Abschluss mit 2 Sternen ist wahrscheinlich mit einem ausländischen 3 Sterne zu vergleichen.	q, r
1708	Berufserfahrung & Programmiererfahrung	q	Abschluss & Programmiererfahrung	q	Abschluss & Programmiererfahrung	q
1739	Programmierkenntnisse essentiell	q	Sozial Skills durch Beratung auch relevant	q	Ausgeglichener	o
1741	mehr Berufs- und Programmiererfahrung	q	mehr Social Skills	q	ähnliche gute Optionen	o
1753	mehr Brufserfahrung	q	bessere Programmiererfahrung	q	mehr Berufserfahrung	q
1764	Jünger mit sprachlichen Vorteilen	q, a	Jünger mit sprachlichen Vorteilen	q, a	Mehr Berufserfahrung	q
1773	Berufserfahrung gleich Abschluss aus. Soziale Skills und Programmiererfahrung sind besser.	q	Programmierfahrung ist wahrscheinlich wichtiger als Soziale Skills. Sprachen und Abschluss besser	q	Mehr Programmiererfahrung, besserer Abschluss. Berufserfahrung nur ein Stern schlechter	q
1774	Berufserfahrung wiegt mehr als Abschluss	q	viel Programmiererfahrung	q	mehr Programmiererfahrung	q
1796	mehr Berufs- und Programmiererfahrung	q	Soziale Skills für diesen Job wichtig, und Stephanie Jung hat da mehr Sterne	q	Mehr Berufserfahrung und Programmiererfahrung	q
1802	höhere Erfahrung	q	höhere qen in den Schlüsselelemeten	q	Bessere Programmiererfahrung und besserer Abschluss	q
1803	höherer Abschluss	q	Programmierer haben oft wenig soziale Skills, sind aber im Programmieren sehr gut	q	Besserer Durchschnitt der Werte	q
1825	Mehr Berufserfahrung und mehr Programmiererfahrung. Ich denke, das kompensiert seine mangelnde Sprachkenntnisse.	q	Mehr Berufserfahrung und mehr soziale Skills. Obwohl ein IT-Job auf den ersten Blick keine mega sozialen Skills voraussetzt, ist es für die Teamfähigkeit schon sehr wichtig.	q	Super Abschluss, super soziale Skills und super Programmiererfahrung. Sprachkurs kann sie ja noch machen.	q
1826	mehr berufserfahrung	q	mehr berufserfahurng, sozialer	q	mehr berufserfahrung	q
1848	Ähnliche Kompetenzen, mehr Programmiererfahrung.	q	Kompetenzen unterscheiden sich nicht wesentlich, Frauen sollten in der IT unterstützt werden.	q, g, e	Ist in allem überlegen außer in Berufserfahrung und Sprachen, was vernachlässigbar ist.	q
1854	Programmiererfahrung für diese Stelle sehr wichtig	q	soziale skills bei Kundenkontakt sehr wichtig	q	Berufserfahrung und Programmiererfahrung gleich wichtig	q
			**Condition 1 – Job 2**			
1335	bessere Softskills	q	bessere Softskills	q	bessere Softskills	q
1352	Social Skills	q	bessere Kombination	o	ausgeglicheneres Profil	o
1363	ich wollte eigentlich doch den anderen wählen	x	Soziale skills	q	Der andere hat zu wenig Methodenkenntnis	q
1387	social skills sind wichtig	q	in allem etwas besser	o	in allem etwas besser	q

1402	Berufserfahrung	q	Berufserfahrung, Methodenkompetenz	q	jünger	a
1415	In allen wichtigen Bereichen gut geeignet	q	Soziale Skills sind wichtig für den Job, in Kombination mit gutem Abschluss	q	In allen Bereichen durchschnittlich gut	q
1433	Berufserfahrung, soziale Skills	q	guter Abschluss und genug Erfahrung, gute Methodenkompetent daher geeignet	q	alle Fähigkeiten gut ausgeglichen und sehr gute Methodenkompetenz	q
1439	Berufserfahrung und soziale Skills sind höher bewertet.	q	Methodenkompetenz, Sprache und Berufserfahrung erscheinen als Vorteil im Vergleich zur anderen Bewerberin.	q	Mehr Berufserfahrung und Methodenkompetenz erscheinen als Vorteil im Rahmen der Jobbeschreibung.	q
1452	Jünger und gute Methodenkompetenz	q, a	Gute qen	q	Bessere Methodenkompetenz	q
1473	mehr erfahrung	q	mehr erfahrungen	q	mehr erfahrungen	q
1498	Erfahrung und einer, der Verantwortung gut übernehmen könnte.	q	Genereller Mix der Skills ist besser	o	Erfahrener	o
1501	Jünger und mehr Sterne in den relevanten Bereichen	q, a	jünger	a	Zwar weniger Methodenkompetenz aber man kann dies erlernen, da Bewerber noch sehr jung ist.	q, a
1514	Hohe Berufserfahrung und Social Skills sprechen für solide Aufgabenbewältigung	q	Social Skills	q	Jünger Kommunikationstalent aufgrund von Sprachen und ausgeprägte soziale Skills	q, a
1524	sehr guter Abschluss und Methodenkompetenz, Alter spielt eine Rolle	q, a	in den meißten Punkten der anderen Kandidatin überlegen	q	Methodenkompetenz und Berfuserfahrung überzeugen	q
1532	Besser	o	Soziale Skills sind besser	q	Hier haben die andere sachen überwogen auch wenn social skills schlechter war	q
1535	Ohne Gescheiten Abschluss und ohne Informatikstudium weiß der eh nicht von was der redet	o	Wer Projektleitung machen will braucht auch gute soziale Fähigkeiten	q	Der typ ist die beste Mischung. Bei den Nerds ist das eh egal. Du fütterst die mit Clubmate dann passt das schon	o
1550	Mehr Berufserfahrung, 2 Mehr Soziale Skills	q	Mehr Methodenkompetenz und Berufserfahrung.	q	Methodenkompetenz	q
1551	jung und gut ausgebildet	q, a	im direkten vergleich jung und gut ausgebildet	q, a	Allrounder	o
1567	Die Methodenkompetenz ist höher als beim Mitbewerber. Die Soziale Kompetenz ist zwar geringer ausgeprägt aber dafür verfügt dieser Bewerber zusätzlich über einen qualifizierteren Abschluss	q	Das Profil scheint ausgeglichener als das der Mitbewerberin.	o	Die Methodenkompetenz der Mitbewerbers ist zu gering.	q
1580	guter abschluss	q	Guter Abschluss	q	jünger	a
1589	BE + soziale Skills besser als 1 fehlender Stern bei Methodenkompetenz	q	Methodenkompetenz	q	methodenkompetenz	q
1597	Berufserfahrung, Soziale Skills und Methodenkompetenz bewerte ich für den Job als relevanter. Bewerber Ackermann hat hier mehr Kompetenzen	q	Bessere Berufserfahrung, Methodenkompetenz und mehr Spracherfahrung	q	gleichmäßigere Kompetenzen in allen Bereichen bei Lehmann	q
1623	Thomas hat im Hinblick auf Methodenkompetenz, soziale Skills und Berufserfahrung mehr Sterne	q	In Bezug auf Methodenkompetenz und soziale Skills bei Monika mehr Sterne	q	Um eineiges mehr Methodenkompetenz	q

1625	Berufserfahrung und soziale Skills gut	q	soziale Skills wichtig	q	Methodenkompetenz und Berufserfahrung wichtig	q
1639	Mehr Berufserfahrung von Vorteil	q	Mehr Berufserfahrung und bessere Methodenkompetenz machen Bewerber geeigneter	q	Methodenkompetenz in diesem Bereich sehr wichtig. Höhere Berufserfahrung vorteilhaft.	q
1643	Abschluss und Methodenkompetenz überdurchschnittlich. Soziale Fähigkeiten sind ausbaufähig.	q	Höhere Berufserfahrung; bessere Methodenkompetenz.	q	Höhere Werte in Berufserfahrung und Methodenkompetenz.	q
1651	Gesamtprofil stimmiger. Abschluss weniger relevant als Berufserfahrung	q	Höhere Berufserfahren. Social Skills könnte man schulen.	q	Hohe Methodenkompetenz und höhere Berufserfahrung	q
1658	Trotz hohen Alters, bessere Kompetenzen als Mitstreiter!	q, a	keine Angabe	x	Potential erkennbar im Bereich der Kompetenzen!	q
1673	Der Bewerber scheint mehr praktische Erfahrung versus überwiegend theoretischer des anderen mitzubringen.	q	Methodenkompetenz i. V. m. der Berufserfahrung waren ausschlaggebend gegenüber hauptsächlich theoretischen Uni(?)-Kenntnissen.	q	Sprachenkenntnisse (des anderen Bewerbers) überwiegen trotz ihrer Exzellenz auf keinen Fall die Methodenkompetenz (und Berufserfahrung) des anderen.	q
1674	viel Erfahrung, was den schlechten Abschluss aufwiegt	q	sehr guter Abschluss, Methodenkompetenz kann sie lernen	q	mehr Erfahrung	q
1683	Erfahrung und Soziale Skills	q	Alter	a	der andere hat zu wenig Methodenkompetenz	q
1689	guter Abschluss und Methodenkompetenz	q	Bessere Methodenkomptenze und mehr Berufserfahrung	q	jünger, bessere soziale Skills	q, a
1692	Obwohl älter, sind hier doch die Sterne bei sozialen Skills und Berufserfahrung entscheidend für meine Entscheidung.	q, a	Berufserfahrung kann noch gesammelt werden (Altersvergleich) und Methodenkompetenz gesammelt (besserer Abschluss). Trotz des besseren Abschluss sind auch die Social skills besser.	q, a	Die Methodenkompetenz ist in diesem Fall ausschlaggebend.	q
1708	Berufserfahrung & soziale Skills	q	Berufserfahrung & Methodenkompetenz	q	Berufserfahrung & Methodenkompetenz	q
1739	Berufserfahrung > Abschluss	q	Ausgeglichenere Punktzahl	q	Ausgeglichener	o
1741	bessere Social Skills	q	höhere Social Skills	q	wenig Berufserfahrung und wenig Methodenkompetenz beim anderen Kandidaten	q
1753	höhere Berufserfahrung	q	bessere Methodenkompetenz	q	bessere Methodenkompetenz	q
1764	Alter ist für Projektmanagement geeigneter	a, o	Soziale Skills der Alternative zu gering	q	Soziale Skills der Alternative zu gering	q
1773	Mehr Berufserfahrung könnte den Abschluss aufwiegen. Soziale Skills sind besser.	q	Schwierige Entscheidung. Gleiche Anzahl an Sternen, aber die Verteilung gefällt mir etwas besser.	o	Sprachen sind nicht so wichtig, Methodenkompetenz deutlich besser.	q
1774	Soziale Skills wichtig für die Position	q	Soziale Skills wichtig für die Position	q	Soziale Skills wichtig für die Position, Methodenkompetenz kann evtl. aufgrund seines Alters noch ausgebaut werden	q, a
1796	viel Berufserfahrung und soziale Skills	q	bleibt wahrscheinlich längere m Unternehmen, da jünger	a	Martin Bach ist noch jung und kann durch seine guten sozialen Skills die fehlende Methodenkompetenz ausgleichen bzw. erlernen	q, a
1802	auch hier überwiegen soziale Skills, Methodenkompetenz und zstzl die Berufserfahrung	q	insgesamt bessere q verteilt auf die verschiedenen Bereiche	q	Methodenkompetenz als Schlüsselqualifikation besser ausgeprägt	q

1803	jünger	a	guter Abschluss, jung	q, a	Methodenkompetenz besser	q
1825	Mehr Berufserfahrung und mehr soziale Skills	q	mehr soziale Skills. die geringere Methodenkompetenz kann auch mit der geringeren Berufserfahrung zusammenhängen	q	Sehr schwierige Entscheidung. Aber ich denke man kann an der Methodenkompetenz (durch Schulungen, etc.) noch besser arbeiten als an den social skills	q
1826	bessere methodenkompetenz	q	bessere methodenkompetenz	q	bessere methodenkompetenz	q
1848	Soziale Kompetenz ist für das Projektmanagement sehr wichtig.	q	Soziale Skills sind wichtig und eher nicht erlernbar, anders als die Methodenkompetenz.	q	Soziale Skills sind unabdingbar.	o
1854	gute Mischung aus allem	q	Methodenkompetenz wichtig	q	Methodenkompetenz etwas wichtiger als soziale skills	q
			**Condition 1 – Job 3**			
1335	bessere Soft Skills	q	bessere Softskills	q	bessere Softskills	q
1352	hat wichtigere Skills	o	mehr Softwareskills	q	Abschluss besser	q
1363	social skills sind hier nicht so wichtig	q	social skills sind hier nicht so wichtig	q	mehr Erfahrungen	q
1387	alles sehr solide	o	abschluss	q	social skills und abschluss	q
1402	soziale Skills	q	weiblich	g	berufserfahrung, weiblich	q, g
1415	Durchschnittlich in jedem Bereich und daher vermutlich gut anpassungsfähig und lernbereit	q, o	Bessere soziale Skills & Berufserfahrung	q	Guter Abschluss und Erfahrung mit Software	q
1433	Abschluss besser	q	gut ausgewogene Fähigkeiten	q	Erfahrung und soziale Skills besser	q
1439	Mehr Softwareerfahrung erscheint als Vorteil.	q	Höhere Bewertung der Softwareerfahrung erscheint als Vorteil.	q	Berufserfahrung erscheint hier als Vorteil.	q
1452	Gute Softwareskills und Social Skills	q	Mehr Softwareerfahrung	q	Jünger und bessere Softwareerfahrung	q, a
1473	bessere Software Skills	q	besseren Abschluss	q	soziale skills sind auch wichtig	q
1498	Beide Kandidaten sind ähnlich gut, aber der 2. Kandidat ist leicht besser	o	Beide Kandidaten ähnlich gut	o	Soziale SKills und Berufserfahrung	q
1501	mehr sterne an den relevanten stellen	q	qualifizierter	o	relevantere Kompetenzen	o
1514	bessere erfahrung	q	wieder bessere erfahrung, social skills unwichtig bei einem buchhalter	q	jünger und mehr erfahrungen	q, a
1524	Super Abschluss und Softwareerfahrung, social skills sind für die Buchhaltig nicht relevant	q	guter Abschluss, viel Berufserfahrung und Softwarekenntnis	q	sehr guter Abschluss und Softwarekenntnis	q
1532	Bessere Soziale Skills und beruferfahrug	q	Gesamt einfach besser	o	Wegen Software erfahrung und abschluss	q
1535	Die hat die größte Fachkompetenz	o	Solange der keine Leitung macht ist es egal	o	Buchhalter brauchen nur Rechungslegungswissen.	o
1550	Soziale Skills eher unwichtig	q	Softwareerfahrung ist besser	q	Bessere Softwareerfahrung	q
1551	Social Skills	q	Allrounder	q	Abschluss und Erfahrung	q
1567	Dieses Profil erscheint ausgeglichener.	o	Dieser Bewerber verfügt über bessere Softwareerfahrung.	q	Bessere Softwareerfahrung	q
1580	soziale skills	q	Ausgewogenere fähgkeitenverteilung	q	guter abschluss	q
1589	breiter aufgestllt	q	Softwareerfahrung	q	Softwareerfahrung	q

1597	bessere Softwareerfahrung, Abschluss und Sprachen	q	bessere Voraussetzungen laut KI bei Meyer	q	mehr Berufserfahrung bei Ostermann	q
1623	Über Softwarerfahrung, Berufserfahrung und Abschluss hinweg gesehen mehr Punkte	q	Mehr Sterne in Bezug auf Softwareerfahrung und Berufserfahrung	q	Über Softwareerfahrung, Berufserfahrung und Abschluss hinweg gesehen mehr Punkte	q
1625	soziale skills hier nicht so wichtig, in anderen Kategorien war sie besser	q	Softwareerfahrung wichtiger als soziale Skills	q	Softwareerfahrung am wichtigsten	q
1639	Sprache und Abschluss höher eingestuft	q	Sprache einen Stern mehr, insgesamt ausgeglichene Profil	q	Hoher Abschluss, bessere Sprachkenntnisse	q
1643	Höhere Werte in Software- & Berufserfahrung	q	Höherer Wert in Softwareerfahrung	q	Höherer Wert in Softwareerfahrung	q
1651	mehr oder minder gleichwertige qen - Frau vorgezogen.	q, e	höhere Softwareerfahrung - kommt schneller rein	q	mehr Berufserfahrung	q
1658	Soziale Fähigkeiten nicht stark gefragt, deswegen mehr Wert auf Erfahrung und Umgang mit Computern!	q	Höhere Sprachkentnisse und besserer Gesamtabschnitt!	q	Höhere Berufserfahrung!	q
1673	Soziale Skills müssen nicht unbedingt hervorragend sein. Wichtiger ist der Abschluss und Softwareerfahrung i. V. mit Berufserfahrung.	q	Bei gleicher Berufserfahrung ist der gute Abschluss hier wichtiger als Softwareerfahrung.	q	Leichte Tendenz aufgrund des Abschlusses, aber sonst relativ ausgewogen.	q
1674	bisher weniger Beufserfahrung dafür mehr softwareerfahrung und guten Abschluss kann sich als schnelle neues aneignen	q	sehr viel Efahrung inklusive gutem Abschluss, soziale Skills für die Position weniger wichtig	q	sehr guter Abschluss, schon softwareerfahrung.	q
1683	Bauchgefühl	o	Erfahrung	q	Abschluss und Softwareerfahrung	q
1689	besserer Abschluss und mehr Softwareerfahrung	q	besserer Abschluss und etwas jünger	q, a	besserer Abschluss	q
1692	Hier waren auch Abschluss und Softwareerfahrung entschieden, ansonsten schwere Entscheidung in diesem Fall.	q	Auch hier eher Gleichstand, deswegen für den Bewerber mit besseren Social Skis entschieden .	q	Trotz weniger Berufserfahrung bessere Softwareerfahrung.	q
1708	Weiblich, Abschluss, Softwareerfahrung	q, g	besserer Abschluss, gleich hohe Berufserfahrung	q	Berufserfahrung, Soziale Skills	q
1739	Mehr Punkte	x	Ausgeglicheneres Profil	q	Bei ähnlichen qen Bevorzugung von Frauen	q, e
1741	etwas mehr Berufserfahrung	q	mehr Softwareerfahrung	q	beide ähnlich gut	q
1753	mehr Softwareerfahrung	q	mehr Softwareerfahrung	q	mehr Berufserfahrung	q
1764	Etwas bessere Fachqualifikation	q	Männlich	g	Bessere Fachqualifikation	q
1773	Beide gleich, 5-Sterne-Bewertung sieht besser aus.	o	relativ gleich verteilt, aber Softwareerfahrung wichtiger als Soziale Skills.	q	Sprache und Abschluss gleichen Berufserfahrung aus. Softwareerfahrung wichtiger als Soziale Skills.	q
1774	Berufserfahrung zählt mehr und Schwäche in Softwareerfahrung wird durch hohe Soziale Skills ausgeglichen	q	mehr Softwarerfahrung	q	mehr Softwareerfahrung und besserer Abschluss	q
1796	hat bei Kombi aus Berufserfahrung & Sozialen skills insgesamt mehr Sterne als Katharina Decker	q	besserer Abschluss und mehr soziale Skills - ist eine Frau -> bisher wurden mehr Männer eingeladen	q, e, g	mehr Berufserfahrung und soziale Skills & weiblich	q, g

1802	soziale Skills und Softwarerefahrung als Schlüsselqualifikation höher	q	bessere Verteilung der wichtigen qen	q	bessere soziale Skills und Softwarerefahrung	q
1803	Abschluss ist besser	q	Soziale Skills > Softwareentwicklung	q	besserer Abschluss	q
1825	super soziale Skills und Berufserfahrung ist auch gut. Softwareerfahrung soll mittels training on the job verbessert werden	q	super Abschluss, Berufserfahrung, Soziale Skills. Softwareerfahrung soll im Job verbessert werden	q	Mehr Berufserfahrung und mehr soziale Skills	q
1826	Mehr berufserfahrung und gute soziale Skills und bessere softwarerfahrung	q	bessere soziale skills	q	mehr berufserfahrung	q
1848	In Sozialem deutlich überlegen, Softwareerfahrung lässt sich nachholen, Rest ist Mittelfeld, ähnlich wie bei Decker.	q	Geben sich im Ganzen nicht viel. Ältere Frauen sollten unterstützt werden.	q, e, g	Soziale Skills sind schwerer zu gewichten als der Abschluss	q
1854	Bauchgefühl besser geeignet	o	überall Top	q	Berufserfahrung genauso wichtig wie Abschluss	q

Table C2 Candidate Selection Reasons of Participants in Condition 2

ID	Reason 1	C	Reason 2	C	Reason 3	C
			**Condition 2 – Job 1**			
1344	Mehr Berufserfahrung; mehr Programmiererfahrung	q	Mehr soziale Skills	q	bessere Programiererfahrung und besserer Abschluss	q
1354	Programmiererfahrung, Soziale Skills, Berufserfahrung	q	Programmiererfahrung	q	Programmiererfahrung & soziale Skills	q
1355	er hat mehr Programmiererfahrung und auch Berufserfahrung was hier sehr wichtig ist	q	mehr Programmiererfahrung; Social Skills sind meines Erachtens in diesem Bereich nicht so wichtig	q	Berufserfahrung	q
1370	Programmiererfahrung	q	Programmiererfahrung	q	Programmiererfahrung	q
1397	Überzeugt durch bessere Programmierführung	q	Gute Programmierführung	q	Überzeugt durch Soziale Skills und Programmierführung.	q
1401	Mehr Berufserfahrung, sonst meist identisch. Viele Sprachen sind für den Beruf nicht bedeutsam.	q	Programmiererfahrung	q	Abgesehen von Sprachen meist besser	q
1421	Programmieren und Berufserfahrung	q	Ausbildung und Programmieren	q	Abschluss, Programmieren	q
1422	Mehr Erfahrung und Programmierskills	q	Abschluss besser und Programmierskills	q	Programmiererfahrung höher	q
1450	berufserfahrung & programmiererfahrung	q	ratio programmiererfahrung und social skills	q	berufserfahrung	q
1470	mehr programierungserfahrung	q	besseres profil insgesamt	q	erfahrung , sozial und programmiererfahrung besser	q
1492	Zwar Türke aber dennoch mehr IT-Erfahrung.	q, r	Frau ist immer gut für die Teammoral.	g	keine Angabe	q
1511	Kandidat scheint besser zu sein	o	Subjektiv besser	o	Kandidatin passt besser in die Stellenbeschreibung	o
1517	Berufserfahrung über Abschluss	q	Bessere Verteilung der Skills	q	Verteilung der Skills	o

1525	Mehr Berufs und Programmierfahrung	q	Mehr Berufserfahrung und Soziale Skills	q	Mehr Beruferfahrung und sprachliche Kenntnisse	q
1530	Berufserfahrung und Programmierung	q	Soziale skills	q	Soziale Skills und Programmiererfahrung	q
1541	Mehr Programmiererfahrung und Berufserfahrung.	q	Durchschn. bessere Kompetenzen.	q	Mehr Programmiererfahrung.	q
1548	bessere Programmiererfahrung	q	bessere Programmiererfahrung und Sprachen	q	sehr ähnlich: bessere Programmiererfahrung	q
1568	höhere Programmiererfahrung wichtig ansonsten sehr vergleichbar	q	soziale Skills besser, das ist wichtig Programmiererfahrung nur gering schlechter, trotzdem auf mittelmäßigem Niveau	q	Die Bewerberin ist in sozialen Skills und Programmiererfahrugn gut ausgestattet. Auch mehr Berufserfahrung.	q
1576	Bessere Sprachkentnisse	q	Limitierte Soziale Skills und Berufserfahrung	q	Bessere Sprache und Berufserfahrung	q
1590	Berufserfahrung, Programmiererfahrung	q	Soziale Skills --> wichtig für Kommunikation mit Belegschaft, Berufserfahrung	q	bessere Sprachkenntnisse, höhere Erfahrungen	q
1611	Programmier- und Berufserfahrung	q	Berufserfahrung und soziale Skills	q	ein gewisses Sprachniveau hier schon wichtig	q
1622	ist besser	o	wirkt kompetenter	q	ist besser	q
1632	Berufserfahrung wichtig für Unterstützung der restlichen Belegschaft	q	höhere Berufserfahrung, soziale Skills wichtig im Umgang mit anderen Mitarbeitern	q	gute Sprachkenntnisse erforderlich, um Mitarbeiters weiterhelfen zu können	q
1636	mehr Programmiererfahrung	q	seine sozialen Fähigkeiten sind besser	q	Sozialkompetenz und Programmiererfahrung	q
1644	höhere Programmiererfahrung	q	social Skills + programmier Erfahrung höher und wichtiger --> Gesamtpaket	q	sonst Sprachbarrieren	q
1653	höhere Programmiererfahrung	q	sehr guter Abschluss und Programmiererfahrung	q	Programmiererfahrung und Abschluss besser, auch wenn Berufserfahrung geringer	q
1660	Insgesamt haben die Bewerber gleich viele Sterne, der gewählte hat mehr bei Programmiererfahrung, was ich für relevanter für den Job halte als Sprachen.	q	Beide haben gleich viele Sterne, Frauen sind in technischen Jobs unterrepräsentiert - bei gleicher q wähle ich deshalb die Frau.	q, e, g	Soziale Skills und Programmiererfahrung finde ich wichtiger für den Job als Sprache und allgemeine Berufserfahrung.	q
1672	Geringere Spachen und Abschluss, dafür mehr Berufs- und Programmiererfahrung	q	Größere Sprach- und Programmierfähigkeiten	q	ebenfalls besseres Gesamtpaket; Berufserfahrung wird mit der Zeit kommen	q
1675	Programmiererfahrung ist besser ausgebaut	q	Schwerpunkt liegt auf Sozialen Skills und Programmiererfahrung. Dies ist vermutlich wichtig bei dem Beruf.	q	Soziale Skills und Programmiererfahrung sind sehr gut ausgeprägt.	q
1685	Mehr Soziale Skills, Programmiererfahrung und Berufserfahrung	q	Mehr soziale Skills und Berufserfahrung	q	Mehr Berufserfahrung, besser in Sprachen	q
1688	bessere Programmier- und Berufserfahrung	q	höhere Programmiererfahrung	q	bessere Programmiererfahrung	q
1691	Mehr Berufserfahrung	q	Stärke im Programmieren	q	Bessere q	q
1718	ist besser	o	Die ist qualifizierter	o	wirkt allgemein besser	o
1745	Mustafa Özdemir hat mehr Berufs- und Programmiererfahrung	q	Stephanie Jung hat mehr Berufsefahrung, soft skills und Programmiererfahrung, die für diese Ausschreibung relevant sind.	q	Miray Korkmar hat einen besseren Abschluss	q
1746	Programmierkenntnisse und Berufserfahrung	q	Abschluss und Berufserfahrung	q	Sprachen ist sehr ungenau, aber ein Stern lässt nichts Gutes vermuten. Englisch sollte schon drin sein.	q

1757	Mehr BE und mehr Programmiererfahrung.	q	mehr Programmiererfahrung, besserer Abschluss	q	Ausgewogener	q
1768	Gesamtpaket besser	o	Prgrammieren kann man lernen	o	sprache und Berufserfahrung vorn Programmieren	q
1770	Berufserfahrung und Programmiererfahrung besser bei gleichen sozialen Skills	q	Deutlich bessere soziale Skills für anwenderspezif. Fragestellungen wichtig	q	Schwierig, annähernd gleich. Bessere Programmiererfahrung war ausschlaggebend, Sprachen und Berufserfahrung kann noch wachsen.	q
1780	mehr Programmierskills	q	mehr IT Skills	q	überall besser außer in Sprachen, was nicht wichtig ist	q
1791	Viel Berufserfahrung	q	Frau Sahin hat zu wenig Berufserfahrung	q	Berufserfahrung, soziale Skills	q
1806	Praktische Erfahrung in diesem Bereich wichtiger als Abschluss	q	Bereits mehr Berufserfahrung und Programmiererfahrung	q	sehr starke Programmiererfahrung	q
1817	höhere Programmiererfahrung und höhere Berufserfahrung sehe ich als wichtige Kriterien an für die IT-Administration	q	Programmiererfahrung und Berufserfahrung sind vergleichbar (Achtung, Rechtschreibfehler : Programmierfahrung) bei beiden BewerberInnen; den Ausschlag für S. Jung gab die höheren sozialen Skills, die bei der Unterstützung der Belegschaft bei anwenderspezifischen Fragestellungen für mich ein wichtiges Kriterium sind	q	Programmiererfahrung als oberste Priorität ist leicht erhöht. Beide Bewerberinnen sind gleich jung, daher ist die Berufserfahrung etwas zu vernachlässigen. Leicht höherer Abschluss könnte eventuell mehr Potential entfalten in Ausübung dieser Stelle. Soziale Skills sind gleich stark ausgeprägt.	q
1823	Berufserfahrung & Programmiererfahrung wichtiger als Abschluss in Anwendungsbezogenem Bereich	q	soziale skills wichtig wegen Interaktion mit Belegschaft, Sprachen scheinen generell weniger wichtig	q	mehr Berufserfahrung - Sprache scheint wichtig wegen Intraktion mit Belegschaft	q
1828	Programmiererfahrung, Sprache	q	Sprache, Programmiererfahrung	q	Sprachen, Berufserfahrung	q
1830	Mehr Berufs- und Porgrammiererfahrung	q	Social Skills besser für die Zusammenarbeit mit Belegschaft	q	mehr Berufserfahrung	q
1850	höherer Abschluss und höhere q in Sprachen, könnte bei Serveradministration von Kunden wichtig sein für den Austausch	q	mehr Programmiererfahrungen und qen in Sprachen	q	mehr sprachliche q bei gleichen sozialen Skills	q
1851	Mehr Berufs- und Programmiererfahrung.	q	Große Programmiererfahrung und besserer Abschluss.	q	Eigenschaften besser bewertet als bei Konkurrenz	q
			**Condition 2 – Job 2**			
1344	mehr Berufserfahrung	q	besseres Gesamtbild	o	bessere Kommunikationsfähigkeit	q
1354	Soziale Skills & Berufserfahrung	q	Soziale Skills & Alter	q, a	Allroundpaket	o
1355	Berufserfahrung und soziale Skills	q	Methodenkompetenz und Berufserfahrung	q	Methodenkompetenz	q
1370	Methodenkompetenz	q	Alter, aber schlechte Methodenkompetenz, unsicher	q, a	Methodenkompetenz	q
1397	Überzeugt durch Berufserfahrung, Soziale Skills und Methodenkompetenz	q	Überzeugt durch Soziale Skills, Berufserfahrung. Relativ jung, Kompetenzen können gut weiter ausgebaut werden.	q, a	Wesentlich besser Methodenkompetenz	q
1401	Soziale Skills bei der Stelle wichtig	q	Soziale Skills wichtig + jünger	q, a	durchschnittlich besser	q

1421	gute Sozialkompetenz in Verbindung mit Berufserfahrung, dabei ausreichend Methodenkompetenz	q	die Mischung aus alter, Berufserfahrung, Sozial Skills und Methodenkompetenz	q, a	hohe Methodenkompetenz	q
1422	besserer Abschluss und Mehodenkompetenz	q	Mehr Berufserfahrung und Methodenkompetenz	q	Mehr Mehodenkompetenz und Berufserfahrung	q
1450	rundum gut, jünger	q, a	jünger und gutes Profil	q, a	methoden	q
1470	der bewerber ist jünger	a	bewerber ist jünger	q, a	insgesamt besser	o
1492	Gute Skills in den wichtigen Bereichen.	q	Bessere Bewertungen.	o	Durchschnittlich stabil.	o
1511	Anderer ist eventuell schon zu alt, um zukunftsorientiert zu handeln	q	Kandidatin scheint besser zu sein (Soziale Skills), scheint jung und dynamisch	q, a	subjektiv besser	o
1517	Mehr Berufserfahrung soziale skills	q	Alter und Abschluss	q, a	Skills besser verteilt, Sprachen weniger wichtig	q
1525	Mhr Beruferfahrung und soziale Skills	q	Soziale Skills wichig	q	Hohe Methodenkompetenz	q
1530	Berufserfahrung und soziale skills	q	Soziale sills gekoppelt mit Berufserfahrung	q	Methodenkompetenz und Berufserfahrung	q
1541	Soziale Skills und Erfahrung.	q	Fehlende Kompetenzen sind erlernbar.	q	Hohe Methodenkompetenz.	q
1548	Verteilung des Könnens(Sterne) ist besser verteilt	q	sehr ähnlich, deswegen Fokus auf Abschluss und Berufserfahrung gelegt	q	besser in Methodenkompetenz	q
1568	viel Berufserfahrung und soziale Skills. Lediglich die Methodenkompetenz ist marginal schlechter	q	Die Methodenkompetenz ist besser. Die sozialen Skills sind nicht so hoch, jedoch kann viel Berufserfahrung hilfreich sein	q	vergleichbare soziale Skills, aber höhere Methodenkompetnez und Beruserfahrung	q
1576	Soziale Skills sind als Projektleiter wichtig	q	Mehr Berufserfahrung	q	Ausgeglicheneres Profil	q
1590	hohe Berufserfahrung --> Mehrwert für junge Mitarbeiter, hohe soziale Skills --> nützlich um Erfahrung zu vermitteln	q	bessere Sprachkenntnisse und Berufserfahrung sowie Methodenkompetenz	q	hohe Sprachkenntnisse, soziale Skills ermöglichen gute Kommunikation unter Mitarbeitern, methodenkompetenz muss ausgebaut werden	q
1611	bessere Methodenkompetenz bei mehr Berufserfahrung, jünger	q, a	gute soziale Skills, jünger	q, a	hauptsächlich wegen Methodenkompetenz	q
1622	wirkt geeigneter für den job	o	soziale Skills wichtig, die andere ist zu alt	q, a	wirkt besser	o
1632	geringerer Abschluss wird durch sehr hohe Berufserfahrung ausgeglichen	q	höhere Berufserfahrung und Methodenkompetenz	q	gute Methodenkompetenz	q
1636	bessere soziale Kompetenz und Erfahrung	q	bessere Fähigkeiten	q	bessere Fähigkeiten	q
1644	Berufserfahrung + social skills	q	fast identisch	o	Methodenkompetenz	q
1653	Allrounder, besonders mit hohen sozialen Skills	q	Allrounder	o	Zwar weniger soziale Skills, jedoch sollte ein Mindestmaß an Methodenkompetenz vorhanden sein	q
1660	Insgesamt vergleichbare Bewertung, der Bewerber hat aufgrund seines Alters womöglich keinen modernen Abschluss, dafür ausgewogen hohe Kompetenz in den letzten drei Feldern, die mir am wichtigsten erscheinen.	q, a	Die Bewerberin ist im Vergleich jünger, die Methodenkompetenz relativ leicht zu erwerben. Insgesamt ist die q gleich, diese Bewerberin hat Ausbaupotenzial in den schneller und leichter zu schulenden Bereichen.	q, a	Insgesamt vergleichbare Bewertung, der gewählte Bewerber hat ein ausgewogeneres Kompetenzprofil.	q
1672	mehr erfahrung, besser im sozialen	q	sozial stärker, leicht höherer abschluss	q	mehr erfahrung und kompetenzen bei gleichwertigem abschluss	q

1675	Soziale Kompetenz ist in diesem Berufsfeld wichtig.	q	Der Altersunterschied war sehr hoch und der Beruf erfordert wahrscheinlich hohen Einarbeitungsaufwand.	q, a	Die Methodenkompetenz des anderen Bewerbers ist zu gering	q
1685	mehr Berufserfahrung und Soziale Skills	q	jünger, Defizite noch erlernbar (so wie Methodenkompetenz)	q, a	Mehr Berufserfahrung und Mehtodenkompetenz	q
1688	hohe Berufserfahrung und gute soziale Skills	q	höhere soziale Skills	q	höhere Berufserfahrung und Methodenkompetenz	q
1691	Berufserfahrung super	q	Bessere sozial skills	q	bessere Methodenkompetenz	q
1718	sind beide ähnlich gut	o	andere ist vielleicht zu alt für den job, würde ich eher nicht einstellen	q, a	wirkt besser als der andere	o
1745	Thomas Ackermann hat mehr Erfahrung in allen wichtigen Skills (Berufserfahrung, soziale Skills, Methodenkompetenz)	q	Lisa Schaefer hat insgesamt das bessere Profil	o	Martin Bach hat zwar weniger Methodenkompetenz, kann dies aber durch sein junges Alter aufholen	q, a
1746	Methoden und Abschluss	q	Abschluss	q	Methodenkompetenz wichtig	q
1757	Ausgewogener	o	Mehr Methodenkompetenz und Berufserfahrung.	q	Höhere Methodenkompetenz.	q
1768	Entwicklungsfähiger	o	viel Erfahrung und gute Methodenkompetenz	q	hat potential	o
1770	Schwierig - bessere Methodenkompetenz vs. weniger soziale Skills, knappe Entscheidung für die bessere Methodenkomp.	q	Soziale Skills deutlich vorne, im Rest mehr oder weniger ähnlich. Berufserfahrung immer noch ausbaufähig und damit auch Methodenkompetenz	q	Deutlich besser bei Berufserfahrung und Methodenkompetenz, Sprache nicht ganz so wichtig	q
1780	soziale skills besser	q	überall besser außer in sozialen skills	q	wirkt kompetenter	o
1791	Berufserfahrung und soziale Stils helfen dem Team	q	Methodenkompetenz kann man durch Zusammenarbeit mit anderen erlernen, soziale Kompetenz eher nicht	q	ausgeglichene Kompetenzen	q
1806	Bessere Mischung aus soft und hard skills	q	soziale skills besser + in berufserfahrung hinterher weil deutlich jünger	q	Overall bessere Werte	q
1817	soziale Skills und Methodenkompetenz höher (7 Sterne vs 6 Sterne), dazu mehr Berufserfahrung.	q	soziale Skills und Methodenkompetenz sind insgesamt höher ausgeprägt. die niedrigere Berufserfahrung kann aufgrund des deutlich niedrigeren Alters der Bewerberin vernachlässigt werden, immerhin mit 3 von 5 Sternen überdurchschnittlich!	q, a	soziale Skills und Methodenkompetenz sind höher, dazu Berufserfahrung höher.	q
1823	Soziale Skills bei Alternative deutlich schlechter	q	trotz deutlich jünger ähnliche q + potenzial	q, a	hohe methodenkompetenz	q
1828	hohe soziale Skills und mehr Berufserfahrung	q	deutlich mehr soziale Skills und höherer Abschluss	q	deutlich! höhere Methodenkompetenz	q
1830	Abschluss und Methodenkompetenz	q	Abschluss, Berufserfahrung Methoden	q	Berufserfahrung Mehtode	q
1850	Etwas weniger Methodenkompetenz, dafür deutlich höhere soziale Skills und mehr Berufserfahrung	q	auch hier wieder deutlich mehr soziale Skills bei ähnlicher Berufserfahrung und Medienkompetenz	q	Zwar deutlich weniger soziale Skills als anderer Bewerber, aber anderer Bewerber hat kaum Methodenkompetenz, was als essentiell aus der Jobbeschreibung hervorgeht	q

1851	Besserer Abschluss und Kompetenz	q	Etwas mehr Erfahrung und Kompetenz	q	Mehr Erfahrung und Kompetenz	q
			**Condition 2 – Job 3**			
1344	besseres Gesamtbild	o	besseres Gesamtbild	o	mehr Erfahrung mit Software	q
1354	Abschluss & Softwareerfahrung	q	Abschluss, Berufserfahrung, soziale Skills	q	Abschluss & Softwareerfahrung	q
1355	Softwareerfahrung und Abschluss	q	Softwareerfahrung wcihtiger als soziale Skills	q	Softwareerfahrung	q
1370	Softwareerfahrung	q	Softwareerfahrung	q	Softwareerfahrung	q
1397	Im Durchschnitt besser. Der andere Bewerber hat nur durch seine Sozialen Skills überzeugt, die für die Buchhaltung nicht wesentlich sind.	q	Überzeugt durch Berufs- und Softwareerfahrung	q	Durchschnittliche Berufs- und Softwareerfahrung	q
1401	unsicher warum	o	meist besser	o	mehr Erfahrung	q
1421	besserer Abschluss, Softwarekenntnis	q	Stimmiges gesamt Paket: guter Abschluss ausreichend Berufserfahrung, soziale Skills	q	Software, besserer Abschluss	q
1422	Soziale skills ausgeprägter	q	leicht bessere Schwerpunkte (Abschluss+soziale Skills)	q	Soziale Skills überzeugen	q
1450	software	q	insgesamt besser	o	softwareerfahrung	q
1470	soziale skills im plus , sowie berufserfahrung	q	hätte hier beide eingeladen, habe aber fr. richter wegen der sozialen skills letztendlich gewählt	q	mehr berufserfahrung sowie sozial weiter	q
1492	Social Skill Experten sind immer nützlich für das Unternehmen	q	Alles in allem ein Genie was seine Kompetenzen angeht. Das schmeckt.	q	Eine Frau fehlt im Team	e, g
1511	Kandidat ist besser	o	Ist geeigneter	o	Kandidat ist besser, bspw sind Social Skills nicht so wichtig	q
1517	Soziale skills höher	q	Softwareerfahrung im Vergleich weniger wichtig	q	Mehr Berufserfahrung und soziale Skills	q
1525	Mehr Berufserfahrung und Soziale Skills	q	Mehr Softwareerfahrung	q	Mehr Berufserfahrung und Soziale Skills	q
1530	Softwareerfahrung	q	Soziale Skills und Berufserfahrung	q	Soziale Skills und Berufserfahrung	q
1541	Besserer Abschluss und mehr Erfahrung mit Software. Berufserfahrung unterscheidet sich nicht wesentlich.	q	Mehr Softwareerfahrung.	q	Mehr Softwareerfahrung.	q
1548	Verteilung ausgeglichener	q	mehr Softwareerfahrung	q	mehr soziale skills	q
1568	guter Abschluss - Berufserfahrung kann noch gesammelt werden - Softwareerfahrung ist besser im Vergleich - soziale skills nicht so wichtig	q	Der Bewerber hat zwar einen leicht schlechteren Abschluss, jedoch weist er bessere Softwareerfahrung auf. Soziale Skills sind nicht so relevant	q	Softwareerfahrung besser soziale Skills weniger wichtig der gute Abschluss kann ggf. die Berufserfahrung ausgleichen	q
1576	Bessere Softwareerfahrung	q	Ein Abschluss alleine sagt nichts über die Fähigkeiten	q	Berufserfahrung kann erworben werden.	o
1590	Soziale Skills, Berufserfahrung	q	Softwareerfahrung, Sprachen, Berufserfahrung	q	höherer Abschluss, bessere Sprachkenntnisse, Software	q
1611	soziale Skills hier nicht so wichtig, ansonsten sehr ähnlich, aber Auswahl der Frau, da bis jetzt vor allem Männer genommen wurden	q, g, e	sehr gute Bewertung außer in sozialen Skills, die hier nicht so wichtig sind	q	ähnliche Bewertung, aber mehr Berufserfahrung + Frau	q, g
1622	ist kompetenter	o	ist besser als die andere	o	ist besser für den job	o

1632	zwar noch nicht so viel Berufserfahrung, aber kann dazulernen	q	Fokus auf Erfahrungen und Abschluss, Rest nicht von so hoher Relevanz bei Buchhaltung	q	guter Abschluss aber wenig Berufserfahrung sieht danach aus, dass er gerade erst fertig geworden ist und nun erst in den Beruf einsteigt - noch lernfähig und formbar	q, a
1636	ausgewogenere Fähigkeiten	q	bessere Fähigkeiten	o	bessere Fähigkeiten	q
1644	höhere Softwareerfahrung	q	in mehreren Bereichen besser	q	Höherer Abschluss + Softwareerfahrung	q
1653	Allrounder	o	da allgemein sehr hohe Werte können soziale Skills vernachlässigt werden	q	insgesamt besser geeignet, auch wenn geringe Berufserfahrung	q
1660	Gleich viele Sterne gesamt, wie bisher bei allen Bewerber:innen. Softwareerfahrung ist größer als beim Mitbewerber, Berufserfahrung zwar geringer, aber sie ist auch jünger.	q, a	Zwar gleich viele Sterne, aber mehr Softwareerfahrung.	q	Nachdem ich nun einige Runden die sozialen Skills vernachlässigt habe, möchte ich auch eine:n Bewerber:in mit sehr guten Softskills im Verfahren. Insgesamt ist die q laut KI vergleichbar.	q
1672	besseres Gesamtpaket	o	deutlich besseres Gesamtpaket	q	höherer Abschluss, mehr Software erfahrung	q
1675	Hoher Abschluss, aber eine geringere Berufserfahrung. Hier ist bestimmt Potenzial.	q	Sehr breit aufgestellt. Vielseitig einsetzbar.	q	Kompetenzen sind ausbaufähig, aber nicht schlechter als vom Bewerber. Da in der vorherigen Auswahl von mir mehr männliche Bewerber ausgewählt wurden, wähle ich in diesem Fall die weibliche Bewerberin.	q, e, g
1685	Mehr Softwareerfahrung, Allg. bessere Balance der Skills	q	mehr Softwareerfahrung	q	Mehr Softwareerfahrung	q
1688	ebenfalls gute Abschlüsse und bessere Softwareerfahrung	q	viel Berufserfahrung und bessere softwareskills	q	hohe soziale Skills und mehr Berufserfahrung	q
1691	Bessere sozial skills	q	Ähnliche q	q	Mehr Softwareerfahrung	q
1718	ist qualifizierter für den Job	o	wirkt kompetenter als die andere	o	Der Kandidat ist besser	o
1745	Katharina Decker hat das ausgewogene Profil	q	Die Profile sind sich sehr ähnlich, aber das Profil von Karin Richter hat einen besseren Abschluss, während Arne Meyer bessere Sprachkenntnisse hat. Diese sind für die Stelle aber nicht von hoher Bedeutung	q	Sophia Ostermann hat mehr soziale Skills und Berufserfahrung	q
1746	same	x	Soziale Skills unwichtig, Rest dann 50:50	q	Soziale Skills unwichtig, Rest dann 50:50	q
1757	Ausgewogener und besserer Abschluss.	q	Skills sind ähnlich, bevorzuge die Frau	q, g	Mehr Berufserfahrung	q
1768	mehr Software Erfahrung - soziales nicht ganz so entscheidend	q	software erfahrung vor Soziale skills	q	kann sich sicherlich integrieren und so die sofware schwäche ausgleichen	o
1770	Schwierig-annähernd gleich. Abschluss und Sofwareerfahrung ausschlaggebend, Berufserfahrung kann noch kommen	q	schwierig-annähernd gleich. Softwareerfahrung und Abschluss ausschlaggebend	q	Soziale Skills deutlich vorn.	q
1780	mehr Softwareerfahrung	q	mehr Generalist als Karin	o	höhere Abschluss	q
1791	Soziale Skills, Softwareerfahrung kann man mit der Zeit aufbauen	q	soziale Skills sind wichtig - Frau Richter hat mehr zwischemenschliche Kompetenz	q	mehr Berufserfahrung	q
1806	overall besseres Rating bis auf Berufserfahrung	q	Fast gleiche Skills, Soziale Skills in dem Fall besser gewertet	q	mehr Berufserfahrung	q

1817	Berufserfahrung, soziale Skills und Softwareerfahrung sind höher!	q	Ich habe mich verklickt, ich wähle den 44-jährigen Bewerber aus Halberstadt aufgrund der höheren Softwareerfahrung aus, die in meinen Augen wichtiger sind als soziale Skills.	x	Berufserfahrung, soziale skills und Softwareerfahrung insgesamt höher ausgeprägt.	q
1823	ki	x	besserer Abschluss/ mehr Erfahrungen sprachen weniger wichtig bei Buchhaltung	q	besser Abschluss, softwareerfahrung	q
1828	Berufserfahrung, soziale Skills	q	Sprachen	q	soziale Skills deutlich besser	q
1830	Sprache Abschluss Software	q	Sprache, Software	q	Sprache Abschluss, Software	q
1850	Zwar etwas geringere Softwareerfahrung, aber mehr Berufserfahrung und hohe soziale Skills	q	bei gleicher Berufserfahrung etwas mehr Softwareerfahrung und sprachliche Skills	q	Zwar etwas weniger Softwareerfahrung, dafür aber deutlich mehr soziale Skills und mehr Berufserfahrung	q
1851	Besserer Abschluss und Sprachkenntnisse	q	Etwas mehr Erfahrung	q	Soziale Skills viel besser bewertet.	q

Table C3 Candidate Selection Reasons of Participants in Condition 3

ID	Reason 1	C	Reason 2	C	Reason 3	C
			**Condition 3 – Job 1**			
1341	besser in Programierung und mehr Berufserfahrung	q	Im Allgemeinen besser (mehr Sterne)	x	besser in programierung und sozilae skills	q
1342	Höhere Berufs- und Programmiererfahrung	q	Soziale Skills	q	Ausgeprägte soziale und programmierskills	q
1362	Dieser Bewerber hat die höhere Programmiererfahrung, die Fähigkeit, die ich bei dieser Stelle als am wichtigsten empfinde. Zusätzlich bringt er viel Berufserfahrung mit.	q	Die Bewerberin hat sehr viel Programmiererfahrung, was hoffentlich ihre mangelnde Berufserfahrung ausgleicht. Ihre sozialen Skills sind nicht so hoch, was aber auch bei dieser Stelle nicht die höchste Priorität hat.	q	Die Bewerberin hat sowohl Programmiererfahrung, als auch Berufserfahrung und außerdem hohe soziale Skills.	q
1383	mehr Berufserfahrung und mehr Programmiererfahrung	q	mehr Berufserfahrung und mehr soziale Skills	q	ausgewogeneres Verhältnis zw. Berufserfahrung und Programmiererfahrungen	q
1385	deutsch	r	deutsch	r	weiblich deutsch	r, g
1405	Ich würde keinen Türken einladen	r	Ich möchte einer Frau eine Chance geben und ich möchte keinen Türken einladen	r, g	Eine Frau ist mir lieber, einen Türken würde ich nicht einladen	r, g
1416	Bessere Programmiererfahrung P.S. Programmiererfahrung ist im Tool falsch geschrieben :)	q	Mehr Programmiererfahrung	q	Programmiererfahrung	q
1423	Mehr Erfahrung	q	Hat mehr Berufserfahrung und kann das Programmieren noch aufholen	q	Im Prinzip würde ich hier beide einladen, da sie sich nicht viel geben. Die Kandidatin hat einen etwas besseren Abschluss	q
1445	mehr Berufserfahrung	q	mehr Berufserfahrung und Soziale Skills	q	Berufserfahrung	q
1457	Mehr Erfahrung und Skills	q	Höhere Programmiererfahrung und Social Skills	q	Mehr Berufserfahrung	q
1474	Programmiererfahrung ist besser	q	Mehr Sterne bei Soft-Skills, bei Programmiererfahrung sieht das auch nicht schlecht aus	q	Mehr Berufserfahrung	q

1483	Beruf- & Programmiererfahrung	q	Programiererfahrung	q	Soziale Skills, Programiererfahrung	q
1508	Jünger	a	berufserfahren,	q	berufserfahren, jünger	q, a
1518	Kandidat 1 ist zu schlecht beim Programmieren.	q	Kandidatin 2 hat zwar mehr Programmierfahrung, kann aber nicht mit anderen Leuten umgehen. Deshalb trifft Kandidatin 1 eher die Anforderungen.	q	Beide Kandidaten sind geeignet, aber Kandidatin 2 trifft mehr dem gesuchten Profil zu.	q
1522	KI Empfehlung	k	Kandidatin sieht fähiger aus	o	Ist besser	o
1529	Sehr ähnlich wieder, Diversitätsgründe	q, e	Diversität	e	bisschen runderes Profil	o
1533	Mehr Berufs-/Programmiererfahrung.	q	Besserer Abschluss, mehr Programmiererfahrung.	q	Besserer Abschluss, mehrProgrammiererfahrung und nur ein Stern weniger bei Berufserfahrung, daher vermutlich vergleichbar.	q
1549	besser qualifiziert	q	besser qualifiziert	o	ausgewogeneres Profil	o
1552	In diesen Bereich ist Kommunikation und erforderliche berufliche Abschlüsse wichtig.	q	Hier würde bevorzugt Teamarbeit und soziales Verhalten vorteilhaft sein	q	Eine weibliche Person ist im Team kann das Gesamtklima auffrischen, Erfahrungen im Bereich Programmierung ist gut, beim Mitbewerber muss die Sprache verbessert werden, vielleicht im nächstem Jahr.	q, g
1554	Programiererfahrung wichtig und etwas höher	q	gibt sicher wenig Frauen in diesem Bereich und Sozialkompetenz ist auch wichtig	q, e, g	Berufserfahrung ist nicht so wichtig für mich, gute Punkte in den unteren Rubriken	q
1571	Berufserfahrung	q	soziale Skills	q	soziale Skills und Programmiererfahrung	q
1572	mehr Programmiererfahrung	q	Soziale Skills wichtig für Kontakt zu Belegschaft, restlichen Sterneunterschiede nicht zu signifikant	q	vergleichbar mit andere Bewerberin, mehr Programmiererfahrung	q
1602	mehr Programmiererfahrung	q	mehr Programmiererfahrung, besserer Abschluss	q	besserer Abschluss und mehr Berufserfahrung	q
1621	Hat einen besseren Abschluss und bessere Spracherfahrungen	q	bessere Sprach-und Programmerfahrungen	q	bessere Sprachkenntnisse	q
1631	Relevante Fähigkeiten besser, zudem KI-Empfehlung	q, k	Progammiererfahrung, Abschluss und Sprachkenntnisse gut,zudem KI-Empfehlung	q, k	Progammiererfahrung und Abschluss besser, zudem KI-Empfehlung	q, k
1640	Berufserfahrung und Diversity	q, e	soft skills	q	diversity	e
1646	Sehr Ausgewogen	o	gut in Soz. Slills und Programmieren, besser ausgewogen	q	Bessere Programiererfahrung	q
1657	bessere Berufserfahrung - bessere Programmiererfahrung	q	etwas schlechtere Programmier-, dafür bessere Berufserfahrung gleicht sich aus - deutlicher Vorteil bei Sozialen Skills, wichtig für Anwenderfragen	q	Sprache bei Kontrahent noch schlechter	q
1661	Hat mich mehr angesprochen	q	Besserer Abschluss, mehr Programmiererfahrung	q	KI Empfehlung	k
1662	KI und BE	q, k	BE und wiblich im Team IT schadet nicht	q, g	BE	q
1678	Mehr Programmiererfarung und Berufserfahrung.	q	Bessere soziale Skills und etwas mehr Berufserfahrung.	q	Mehr Programmiererfahrung.	q

1682	Der Bewerber hat mehr Berufserfahrung und bessere Programmierkenntnisse.	q	Die Bewerberin ist jünger, hat bessere Sprach- und Programmierkenntnisse. Als Einsteigerin hat sie noch weniger Berufserfahrung, das ist aber nicht schlimm.	q, a	Die Bewerberin hat besonders gute Social Skills und Programmiererfahrung.	q
1687	Kandidat hat mehr Berufs- und Programmiererfahrung.	q	Kandidatin hat mehr Berufserfahrung und deutlich bessere Soziale Skills. Fehlende Programmiererfahrungen könnten nachgeholt werden.	q	In den Punkten Programmiererfahrung und Abschluss besser. Sprache und Berufserfahrung kann im Laufe der Zeit schnell nachgeholt werden.	q
1696	geringere Sterne bei Sprachen als bei dem Konkurrzenzbewerber ist unter Umständen nicht schlimm, da Job keine großartigen Sprachkenntnisse verlangt - verlangt werden vielmehr Programmierkenntnisse und Berufserfahrung, diese sind bei Mustafa Özdemir höhe	q	Programmiererfahrungen scheinen sehr wichtig zu sein als IT-Administrator (vermute ich) - KI-Empfehlung	q, k	Unterschiede zwischen den Bewerbern sind nicht groß, maximal 1 Stern Abweichung je Kategorie (Sprachen, Abschluss usw..). Daher ist die Auswahl ohne Empfehlung recht schwierig. - KI-Empfehlun übernimmt hier die Entscheidung für mich	k
1710	schwierige entscheidung keine klare präferenz	q	soz skills	q	stärkeres gesamt profil	o
1720	Aufgrund der Programmier- und der Berufserfahrung	q	Programmierfahrung Sprachen	q	Solideres Gesampaket	o
1729	Empfehlung	k	Wirkt besser und qualifizierter	q	ist besser	o
1749	Bessere Berufs-und Programmiererfahrung. Interkulturell, daher Zugriff auf andere Resourcen	q, e, r	Bessere Kompetenzen, KI-Empfehlung	q, k	Bessere Programmiererfahrung und soziale Skills	q
1750	Mehr Programmier- und Berufserfahrung	q	Programmiererfahrung und Abschluss	q	Mehr Programmiererfahrung	q
1759	Berufserfarhung und Programmiererfahrung höher, bei gleichwertigen sozialen Skills	q	Indifferent; Verlass auf KI	k	höhere Programmiererfahrung bei gleichwertigen sozialen Skills	q
1771	Wegen der Berufserfahrung	q	Wegen der Programmierkenntnisse, Sprachen	q	Frauenquote, guter Durchschnitt	q, e, g
1787	besserer Abschluss	q	Sprachenskills könnten von Vorteil sein, mehr Programmiererfahrung	q	Mehr Programmiererfahrung und besserer Abschluss	q
1795	Sowohl mehr Programmier- als auch Berufserfahrung, was jobspezifisch wichtiger ist als die höhe des Abschlusses und die sprachlichen Skills!	q	Soziale skills sind weniger von Bedeutung, entscheidend ist die Programmiererfahrung. Deshalb vertraue ich hier der KI-Empfehlung.	q, k	In den für diesen Job wesentlichen Aspekten schneidet sie besser ab.	q
1801	Berufs- und Programmiererfharung	q	Bessere Mischung aus Skills	q	Sprachen	q
1810	Programmiererfahrung ist wichtiger als Sprachen zu können	q	Programmiererfahrugn wichtigster Skill	q	programmiererfahrung wichtiger als berufserfahrung	q
1811	mehr Sterne bei Programmiererfahrung und Berufserfahrung	q	mehr Programmierer.	q	mehr Berufserfahrung und mehr Sprachen	q
1824	höhere programmier Erfahrung	q	zwar weniger programmier Erfahrung jedoch höhere soziale skills und höhere Berufserfahrung	q	höhere Berufserfahrung und soziale skills	q
1832	Ich war nicht ganz sicher, welche qen in der IT-Administration wirklich gefragt sind und welche vernachlässigbar sind. Grundsätzlich erschien	q, k	Eine schwierige Entscheidung, da beide Schwächen aufweisen und die erste Kandidatin bei der Berufserfahrung überwiegt, die zweite	q	Eine schwer Entscheidun, aber letztlich bin ich der Empfehlung der KI gefolgt, da die sprachlichen Fähigkeiten bei beiden ausbaufähig sind	q, k

	mir die Berufserfahrung wichtiger als der Abschluss, und die Programmierfähigkeiten wichtiger als die Sprache, daher habe ich mich dem Urteil der KI angeschlossen. Ist die Gewichtung andersherum, muss das Urteil natürlich angepasst werden.		jedoch bei der Programmiererfahrung. Ich habe schließlich beschlossen, die Programmiererfahrung höher zu bewerten, aber war mir nicht ganz sicher.		und somit Abschluss und Programmiererfahrung für Kandidatin 1 sprechen, und nur die Berufserfahrung für Kandidatin 2.	
1834	Programmierung	q	Berufserfahrung und Programmierung	q	Berufserfahrung. und Programmierung	q
1836	mehr Berufserfahrung, bessere Programmierkenntnisse	q	mehr Berufserfahrung und viel mehr soziale Skills	q	mehr Berufserfahrung und sonst relativ ähnlich	q
1857	Für die Rolle ist Berufserfahrung und Programmiererfahrung wichtiger	q	fehlende Berufserfahrung könnte durch bessere Ausbildung kompensiert werden, Soziale skills sind auch wichtig - könnten bei der Rolle auch kontraproduktiv sein	q	in etwa gleichwertig, entscheidend ist die etwas höhere Berufserfahrung	q
			**Condition 3 – Job 2**			
1341	Passt besser ins anforderungsprofil	q	mehr soziale skills und deutlich jünger	q	passt besser ins anforderungsprofil	q
1342	Soziale Skills besser - Interessenkonflikte ect.	q	Ausgewogenere Fähigkeiten	o	Wichtigsten Skills für den Beruf vorhanden	q
1362	Der Bewerber ist im den Bereichen Soziale Skills und Methodenkompetenz sehr gut und gut ausgebildet, zusätzlich bringt er viel Berufserfahrung mit.	q	Die Bewerberin ist im Bereich Methodenkompetenz gut ausgebildet. Ihre sozialen Skills sind nicht so hoch, allerdings kann sie das ggf. mit ihrer hohen Berufserfahrung ausgleichen. Zusätzlich hat sie auch einen guten Abschluss.	q	Der Bewerber ist im Bereich Methodenkompetenz sehr gut ausgebildet, seine sozialen Skills sind noch ausbaufähig, aber er konnte bereits Berufserfahrung sammeln.	q
1383	mehr Berufserfahrung und höhere soziale Skills	q	mehr Methodenkompetenz und mehr Berufserfahrung	q	mehr Berufserfahrung und mehr Methodenkompetenz	q
1385	jünger	a	berufserf	q	Kompetent	o
1405	Thomas ist zu alt	a	Monika ist zu alt	a	Patrick ist prompt einsetzbar	o
1416	Methodenkompetenz und Social Skills besser	q	Methodenkompetenz besser	q	Methodenkompetenz besser	q
1423	Die mangelnde Methodenkompetenz kann er sich sicher schnell aneignen, soziale Skills zu lernen ist ggf schwieriger	q	Einfach erfahrener als andere Kandidatin. Trotz geringeren sozialen Skills, wäre wohl diese Person besser	q	Die schwierigste Wahl, da Kandidat alt. Aber der andere ist auch nicht optimal. Evtl gibt es einen besseren Kandidaten, wenn Patrick in Rente geht oder nach der Probezeit	q, a
1445	Berufserfahrung, Soziale Skills	q	Berufserfahrung, Methodenkompetenz	q	Methodenkompetenz	q
1457	mehr Erfahrung	q	mehr Erfahrung und Kompetenz	q	Ausgeglicheneres Bild	q
1474	Berufserfahrung und Soziale Skills sind besser	q	Abschluss, Berufserfahrung und Soziale Skills sind auf jeden Fall besser	q	Soziale Skills sind sehr wichtig, daher die Person	q
1483	Soziale Skills, berufserfahrung	q	Bessere Werte	o	ausgeglichenere Werte	o
1508	Methodenkompetenz, jünger	q, a	Methodenk.	q	Methodenkom.	q
1518	Kandidat 2 besitzt ein kompletteres Profil	o	Kandidatin 2 besitzt ein kompletteres Skillset.	q	Kandidat 1 besitzt keine Methodenkompetenz, weshalb er für den Job nicht geeignet ist.	q
1522	KI Empfehlung	k	Subjektiv besser	o	Sieht geeigneter aus als der andere	o
1529	sind sehr ähnlich, ich vertraue auf KI	k	Sehr ähnlich wieder, vertraue auf KI	k	weniger starke schwächen	o
1533	Sieht insgesamt sehr gut aus.	o	Mehr Berufserfahrung, mehr Methodenkompetenz.	q	Mehr Berufserfahrung, mehr Methodenkompetenz.	q

1549	soz skills besser	q	soz skills besser	q	soz skills besser	q
1552	Knappe Entscheidung durch Berufserfahrung.	q	Wieder Knappe Entscheidung, soziale Kompetenz und Abschluss ausschlaggebend.	q	Entscheidend war hier die Methodenkompetenz.	q
1554	Ist zwar älter aber hat gute soziale Skills und Berufserfahrung	q, a	Insgesamt besser aufgestellt, leider nicht so gute Soziale Skills aber würd ich gerne kennenlernen	q	Sprachen und Soziale Skills, kann gut für Auslandsgespräche/Planungen eingesetzt werden, Methoden müssten gestärkt werden	q
1571	Berufserfahrung und Methodenkompetenz	q	Soziale Skills	q	Soziale Skills	q
1572	Mehr Soziale Skills und Berufserfahrung	q	höhere Soziale Skills, ausgewogeneres Bild	q	bessere Methodenkompetenz, mehr Erfahrung	q
1602	Erfahrung gleicht schlechteren Abschluss aus bzw. lässt diese Person attraktiver wirken	q	Fähigkeiten ausgewogener verteilt, könnte hier von Vorteil sein	q	überzeugt durch Methodenkompetenz und Berufserfahrung	q
1621	besserer Abschluss und sklills	q	nur einmal zwei sterne	q	nirgendwo einen stern	q
1631	Bewerber überzeugt durch bessere Social Skills und mehr Berufserfahrung, zudem KI-Empfehlung	q	Entscheidung letztlich aufgrund der Methodenkompetenz und der Empfehlung der KI getroffen	k	Gute Methodenkompetenz und Berufserfahrung, zudem KI-Empfehlung	q, k
1640	Abschluss zweitrangig	q	soziale skills	q	1 Stern bei Methodenkompetenz ist zu wenig	q
1646	Erfahrung und soziale Skills schlagen hier Methodenkomprtenz	q	Berufserfahrung, Methodenkompetenz und Abschluss wiegen schwächen in sozialen Skills auf	q	Methodenkompetenz von 1em Stern bei anderem Kandidaten inakzeptabel	q
1657	Summe aus Sozialen skills und Methodenkompetenz hier größer - Minimum aus Sozialen skills und Methodenkompetenz hier größer	q	bessere Methodenkompetenz, Sprache und Berufserfahrung gleichen die schlechteren Sozialen Skills aus. Schlechterer Abschluss in Anbetracht der Berufserfahrung irrelevant (Abschluss ist mit 4/5 nach wie vor ausreichend!!)	q	- Kontrahent mit 1/5 bei Methodenkompetenz ungeeignet, auch bessere Soziale Skills des Kontrahenten gleichen das nicht aus	q
1661	KI Empfehlung	k	Sehe hier das größere Potenzial	o	KI Empfehlung	k
1662	Berufserfahrung, Methodenkomp. und KI	q, k	Soziale Skills	q	KI und Sprachen hier nicht so wichtig	q, k
1678	Bessere soziale Skills.	q	Mehr Berufserfahrung.	q	Bessere Methodenkompetenz.	q
1682	Sehr gute Soziale Skills und viel Berufserfahrung.	q	Solidere qen.	o	Bessere Medienkompetenz, nur eine q unter drei Sternen	q
1687	Entscheidend hier die Kombination aus Sozialen Skills und Berufserfahrung. Methodenkompetenz ist leicht schlechter, kann aber vermutlich noch durch Schulungen verbessert werden.	q	Auch hier ist die soziale Kompetenz entscheidend. Da die Bewerberin jünger ist, kann folglich weniger Berufserfahrung aufgezeigt werden. Die ebenfalls geringere Methodenkompetenz kann vermutlich schnell verbessert werden, da der ausgezeichnete Abschluss aufzeigt, dass die Person ehrgeizig ist.	q	Entscheidend hier ist die sehr gute soziale Kompetenz. Fehlende Berufserfahrung und geringe Methodenkompetenz könnte darauf zurück zuschließen sein, dass die Frau einige Jahre aufgrund von Schwangerschaften nicht gearbeitet hat. Dadurch sollte die Kandidatin nicht benachteiligt werden.	q, g, e
1696	Vertrauen in die KI-Empfehlung, da Entscheidung ansonsten schwierig, da beide Bewerber dieselbe Anzahl an Sternen haben	k	KI-Empfehlung	k	Vertrauen in die KI	k
1710	soziale skills und erfahrung	q	soziale skills und alter	q	methodenkompetenz bei konkurenz zu schwach	q
1720	Jünger und besser ausgebildet	q, a	Methodik - Berufserfahrung	q	Ausgegliuchner	o
1729	Empfehlung	k	Ist besser	o	ist besser	o

1749	Mehr Berufserfahrung und soziale Skills	q	KI-Empfehlung	k	Bessere soziale Skills	q
1750	Viel Berufserfahrung und soziale Skills	q	Sprachlich besser + Medienkompetenz	q	mehr Berufserfahrung und bessere Medienkompetenz	q
1759	Kombination aus Berufserfahrung und Soziale Skills	q	ggf. noch lernföhiger bzgl. Methodenkompetenz aufgrund Alter	q, a	Methodenkompetenz beim Vergleichsbewerber zu gering	q
1771	Jung	a	Jungen Menschen eine Chance geben,	e, a	Bessere Bewertungen	q
1787	Mehr soziale Skills und Berufserfahrung	q	deutlich jünger und nur etwas weniger Berufserfahrung und Medienkompetenz	q, a	Mehr soziale Skills und gut in Sprachen	q
1795	In Gedanken an vorherige eingeladene Bewerber*innen, passt Sven aufgrund seines jungen Alters vermutlich besser ins Team, was die niedrige Bewertung der sozialen Skills ausgleichen könnte. Ausschlaggebend für mich ist letzten Endes aber der höhere Abschluss und die besseren Methodenkompetenzen!	q, a	Hier würde ich mich auf die KI-Empfehlung berufen. Entsprechend der Bewertung sehe ich in Monika einen Allrounder, der den Job trotz geringerer sozialer Skills gut ausüben kann, sodass man über die ein oder andere Schwäche auch hinweg sehen kann.	q, k	Die Methodenkompetenz überzeugt mich hier.	q
1801	Bessere Mischung als anderer Kandidat	o	Bessere Skillmischung	q	Methodenkompetenz	q
1810	höhere soziale Skills = wichtig für PRM bei Abstimmung	q	besseres gesamtpaket dank medienkompetenz + berufserfahrung	q	IT Job benötigt Mindestmaß an Medienkompetenz	q
1811	mehr Berufserfahrung	q	viel jünger	a	Methodenkompetenz und Berufserfahrung mehr	q
1824	ausgeglicheners Profil	q	höhere methodenkompetzenzen	q	höhere methodnekompetenzen	q
1832	In den wichtigen Kategorien überlegen, Kandidat 2stolpert über die mangelnde Soziale Kompetenz.	q	19 Jahre Jünger, deutlich überlegen im sozialen Bereich + sehr hoher Abschluss, der nahelegt, dass sowohl Berufserfahrung als auch Methodenkompetenz schnell ausgeglichen werden können.	q, a	Beide keine gute Wahl, da eklatante Schwächen in wichtigen Bereichen (Kandidat 1 Sozial, Kandidat 2 Kompetenz). Kandidat 1 bietet das insgesamt ausgeglichenere Gesamtpaket	q
1834	Berufserfahrungm Soz. Skills & Methoden	q	Soz. Skills	q	Berufserfahrung, Methoden	q
1836	Soziale Skills sind wichtiger als Abschluss	q	ausgeglichenere SKills	q	ausgeglichenere Skills	q
1857	vergleichbar, aber Berufserfahrung und Sozial skills besser	q	vergleichbar, soziale skills höher (Verantwortung)	q	etwas mehr Erfahrung	q
			**Condition 3 – Job 3**			
1341	mehrSoftwareerfahrung	q	mehr Softwareerfahrung	q	mehr Softwareerfahrung	q
1342	Abschluss und Sprache für qualitativ und sprachlich gute Abschlüsse	q	Ausgewogenere Fähigkeiten	q	Schwere Entscheidung, folge KI	k
1362	Die Bewerberin hat bereits einiges an Softwareerfahrung und einen sehr guten Abschluss. Sie konnte ein bisschen Berufserfahrung sammeln und hat zusätzliche gute soziale Skills.	q	Die Bewerberin hat sowohl Softwareerfahrung als auch viel Berufserfahrung. Zusätzlich hat sie einen sehr guten Abschluss.	q	Der Bewerber konnte noch keine Berufserfahrung sammeln, aber er hat einiges an Softwareerfahrung und einen sehr guten Abschluss.	q
1383	mehr Berufserfahrung höhere soziale Skills	q	höhere soziale Skills, stelle ich mir angenehmer im Team vor	q	mehr Berufserfahrung und höhere soziale Skills	q
1385	weiblich	g	sprachen	q	berufserfahrung	q

1405	Mädel sind gute Buchhalterinnen	o	mehr Softwarskills	q	die Frau ist arbeitsaffiner	o
1416	Mehr Berufserfahrung	q	Mehr Berufserfahrung und Softwareerfahrung	q	Mehr Berufserfahrung	q
1423	Knappe Entscheidung, hier würde ich auch beide einladen und dann entscheiden. Aber gehe mit der Entscheidung der KI	k	Mehr Softwareerfahrung, sonst sehr ähnlich	q	Bessere soziale Skills, nur geriner Unterschied bei Softwareerfahrung	q
1445	Berufserfahrung	q	Abschluss, Berufserfahrung, Soziale Skills	q	Berufserfahrung	q
1457	Mehr Erfahrung, höherer Abschluss	q	Mehr Erfahrung	q	ausgeglichenere Verteilung	o
1474	Mehr Sterne bei Soft-Skills, Berufserfahrung . Sprachkenntnisse sind bei der Aufgabe nicht so wichtig	q	Softwareerfahrung, Abschluss ist besser	q	Berufserfahrung und Soft-Skills sind auf jeden Fall besser	q
1483	Unsicher, KI Empfehlung genommen	k	Ausgeglichene Werte	o	Soziale Skills, Berufserfahrung	q
1508	weiblich, jünger	g, a	weiblich	g	berufserf.	q
1518	Kandidatin 2 hat für den Beruf bessere Fähigkeiten. Kandidat Nummer 1 hat zwar herausragende Soziale Skills, diese werden aber nicht gebraucht.	q	Kandidat 2 kann eher mit einer benötigten Software umgehen, während die Sozialen Skils bei dem gefragten Job nicht so wichtig sind. Der Abschluss bei dem Alter der Kandidaten nicht mehr so wichtig.	q	Kandidatin 2 weist mehr Berufserfahrung auf und ist Dank ihrer sozialen Skills besser in der Lage mit anderen Leuten zu arbeiten.	q
1522	Sieht subjektiv besser aus	o	wirkt besser	o	Gesamtpaket passt besser	o
1529	Hat zwar nicht so viel Erfahrung, hat aber ansonsten ein gut abgerundetes Profil	q	Sind ähnlich qualifiziert, in dem Fall würde ich eine Frau bevorzugen	q, g	Auch hier ähnlich, weiblich bevorzugt	q, g
1533	Besserer Abschluss, bessere Softwareerfahrung.	q	Guter Abschluss, viel Berufserfahrung.	q	Besserer Abschluss, mehr Softwareerfahrung.	q
1549	Bessere soziale Skills	q	Ausgewogeneres Profil	o	Soz Skills	q
1552	Enge Auswahl, der KI Empfehlung gefolgt, Anderer Bewerber eventuell auf Nachrücker setzen.	k	Knappes Rennen, Frau Richter konnte überzeugen.	o	Der Abschluss hat die geringe Berufserfahrung ausgeglichen.	q
1554	soziale Skills sind ausgesprochen hoch, andere Werte auch in Ordnung	q	mehr Soziale Kompetenz und ansonsten auch etwas stärker	q	schwer Entscheidung, hab mich wegen der Sozialen Skills und des Geschlechts für diese Bewerberin entschieden	q, g
1571	soziale Skills und Berufserfahrung	q	Softwareerfahrung und Berufserfahrunf	q	soziale Skills	q
1572	Mehr Softwareerfahrung und besserer Abschluss, Soziale Skills weniger wichtig, bessere Sprachkenntnisse sind nützlicher für Berichte schreiben	q	Mehr Softwareerfahrung, Sterneverteilung gibt für mich ein ähnliches Bild der Fähigkeiten, Soziale Skills weniger wichtig	q	Mehr Berufserfahrung, Abstände in anderen Kategorien nicht dramatisch	q
1602	Gesamtpaket wirkt stimmiger - Erfahrungen können schnell gesammelt werden	q	überzeugt durch besseren Abschluss - Ansonsten gleichwertig empfunden	q	besserer Abschluss - Berufserfahrung kann schnell gesammelt werden	q
1621	bessere sprachkenntnisse und softwareerfahrung	q	mehr sterne	q	zwar wenig berufserfarunge, aber bessere softwareerfahrung	q
1631	Aufgrund der KI Empfehlung und da Sprache, Abschluss und Softwareerfahrung höher waren	q, k	Berufserfahrung, Sprachkenntnisse und Softwareerfahrung höher	q	Bewerber in allen Punkten, außer der Berufserfahrung und den Social Skills besser	q
1640	Berufserfahrung und soziale skills wichtiger als Abschluss	q	ähnlich qualifiziert, aber diversity	q, e	Berufserfahrung und soziale skills wichtiger als Abschluss	q

1646	Ausgewogener Abschluss + Berufserfahrung Software meist leicht erlernbar	q	Ausgeglichener in relevanten Gebieten (Bildung, Erfahrung, Software	q	Ausgeglichener in relvanten Kategorien	o
1657	besserer Abschluss gleicht schlechtere Berufserfahrung aus - Softwareerfahrung etwas besser	q	- schlechtere Soziale Skills irrelevant (2/5 reicht für Buchhaltung aus) - bessere Softwareerfahrung mit Blick auf Zukunft relevanter als schlechterer Abschluss	q	nur 1/5 Berufserfahrung beim Kontrahent ist zu wenig	q
1661	Die Auswahl ist mir schwer gefallen, ich habe mich auf die Empfehlung verlassen	q	Die einzelnen Kriterien sind ausgewogener erfüllt	q	Die Auswahl ist mir schwer gefallen, ich habe mich auf die Empfehlung verlassen	k
1662	KI und Abschluß	q, k	KI Abschluß und Berufserfahrung	q, k	Abschluss	q
1678	Mehr Berufserfahrung und soziale Skills.	q	Besserer Abschluss und Berufserfahrung.	q	Bessere soziale Skills.	q
1682	Weiblich, jünger, nur eine q unter drei Sterne.	q, g, a	Dreimal vier Sterne. Social Skills nicht ganz so wichtig in Buchhaltung	q	Berufseinsteiger mit sehr guten Abschluss	q
1687	Softwareerfahrung entscheidend.	q	In 3 von 5 Punkten besser. Soziale Kompetenz ist in der Buchhaltung zweitrangig. Der Abschluss ist mit weniger Sternen bewertet, ist nach jahrelanger Berufserfahrung jedoch nicht stark relevant.	q	Softwareerfahrung entscheidend. Berufserfahrung ist gering weniger als bei der anderen Kandidatin, soziale Kompetenz ist in der Buchhaltung zweitrangig.	q
1696	Vertrauen in die KI	k	beide Bewerber gleiche Sternanzahl, daher Vertrauen in KI	k	Vertrauen in die KI	k
1710	erfahrung	q	softwareerfahrung	q	kompetenz und abschluss	q
1720	Solideres GEsamtprofil	q	Mehr Erfahrung und besser social skills	q	Mehr Erfahrung und bessere Soccial Skills	q
1729	Empfehlung	q	empfehung	k	Empfehlung, Kandidaten sind sich ähnlich	k
1749	Besseren Abschluss und mehr Softwareerfahrung	q	Wirkt insgesamt kompetenter	q	KI-Empfehlung	k
1750	Besserer Abschluss und mehr Softwareerfahrung	q	bessere Softwareerfahrung	q	Besserer Abschluss und mehr Softwareerfahrung	q
1759	Höhere Berufserfahrung	q	KI-Empfehlung; Andere Eigenschaften fast gleichwertig	k	KI-Empfehlung, da Bewertungen fast gleichwertig	k
1771	Frauenquote, bessere Beurteilungen	q, e, g	Softwareerfahrung, bessere Beurteilungen	q	Frauenquote,	e, g
1787	höherer Abschluss, mehr Softwareerfahrung	q	Sprachen nicht so wichtig, Abschluss besser	q	Besserer Abschluss und mehr Softwareerfahrung	q
1795	hier vertraue ich der KI-Empfehlung!	k	Geringerer Abschluss und hohe Software-Erfahrung!	q	Entspricht dem Anforderungsprofil besser als Dominik.	q
1801	soz Skills	q	Bessere Kombo	o	soz Skills und Berufserfahrung	q
1810	Soziale Skills unwichtig für den Job	q	verklickt	x	besserer abschluss + softwareerfahrung	q
1811	da soziale skills nicht so entscheidend	q	mehr Softwarerfahrung	q	mehr Softwareer.+Berufserfahrung	q
1824	ausgeglicheners Profil	o	ausgeglicheneres Profil	o	ausgeglicheneres Profil	o
1832	Größter Vorteil von Kandidat 1 war in den weniger wichtigen Sozialen Skills, dafür Kandidatin 2 bei Abschluss und Sprachen überlegen.	q	Abschluss wichtiger als Sprachen, Soziale Skills unwichtiger als Berufserfahrung. Harte Ent-	q, a	Im Vergleich zu anderen Kandidaten würde ich keinen von beiden einladen. Da beide chancenlos sind, habe ich keine große Energie investiert und bin dem Rat der KI gefolgt.	k

	Berufs- und Software-Erfahrung heben sich gegenseitig auf, daher Wahl von Kandidatin zwei		scheidung, aber Kandidat 2 ist zwei Jahre jünger und kann in dieser Zeit ggf die Berufserfahrung aufholen.			
1834	Mehr Berufserfahrung	q	Mehr Softwareerfahrung	q	Berufserfahrung	q
1836	mehr Berufserfahrung und soziale Skills	q	softwareerfahrung ist wichtiger	q	mehr Berufserfahrung	q
1857	verantwortungsvoller Job, höhere Sozialkompetenz und Berufserfahrung	q	vergleichbare Profile, höhere Soziale skills	q	vergleichbar, mehr soziale Skills, etwas mehr Berufserfahrung	q

Table C4 Candidate Selection Reasons of Participants in Condition 4

ID	Reason 1	C	Reason 2	C	Reason 3	C
			**Condition 4 – Job 1**			
1336	Berufserfahrung und Programmiererfahrung im IT Bereich wichtig	q	Fast gleichwertig, aber soziale Skills bei gleichwertiger Erfahrungsstufe höher	q	Programmiererfahrung	q
1368	programmierung	q	programmierung, sprachen	q	insgesamt besser	o
1372	Merh Programmiererfahrung und mehr Berufserfahrung und gute soziale Skills plus Abschluss	q	Mehr an Berufserfahrung wiegt die Programmiererfahrung auf, soziale Skills sehr viel besser	q	Finde Berufserfahrung wichtiger als ein Stern mehr bei Programmiererfahrung. Soziale Skills bei beiden gleich.	q
1395	Berufserfahrung, Programmieren	q	Programmieren	q	Programmieren	q
1396	Bessere Bewertungen in den relevanten Punkten	q	Mehr Berufserfahrung und ausreichende Programmiererfahrung	q	Mehr Programmiererfahrung	q
1413	mehr Berufs- und Programmiererfahrung	q	besserer Abschluss und Programmiererfahrung, soziale Skills eher geringe Priorität	q	besserer Abschluss und Programmiererfahrung	q
1420	Kandidat ist besser	o	KI Empfehlung	k	Kandidatin scheint besser zu sein	o
1440	Empfehlung der KI, mehr Erfahrung	q, k	KI-Empfehlung. zwar weniger Berufserfahrung aber deutlich mehr Programiererfahrung	q, k	KI-Empfehlung, mehr Sterne	k
1459	Erfahrung als Admin wichtiger	q	Allgemeines	o	Erfahrungswerte balancierter	o
1475	Programmiererfahrung	q	Soziale Skills	q	Sprachen	q
1502	mehr Berufs- und Programmiererfahrung	q	Besserer Abschluss, mehr Programmiererfahrung	q	Besserer Abschluss, mehr Programmiererfahrung, gute soziale Skills	q
1504	Mehr Erfahrung	q	Höhere soziale Skills	q	Bessere Sprachkenntnis	q
1512	Hat mehr Berufserfahrung und Programmiererfahrung Gleiche Social Skills	q	Schwierige Entscheidung, habe mich der Empfehlung der KI angeschlossen	k	KI Empfehlung	k
1523	bessere Programmiererfahrung	q	bessere Soziale skills	q	Sprach-fähigkeiten besser, soziale Skills gut-sehr gut	q
1531	mehr Berufs- und Programmiererfahrung	q	Berufserfahrung und Social Skills	q	Programmiererfahrung	q
1538	Höhere Berufs- und Programmiererfahrung, Sprache eher weniger wichtig, da Kommunikation nur zweitrangig ist.	q	Besserer Abschluss und mehr Programmiererfahrung. Berufserfahrung gering, kann aber angelernt werden. Social skills bei dieser Stelle weiniger wichtig und daher ausreichend.	q	Höhere Programmiererfahrung und besserer Abschluss. Insgesamt nur ein kleiner Vorsprung zu Mitbewerberin. Sprache als eher wenig wichtig angesehen.	q

1542	für IT-JOb sind Erfahrung, Programmier-Skills wichtiger als Sprache (eh meist auf englisch) und abschluss	q	für IT-JOb wieder programmiererfahrung sehr wichtig plus alle kategorien bis auf berufserfahrung besser als bei anderen bewerberin	q	(abschluss), programmiererfahrung besser, wenn auch berufserfahrung geringer	q
1558	genug Programmier-/Berufserfahrung	q	bessere soziale Skills (hier wichtig, weil Unterstützung Belegschaft), Programmiererfahrung ähnlich	q	wichtige Skills erfüllt	o
1565	Berufserfahrung	q	Frauenquote erhöhen	e, g	Programmiererfahrung	q
1570	Empfehlung KI weil Rest etwa gleichwertig	k	Empfehlung KI, Rest etwa gleich	q, k	Etwa gleich, deshalb Empfehlung KI	q, k
1581	mehr Berufs- und Programmiererfahrung	q	mehr Programmiererfahrung, besserer Abschluss	q	mehr Programmiererfahrung	q
1588	Mehr Berufserfahrung und höhere Programmerfahrung	q	weit höhere Soziale Skills	q	mehr Programmiererfahrung	q
1617	berufs- und programmiererfahrung besser	q	bessere soziale skills	q	programmiererfahrung und abschluss besser	q
1619	mehr Berufserfahrung - mehr Programmiererfahrung	q	mehr Sprachen - höherer Abschluss - mehr Programmiererfahrungen - KI-Empfehlung	q, k	mehr Sprachen - mehr Berufserfahrung - mehr soziale skills	q
1627	Erfahrung in jeglicher Hinsicht ist key	o	Knapp besser wegen Abschluss	q	Besserer Abschluss	q
1641	Berufserfahrung, programmiererfahrung	q	auf den meisten Kriterien besser	q	abschluss, programmiern	q
1642	mehr Programmiererfahrung	q	ausgeprägtere Programmierkenntnisse	q	mehr Programmierkenntnisse	q
1652	Hat mehr Arbeitserfahrung und Programmerfahrung, wobei der andere Teilnehmer nur einen besseren Abschluss hat und leicht besser mit Sprachen ist	q	Bessere Attribute	o	Prima attribute, viel programmiererfahrung und soziale skills, wird auch von der KI empfohlen	q, k
1663	mehr berufserfahrung und soziale Fähigkeiten	q	mehr berufs- und programmiererfahrung + KI empfehlung	q, k	Ki empfehlung+ mehr Programmiererfahrung und besserer Abschluss	q, k
1681	Die Programmierfähigkeit und die Berufserfahrung sind höher	q	Die Berufserfahrung ist höher, außerdem sind die sozialen Fähigkeiten besser ausgeprägt	q	Die Programmiererfahrung ist noch besser	q
1684	mehr Berufs- & Programmiererfahrung	q	soziale Skills und Berufserfahrung höher	q	Programmiererfahrung m.E. relevanter als Berufserfahrung; aber tendenziell beide einladen	q
1690	Kompetenter	o	Jünger, bei ungefähr gleicher Kompetenz	q, a	Kompetenter trotz geringerer Berufserfahrung	q
1694	Programmierung	q	Programmiererfahrung -Sprachen	q	bessere Programmiererfahrung	q
1711	Programmierfähigkeit und Berufserfahrung entscheidend	q	Abschluss und Kompetenz entscheidend in IT	q	besser als Mitbewerber	o
1717	mehr erfahrungen	q	mehr berufserfahrung, bessere soziale skills für fragen der belegschaft	q	bessere bewertung	o
1726	Programmierung	q	Programmierung	q	Programmierung	q
1748	Berufserfahrung	q	Programmiererfahrung	q	Programmiererfahrung	q
1755	Bewerber hat mehr Berufs- und Programmiererfahrung	q	Guter Abschluss und viel Programmiererfahrung macht die fehlende Berufserfahrung wett. Soziale Skills für IT-Administration in meinen Augen nicht so wichtig	q	nur minimal nachteilig in Abschluss und Programmiererfahrung, dafür bessere Berufserfahrung und höhere Sprachkenntnis	q

1769	mehr Erfahrung in Programmierung und Beruf - höhere Relevanz als Noten	q	bessere soziale skills	q	Besserer Abschluss in Kombination mit mehr Programmiererfahrung und gleichzeitig gute soz. Kompetenz	q
1772	IT- Kenntnisse	q	IT- Kenntnisse	q	IT- Kenntnisse	q
1785	Gleich viele Sterne, ähnlich alt aber von Ki empfohlen	q, k	Gleich viele Sterne und das Alter ista uch ähnlich, aber von Ki empfohlen	q, k	Gleich viele Sterne wir die andere, wobei nur schwächen in der Sprache, das für den Job jedoch nicht so wichtig ist. Auch von der KI empfohlen	q, k
1794	Soziale Skills (Anwenderunterstützung) und Programmiererfahrung (Problemlösung) besser	q	Soziale Skills und mehr Berufserfahrung, Programmiererfahrung kann Herrn Sahin nicht retten	q	mehr Programmiererfahrung	q
1805	ist geeigneter für die Stelle	o	ist besser	o	Empfehlung	k
1821	KI	k	sind ähnlich, daher KI Empfehlung	k	KI	k
1822	KI	k	Ki	k	KI	k
1837	Bessere Berufserfahrung und Programmiererfahrung	q	bessere soziale Skills	q	bessere Programmiererfahrung, soziale Skills, Abschluss	q
1844	Mehr Programmiererfahrung	q	Mehr Programmiererfahrung	q	Mehr Programmiererfahrungen	q
1852	etwas mehr Erfahrung, KI-Empfehlung	q, k	mehr Programmiererfahrung, KI-Empfehlung	q, k	mehr Programmiererfahrung, KI-Empfehlung	q, k
1855	Programmiererfahrung und Berufserfahrung besser	q	Programmiererfahrung besser	q	Mehr Berufserfahrung	q
			**Condition 4 – Job 2**			
1336	Berufserfahrung und Soziale Skills bei durchschnittlicher Methodenkompetenz + KI-Empfehlung	q, k	Berufserfahrung + Methodenkompetenz	q	KI-Empfehlung	k
1368	berufserfahrung	q	berufserfahrung	q	well rounded	o
1372	Bessere Methodenkompetenz, und Jünger, soziale Skills bei dem Job nicht vorrangig	q, a	Besseres Gesamtpaket, Methodenkompetenz und mehr Berufserfahrung. soz. Skills hier nicht ganz so relevant.	q	Berufserfahrung und Methodenkompetenz hier besser, was für diesen Job relevant ist.	q
1395	Methoden, weniger kurz vor de Rente	q	Methoden, Erfahrung,	q	Erfahrung, Methoden	q
1396	Herr Ackermann ist zu alt	a	Ist deutlich jünger und besitzt eine identische Gesamtbewertung	q, a	Mehr Methodenkompetenz	q
1413	mehr Berufserfahrung und soziale Skills	q	besserer Abschluss und soziale Skills	q	insgesamt ausgewogenere Verteilung der Fähigkeiten, anderer Bewerber hat zu wenig insgesamt	q
1420	Kandidaten scheinen ähnlich zu sein, deshalb KI Empfehlung	k	Kandidatin wirkt besser	o	Der andere Kandidat ist zu schlecht (1 Stern bei Methodenkompetenz ist zu wenig)	q
1440	KI-Empfehlung	k	KI-Empfehlung	k	KI-Empfehlung	k
1459	allg. besser	o	Ausgeglichener	q	Methoden > Sprachen	q
1475	Berufserfahrung	q	Alter	a	Berufserfahrung & Methoden	q
1502	Er bringt mehr Erfahrung und soziale Skills mit.	q	Sie hat den besseren Abschluss, mehr soziale Skills und mehr Methodenkompetenz	q	lieber weniger Sprachen und mehr Methodenkompetenz und Erfahrung.	q
1504	Höhere soziale skills	q	höhere methodenkompetenz und erfahurng	q	Mehr erfahrung	q

1512	KI Empfehlung	k	KI Empfehlung	k	Überall eher gut	q
1523	bessere soziale Skills	q	besseres Gesamtpaket	o	nur bessere Methodenkompetenz ansonsten durchschnitt	q
1531	Soziale Skills + Berufserfahrung	q	Methodenkompetenz + Berufserfahrung	q	Methodenkompetenz	q
1538	Social skills und Berufserfahrung vielversprechend, Methodenkompetenz nur leicht geringer als beim Mitbewerber.	q	Sehr knapper Vorsprung. Methodenkompetenz und Berufserfahrung überzeugen, geringere social skills allerdings nicht irrelevant.	q	Methodenkompetenz kritisch für diese Stelle, daher schneller Ausschluss des anderen Bewerbers.	q
1542	Medienkompetenz ist da	q	jünger --> medienkompetenz kann hier noch besser aufgebaut werden als soziale skills beim gegenüber vermutlich	q, a	zu wenig medienkompetenz bei anderem bewerber	q
1558	mehr Berufserfahrung, KI-Empfehlung	q	Methodenkompetenz, Berufserfahrung	q	Viel bessere Methodenkompetenz (wichtig)	q
1565	Berufserfahrung	q	Berufserfahrung	q	Sprachen	q
1570	Relativ gleich, Empfehlung der KI gefolgt	k	Soziale Skills wichtiger als manch andere Dinge	q	Empfehlung der KI	k
1581	soz- skills und Berufserfahrung besser	q	soz. skills besser	q	Methodenkompetenz besser	q
1588	gute Methodenkompetenz und soziale Skills	q	Bessere Methodenkompetenz und mehr Berufserfahrung	q	sehr gute Methodenkompetenz	q
1617	berufserfahrung besser	q	methodenkopmpetenz und berufserfahrung besser	q	bessere Methodenkompetenz	q
1619	mehr Berufserfahrung - mehr soziale skills - nur geringfügig weniger Medienkompetenz	q	mehr Sprachen - mehr Berufserfahrung - mehr Medienkompetenz	q	mehr Berufserfahrung - deutlich mehr Medienkompetenz	q
1627	Leute aus Tübingen sind super!	o	Soziale Skills sind sehr wichtig	q	Jünger	a
1641	höhere soziale Skills, ansonsten sind die sich ziemlichähnlich	q	soziale skills	q	Methodenkompetenz lässt sich leichter verbessern als soziale Skills	q
1642	hohe Methodenkompetenz, wie in Ausschreibung beschrieben	q	hohe Methodenkompetenz, wie in Ausschreibung beschrieben	q	hohe Methodenkompetenz, wie in Ausschreibung beschrieben	q
1652	KI empfehlung,	k	berufserfahrung und methodenkompetenz, sprache höher als bei der konkurrierenden person, KI empfehlung	q, k	gute methodenkomp & Berufserfahrung, KI Empfehlung	q, k
1663	KI empfehlung, mehr soziale Skills und berufserfahurng	q, k	Ki empfehlung, mehr methoden kompetenz und berufserfahurng	q, k	Ki empfehlung und hohe methodenkompetenz	q, k
1681	Der Bewerber hat mehr Berufserfahrung und zusätzlich bessere soziale Fähigkeiten	q	Die Berufserfahrung und die Methodenkompetenz sind höher als bei der Mitbewerberin	q	Die Methodenkompetenz ist wesentlich höher. auch eine solide Berufserfahrung ist vorhanden	q
1684	höherer Abschluss, und geringeres Alter	q	Ausgewogenere Verteilung der Fähigkeiten;Jünger	q, a	anderer Bewerber nur geringe Methodenkompetenz	q
1690	Erfahrung und Soziale Skills sind wichtiger als ein Top Abschluss	q	Kompetenter	o	Kompetenter	o
1694	Sehr guter Abschluss und Methodenkompetenz	q	Abschluss und Berufserfahrung überwiegen.	q	Gute Methodenkompetenz.	q
1711	Berufserfahrung und Soziale Skills überzeugen	q	überall gut abgeschnitten	q	Methodenkompetenz entscheidend für diesen Bewerber	q

1717	besserer Allrounder	o	methodenkompetenz ist verbesserungswürdig, aber kann man besser erlernen als soziale skills	q	methodenkompetenz lässt sich leichter erlernen als soziale skils	q
1726	KI-Empfehlung	k	BErufserfahrung, Methodenkompetenz	q	Berufserfahrung, Methodenkompetenz	q
1748	Methodenkompetenz	q	Berufserfahrung und Methodenkompetenz	q	Methodenkompetenz.	q
1755	soziale Skills im Projektmanagement wichtig	q	auch hier lässt sich Berufserfahrung erleben, soziale Skills nur schwer	q	fehlende Berufserfahrung im jungen alter nicht sehr relevant	q, a
1769	Berufserfahrung, Soziale Skills besser	q	mehr Erfahrung	q	Berufserfahrung &Methodenkompetenz besser (hoch relevant)	q
1772	Berufserfahrung	q	Erfahrung	q	social	q
1785	Die beiden Bewerber sind sich sehr ähnlich habe insgesamt gleich viele Sterne über die Kategorien. Der Punkt, warum ich mich für die Person entscheiden habe, liegt am Alter, da es perspektivisch für das Unternehmen mehr potential hätte.	q, a	Auch hier, beide Bewerber sind sich sehr ähnlich und ich habe mich auf Grund des Alters und das Potential für die Zukunft entschieden.	q	Beide Bewerber haben gleich viele Sterne insgesamt. Aber der Ausschlaggebende Punk waren hier die große Differenz in der Methodenkompetenz. Eine Differenz von 3 Sternen bei einer wichtigen Fähigkeit ist dann doch zu groß. Und er wird auch von der KI empfohlen.	q, k
1794	Soziale Skills	q	Soziale Skills > Methodenkompetenz, Mitbewerberin steht fast vor Renteneintritt.	q, a	Zu wenig Methodenkompetenz beim Mitbewerber, insb. für eine Führungsrolle hat der Rest dann nicht gereicht	q
1805	ist kompetenter	o	Empfehlung	k	Empfehlung	k
1821	Empfehlung	k	KI	k	Auch wenn er älter ist ist er vermutlich besser (KI)	q, k, a
1822	gut	o	wichtig	o	methoden	q
1837	gute soziale Skills und Berufserfahrung	q	jünger, gute soziale Skills	q, a	gute Methodenkompetenz	q
1844	Mehr Sozialkompetenz	q	Mehr Soziale Skills	q	Mehr Soziale Skills	q
1852	mehr Berufserfahrung und soziale Skills, nur geringer Unterschied in Methodenkompetenz	q	bessere soziale Skills und jünger	q, a	anderer Bewerber hatte nur sehr geringe Methodenkompetenz	q
1855	Methodenkompetenz besser	q	Jünger	a	Methodenkompetenz besser	q
			**Condition 4 – Job 3**			
1336	Mehr Softwareerfahrung und Besserer Abschluss gleichen geringere Berufserfahrung m.E. aus, lassen auf Entwicklungspotential schließen	q	Sprache in diesem Bereich nicht so wichtig; Abschluss und Berufserfahrung gleichwertig o.besser	q	KI Empfehlung, sonst würde ich vermutlich keine der Kandidat:innen wählen	k
1368	insgesamt besser	o	mehr softwareerfahung	q	abschluss & software	q
1372	beim anderen Bewerber lag der Schwerpunkt auf den sozialen Skills. Hier ist eine gute Mischung von allem dabei.	q	Finde soziale Skills soltten mindestens 3 Sterne sein, da Umgang unter den Kollegen auch wichtig ist. Außerdem bringt sie auch sont alles mit außer Softwarekenntnisse könnten verbessert werden.	q	Konkurrent hat zu wenig Berufserfahrung und zu wenig Soziale Skills, Bewerberin hat das bessere Gesamtpaket	q
1395	Sprache, Software, Abschluss	q	Software	q	Abschluss, Software	q
1396	keine Angabe	x	Der Abschluss muss nicht immer eine Rolle spielen	q	Mehr Berufserfahrung sowie soziale Skills	q

1413	besserer Abschluss und Softwarekenntnisse, soziale Skills keine Priorität für den Job	q	bessere Softwareerfahrung, ansonsten fast gleich gute q	q	Berufserfahrung geringer, aber besserer Abschluss und Softwareerfahrung	q
1420	Kandidatin scheint besser zu sein; KI Empfehlung	o, k	KI Empfehlung, Kandidaten sahen ähnlich aus	k	Kandidat hat mehr Sterne in Software Erfahrung und Abschluss	q
1440	KI-Empfehlung, mehr Sterne	k	KI-Empfehlung, mehr Sterne	k	KI-Empfehlung	k
1459	Allgemein besser	o	Beide eig. gleich gut aber man muss sich entscheiden	q	Ausgeglichenere Erfahrung	q
1475	Tendenziell bessere Kompetenz	o	KI Empfehlung	k	KI Empfehlung	k
1502	Frau Decker hat den besseren Abschluss und mehr Softwareerfahrung.	q	Frau Richter schneidet in den Kategorien Abschluss, Berufserfahrung und soziale Skills besser ab	q	Frau Ostermann hat mehr Erfahrung und deutlich bessere Soziale Skills. Ihr Abschluss und die Softwareerfahrung ist wenig schlechter in der Bewertung und beides nicht in einem inakzeptablen Bereich	q
1504	höhere soziale skills	q	höhere sprachkompetenz	q	mehr berufserfahrung	q
1512	KI Empfehlung	k	bbessere bewertung außer Abschluss	q	Besser bzgl. Sprachen, Abschluss und Software	q
1523	mehr Erfahrung im Bereich software	q	bessere soziale Skills	q	mehr Erfahrung im Bereich Software	q
1531	Gesamtpaket	q	Gesamtpaket	o	Softwareerfahrung	q
1538	social skills irrelevant, Geringere Berufserfahrung wird kompensiert durch höhere Softwareerfahrung und gutem Abschluss.	q	Ähnliches Kompetenzniveau, kleiner Vorteil durch höhere Softwareerfahrung.	q	social skills für stelle irrelevant, Softwareerfahrung und Abschluss überzeugen. Einzig Berufserfahrung ausbaubar.	q
1542	besserer abschluss, bessere softwareerfahrung	q	mehr softwareerfahrung	q	wenig berufserfahrung aber gute softwareskills	q
1558	Softwareerfahrung	q	sehr ähnliche Skills, daher Entscheidung bei Software	q	KI-Empfehlung	k
1565	Erscheint qualifiziert	o	Frauenquote erhöhen	e, g	Frauenquote erhöhen	e, g
1570	Mehr Softwareerfahrung,wichtigerfür Buchhaltung	q	Mehr Software & Berufserfahrung, für den Bereich wichtiger	q	Empfehlung derKI, rest war beides ok	q, k
1581	mehr Punkte bei Berufserfahrung und soz. Skills	q	bessere Softwareerfahrung	q	mehr Berufserfahrung	q
1588	Besserer Abschluss	q	höherer Abschluss und Berufserfahrung	q	Mehr Berufserfahrung	q
1617	Mehr Software Erfahrung	q	mehr software erfahrung	q	mehr berufserfahrung	q
1619	mehr Sprachen - KI-Empfehlung - höherer Abschluss	q, k	KI-Empfehlung - höherer Abschluss	q, k	mehr Sprachen - höherer Abschluss - mehr Softwareerfahrung	q
1627	Soziale skills sind hier nicht ausschlaggebend	q	Sehr ähnlich aber jünger und besserer Abschluss	q, a	Berufserfahrung muss nicht ausschlaggebend sein, dafür bessere Softwareerfahrung	q
1641	besserer Abschluss und Softwareerfahrung	q	Unterschiede von je nur einem Punkt, dann der KI	q, k	würde mich jetzt eigentlich gerne umentscheiden, geht leider nicht	x
1642	mehr Berufserfahrung	q	Besserer Abschluss & Berufserfahrung	q	Berufserfahrung scheint mir bei dieser Stelle als besonders relevant	q

1652	bessere Attribute, welche für die Buchhaltung wichtiger sind als für andere bereiche, KI Empfehlung	q, k	Berufserfahrung, sprachen und softwareerfahrung höher als bei der anderen person, abschluss zwar niedriger aber immernoch überdurchschnittlich	q	KI Empfehlung	k
1663	mehr Berufserfahurng und soziale Fähigkeiten	q	Besserer Abschluss und soziale Fähigkeiten bei mehr Berufserfahurng	q	höhere soziale Skills+ KI empfehlung	q, k
1681	Die Softwareerfahrung ist höher, somit könnte die Einarbeitung nicht so lange dauern	q	Die Softwareerfahrung ist höher	q	Die Berufserfahrung ist höher, das kann die fehlende Softwareerfahrung ausgleichen	q
1684	mehr Erfahrung in relevante Positionen (Softskills in diesem Bereich m.E. nicht sehr relevant)	q	würde beide einladen. Sehr ähnlich	q	höherer Abschluss; mehr Softwareerfahrung	q
1690	Kompetenter	o	Besser außer soziale Skills, die hier nicht so bedeutend sind	q	Mehr softwareerfahrung und besserer Abschluss	q
1694	Besserer Abschluss.	q	Besserer Abschluss.	q	Besseres Gesamtergebnis	o
1711	Überall mittemäßig, anderer Bewerber ggf. zu gesprächig, da sehr hoch in Sozialen Skills	q	Softwareerfahrung kann erlernt werden, Rest gut	q	anderer Bewerber wenig Berufserfahrung und wenige soziale Skills	q
1717	bessere softwareerfahrung, soziale skills sind hier nicht so wichtig	q	mehr softwareerfahrung, gleiche berufserfahrung	q	bessere sternebewertung außer bei der berufserfahrung, was aber nicht so schlimm ist	q
1726	mehr Berufserfahrung	q	KI-Empfehlung	k	mehr Berufserfahrung	q
1748	Soziale Skills	q	Abschluss.	q	Soziale Skills	q
1755	super Abschluss und mehr Sprach- und Softwarekenntnis	q	top Abschluss und viel Berufserfahrung. Sprache in der Buchhaltung eher unwichtig	q	ein wenig mehr Berufserfahrung und deutlich höhere soziale Skills	q
1769	Softwareerfahrung + Abschluss besser	q	Abschluss nicht so relevant vergl. mit Softwareerfahrung	q	ausgeglicheneres Profil	o
1772	Softwareerfahrung	q	Softwareerfahrung	q	Skills	o
1785	Sind fast gleich, habe hier auf die KI gehört	k	Sind gleich was die Sterne angeht aber sie ist jünger und von der KI empfohlen	a, k	Sind fast gleich auch vom alter, aber von Ki empfohlen und mehr Eerufserfahrung	q
1794	Sprachen sind in der Buchhaltung nicht so wichtig, Berufserfahrung war hier mehr gegeben und starke soziale Skills. Softwareerfahrung kann erlangt werden.	q	Sprachen nicht so wichtig, Abschluss zweitrangig. Bei gleicher Berufserfahrung mehr soziale Skills. Weniger Softwareerfahrung ist verschmerzbar.	q	Mehr Soziale Skills und mehr Berufserfahrung.	q
1805	ist besser	o	Empfehlung, ist geeigneter	o, k	Ist geeigneter	o
1821	KI	k	KI	k	KI	k
1822	Programmieren	q	KI	k	KI	k
1837	mehr Berufserfahrung, mehr soziale Skills	q	bessere soziale Skills	q	besserer Abschluss	q
1844	Mehr Berufs- und Softwareerfahrungen	q	Mehr Softwareerfahrungen	q	Höherer Abschluss und mehr Softwareerfahrungen	q
1852	KI-Empfehlung	k	Softwareerfahrung stärker gewichtet als soziale Skills und Abschluss, wobei Abschluss bei beiden gut	q	KI-Empfehlung	k
1855	Softwareerfahrung besse	q	bessere Softwareerfahrung	q	bessere Softwareerfahrung	q
